# Exclusion of Notch from the contact site during efferocytosis restricts anticancer immunity

**DOI:** 10.1038/s41590-026-02452-3

**Published:** 2026-03-03

**Authors:** Zhenrui Li, Beisi Xu, Piyush Sharma, Cliff Guy, Emilio Boada-Romero, Katherine Verbist, Ao Guo, Luigi Mari, Suresh Poudel, Mao Yang, Douglas R. Green

**Affiliations:** 1 Department of Immunology, St. Jude Children’s Research Hospital, Memphis, TN USA; 2 Center for Applied Bioinformatics, St. Jude Children’s Research Hospital, Memphis, TN USA; 3 Present Address: Key Laboratory of Immnunotherapy, Center for Advanced Interdisciplinary Science and Biomedicine of IHM, School of Basic Medical Sciences, Division of Life Sciences and Medicine, University of Science and Technology of China, Hefei, China

**Keywords:** Innate immunity, Tumour immunology, Biochemistry

## Abstract

The clearance of dying cells by phagocytes (efferocytosis) is important for maintenance of tissue homeostasis and the active repression of inflammatory responses but can promote an immunosuppressive tumor microenvironment. Here we show that Notch signaling is suppressed actively during efferocytosis and that activation of this pathway by ectopic expression of the Notch intracellular domain in myeloid cells improves anticancer immunity in mice. Contact with dead cells or IgG-coated surfaces induces the activation of an integrin barrier that excludes Notch from the contact site to prevent it signaling. The formation of this active integrin barrier requires the Rubicon–VPS34 complex, which recruits phospholipase D (PLD) to regulate integrin activation. Ablation of Rubicon in the host or inhibition of PLD increases Notch activation during efferocytosis and improves anticancer immunity in a manner dependent on Notch signaling. These findings identify a regulatory mechanism that restricts Notch signaling during efferocytosis.

## Abstract (web summary)

Here the authors show that during phagocytosis of dying cells anticancer Notch signaling is prevented by exclusion of its receptors on the macrophage at the contact site with the dying cell.

## Main

Efferocytosis, the process of engulfment and clearance of dead cells, has important roles in many physiologic and pathologic phenomena. Cells that die during development and normal homeostatic tissue turnover must be removed without inducing inflammation. Similarly, all stages of tumor growth are characterized by copious amounts of cell death, as initial expression of oncogenes provides selective pressure for tumorigenesis through the induction of apoptosis and an established tumor contains areas of nutrient paucity and hypoxia that kill cells^1^. The clearance of such cells contributes to the immunosuppressive tumor microenvironment (TME). The repair of tissue damage is also associated with efferocytosis^2,3^. In each case, the efferocytosis of apoptotic cells contributes to the inflammatory ‘silence’ of apoptotic cell death^4,5^. This feature of efferocytosis acts as a double-edged sword; on one hand, it is critical to maintain organ health and prevent autoimmunity, while, on the other hand, it facilitates the evasion of immunosurveillance in cancer^6,7^.

Dying cells signal the induction of efferocytosis by externalizing phosphatidylserine (PS), which is directly recognized by PS receptors, such as T cell membrane protein 4 (TIM4), brain-specific angiogenesis inhibitor 1 and Stabilin 2, or through bridging proteins such as Gas6, protein S and MFG-E8 that interact with receptors of the Tyro3/Axl/Mer family or integrins to engage efferocytosis^4,5^. The major effector cells of efferocytosis are professional phagocytes, especially macrophages. Upon efferocytosis, macrophages are programmed, through partially understood mechanisms, toward anti-inflammatory polarization that is characterized by production of interleukin 10 (IL-10) and transforming growth factor-β1, concomitant with activation of nuclear receptor families that regulate genes involved in lipid metabolism and transport^8–11^. This anti-inflammatory polarization of efferocytotic macrophages has been implicated in the regulation of autoinflammatory disease and adaptive immune responses and contributes to suppression of antitumor immunity. Therefore, strategies to therapeutically harness efferocytosis are under active development^6^, leading to clinical trials that have reported some success when combined with checkpoint blockade therapy^12,13^.

Once the dead cell is recognized and engulfed, the phagosome and its contained cell corpse are destined to fuse with lysosomes where the dead cell components are digested. This fusion is promoted by the process of LC3-associated phagocytosis (LAP) involving the conjugation of the autophagic proteins ATG8 (that is, LC3 and Gabarap proteins) by the ATG5–ATG12–ATG16L complex to the single membrane of phagosomes. LAP requires the recruitment of Rubicon (encoded by *Rubcn*) and the class III phosphatidylinositol 3-OH kinase (PI3K) VPS34 complex to the early phagosome, where it catalyzes the formation of the signaling lipid phosphatidylinositol 3-phosphate from phosphatidylinositol^14^. This Rubcn–VPS34 complex is recruited to phagosomes by the engagement of cell surface Toll-like receptors, Fc receptors (FcRs) and the apoptotic cell receptor TIM4 (refs. ^14–16^). Using several tumor graft models and a genetic model of lung adenocarcinoma, we previously found that deficiency in components of the Rubcn–VPS34 complex, specifically in myeloid cells, induced proinflammatory polarization of tumor-associated macrophages (TAMs) and restricted tumor growth^16^. As a result, tumor-infiltrating T lymphocytes were activated in the tumors and the antitumor effect was dependent on T cells in the myeloid Rubcn-deficient animals. However, the mechanism by which the Rubcn–VPS34 complex suppresses the immune response during efferocytosis is not fully understood.

The highly conserved Notch signaling pathway integrates environmental cues from neighboring cell–cell communication and determines cell fate during development. More recently, evidence has been reported that Notch activation in macrophages drives proinflammatory polarization and reprograms metabolism^17–20^. Given its essential physiological function in development and diseases, activation of Notch signaling is highly regulated and requires the interaction of ligands (for example, DLL1/3/4 and Jag1/2 in mammals) with Notch receptors (Notch1–Notch4), which triggers the exposure of proteolytic sites that are sequentially cleaved by ADAM proteases and γ-secretase. The latter cleavage liberates the Notch intracellular domain (NICD) from the plasma membrane, which then translocates into the nucleus where it interacts with the transcription factor, RBPJ, to activate target gene expression^21^. Although Notch ligands and receptors are ubiquitously expressed in cells, little is known about how Notch is regulated during efferocytosis during which apoptotic cells bearing Notch ligands have close interaction with phagocytes.

Here, we report that efferocytosis by wild-type (WT) macrophages suppresses Notch signaling through an active integrin barrier-dependent exclusion of Notch receptors on the macrophage plasma membrane, where engulfment occurs. This active integrin barrier formation requires the Rubcn–VPS34 complex, which recruits phospholipase D (PLD) to mediate integrin activation. Activation of Notch, either by ectopic overexpression of NICD in macrophages or by treatment with PLD inhibitors, promotes proinflammatory polarization of macrophages and enhances antitumor effects, as observed in Rubcn-deficient macrophages. RBPJ ablation in macrophages prevented the antitumor effect observed in Rubcn-deficient mice or mice treated with PLD inhibitors, suggesting that the regulation of Notch signaling during efferocytosis has a critical function in antitumor responses.

## Results

### Efferocytosis by Rubcn-deficient macrophages induces Notch activation

**Fig. 1:**
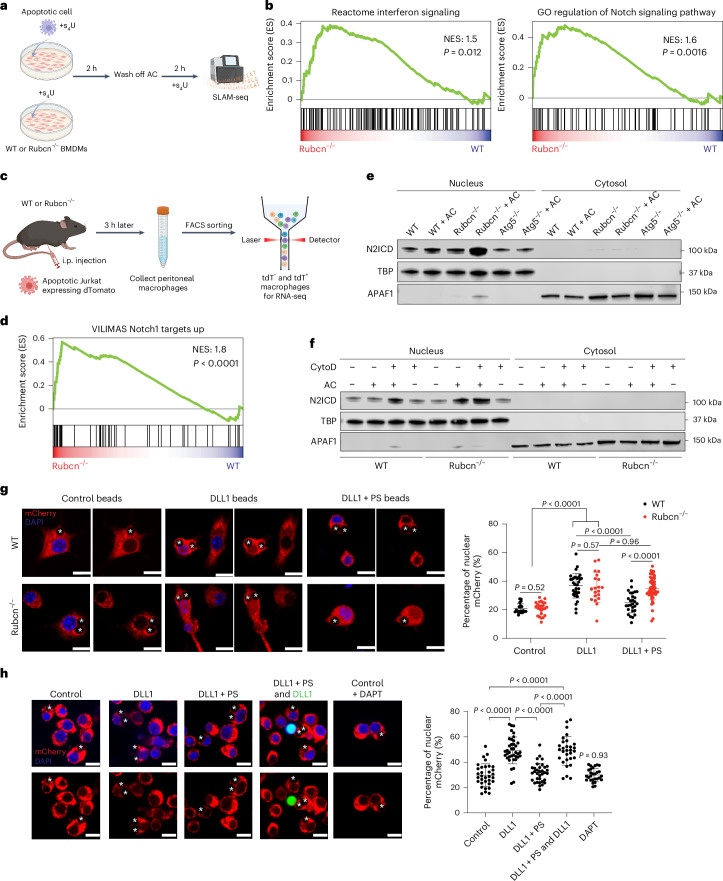
Efferocytosis by Rubcn-deficient macrophages induces Notch activation. **a**, Scheme of SLAM-seq. WT or *Rubcn*^*−/−*^ BMDMs were cultured with 100 μM s^4^U in the presence or absence of apoptotic Jurkat cells and incubated at 37 °C for 2 h. Apoptotic cells (ACs) were then washed off with warm complete medium. Cells were cultured for another 2 h in the presence of 100 μM s^4^U at 37 °C. Total RNA was extracted for library construction and sequencing. **b**, GSEA of IFN signaling and Notch signaling genes in WT and *Rubcn*^*−/−*^ BMDMs upon efferocytosis. GO, Gene Ontology. **c**, Schematic of experimental design. Apoptotic Jurkat cells expressing tdT were injected i.p. into WT and *Rubcn*^*−/−*^ mice. Then, 3 h later, peritoneal macrophages were isolated and subjected to FACS sorting tdT^*−*^ or tdT^+^ (gated on DAPI^*−*^CD45^+^CD11b^+^F4/80^+^) for RNA-seq. **d**, GSEA of upregulated Notch1 target genes in WT and *Rubcn*^*−/−*^ peritoneal macrophages upon efferocytosis as in **c**. **e**, Western blot of nuclear N2ICD in WT, *Rubcn*^*−/−*^ and *Atg5*^*−/−*^ BMDMs that had engulfed apoptotic HT115 cells or not. TATA-binding protein (TBP) and apoptotic peptidase-activating factor1 (APAF1) served as markers for nuclear and cytosolic fractions, respectively. **f**, Western blot of nuclear N2ICD in WT and *Rubcn*^*−/−*^ BMDMs with or without 1 μM CytoD treatment for 3 h upon efferocytosis. **g**, Confocal microscopy imaging of WT and *Rubcn*^*−/−*^ RAW264.7 cells expressing Notch1–mCherry–NLS reporter and TIM4. Cells were cultured with unconjugated (control) beads or beads conjugated with DLL1 ± biotinylated PS for 3 h before fixing and imaging. Activation of Notch1 receptor was quantified by the intensity of mCherry in the nucleus over the total for each cell (100 × nuclear/total). Control group: WT, *n* = 21; *Rubcn*^*−*/*−*^, *n* = 25. DLL1 group: WT, *n* = 33; *Rubcn*^*−*/*−*^, *n* = 20. DLL + PS group: WT, *n* = 28; *Rubcn*^*−*/*−*^, *n* = 52. Statistical analysis was conducted using a Student’s *t*-test. Scale bars, 10 μm. **h**, WT RAW264.7 cells expressing Notch1–mCherry–NLS reporter and TIM4 were cultured with the indicated beads for 3 h before fixing and imaging. For RAW264.7 cells incubated with a mixture of beads coupled with DLL1 plus PS (no color) and DLL1 alone (green), cells that had engulfed or were in close contact with both were quantified as described above. Control, *n* = 33; DLL1, *n* = 38; DLL1 + PS, *n* = 34; DLL1 + PS and DLL1, *n* = 30; DAPT, *n* = 28. Asterisks indicate engulfed beads. Data are the mean ± s.d. Dots represent single cells. Statistical analysis was conducted using a Student’s *t*-test. Scale bars, 10 μm. GSEA significance was calculated using one-sided permutation testing based on a preranked approach. Panels created in BioRender: **a**, Verbist, K. https://biorender.com/jsrbskx (2026); **c**, Verbist, K. https://bioRender.com/q7evtdr (2026). Source data

**Extended Data Fig. 1:**
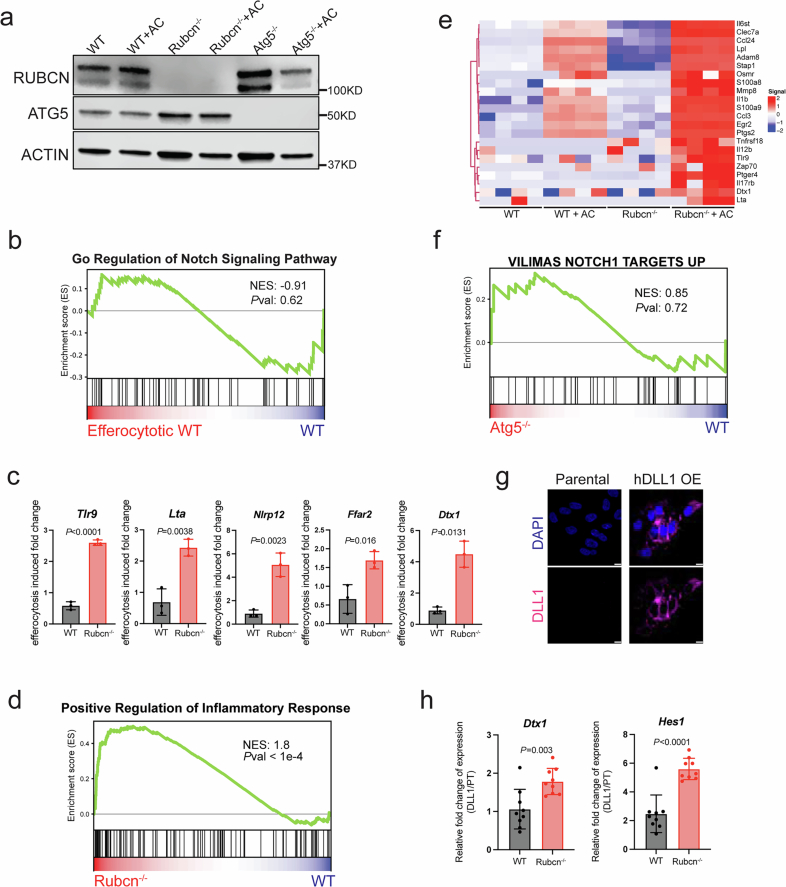
Notch activation in Rubicon-deficient BMDM upon efferocytosis. (**a**) Western blots to validate the ablation of Rubicon and ATG5 in BMDM. (**b**) GSEA of WT peritoneal macrophages with or without efferocytosis of apoptotic Jurkat cells *in vitro* as in Fig. 1a. (**c**) RT-qPCR validation of relative expression of indicated genes in WT and *Rubcn*^*−/−*^ BMDM with or without efferocytosis of apoptotic Jurkat cells *in vitro*. WT (n = 3) and *Rubcn*^*−*/*−*^(n = 3). Data are mean±s.d. Student’s *t* test. (**d**) GSEA analysis of positive regulation of inflammatory response genes in WT and *Rubcn*^*−*/*−*^ peritoneal macrophages upon efferocytosis as in Fig. 1c. (**e**) Heatmap showing genes upregulated in *Rubcn*^*−/−*^ BMDM compared to those in WT BMDM during efferocytosis. (**f**) RNA-seq profiling of Notch target genes in WT or *Atg5*^*−/−*^ BMDM following efferocytosis of apoptotic Jurkat cells. (**g**) Representative images showing DLL1 expression in CHO-K1 cells without (parental) and with ectopically expressed human DLL1. Scale bars, 10 μm. (**h**) RT-qPCR analysis of Notch target genes in WT and *Rubcn*^*−/−*^ peritoneal macrophages that had engulfed apoptotic parental or DLL1-expressing CHO-K1 cells. WT (n = 9) and *Rubcn*^*−*/*−*^(n = 9). Data are mean±s.d. Student’s *t* test. GSEA significance was calculated using one-sided permutation testing based on a pre-ranked approach. Source data

To investigate how defective Rubcn expression influences the response to the engulfment of dying cells, we cocultured apoptotic Jurkat cells with bone-marrow-derived macrophages (BMDMs) from *Rubcn*^*−/−*^ mice and subjected macrophages to SLAM-seq^22^ to identify newly expressed mRNA (Fig. 1a). Consistent with previous observations^16^, we found that BMDM deficient in Rubcn displayed an increased interferon (IFN) response. Interestingly, Notch signaling was enriched in *Rubcn*^*−/−*^ BMDMs upon efferocytosis compared to WT BMDMs that had engulfed apoptotic cells (Fig. 1b and Extended Data Fig. 1a–c). To test the effect of Rubcn deficiency in macrophages in vivo, we injected tdTomato (tdT)-expressing apoptotic Jurkat cells intraperitoneally (i.p.) into WT or *Rubcn*^*−/−*^ mice, then sorted tdT^*−*^ and tdT^+^ peritoneal macrophages and subjected them to RNA sequencing (RNA-seq) (Fig. 1c). Again, we observed that dead cell engulfment by Rubcn-deficient but not WT macrophages induced inflammatory and Notch signaling gene sets (Fig. 1d and Extended Data Fig. 1d,e). This increase in Notch signaling was not observed in ATG5-deficient BMDMs that had engulfed apoptotic Jurkat cells (Extended Data Fig. 1a,f). To further examine Notch activation in Rubcn-deficient macrophages in vivo, we i.p. injected apoptotic parental CHO-K1 cells that do not express endogenous Notch ligands^23^ and CHO-K1 cells ectopically expressing human DLL1 (Extended Data Fig. 1g) into WT and *Rubcn*^*−/−*^ mice. We sorted efferocytotic peritoneal macrophages that engulfed live/dead red-labeled apoptotic CHO-K1 cells for reverse transcription (RT)–qPCR and found that Notch target genes, *Dtx1* and *Hes1*, were significantly upregulated in Rubcn-deficient macrophages that had engulfed apoptotic CHO-K1 cells expressing human DLL1 compared to those that had engulfed parental CHO-K1 cells (Extended Data Fig. 1h).

**Extended Data Fig. 2:**
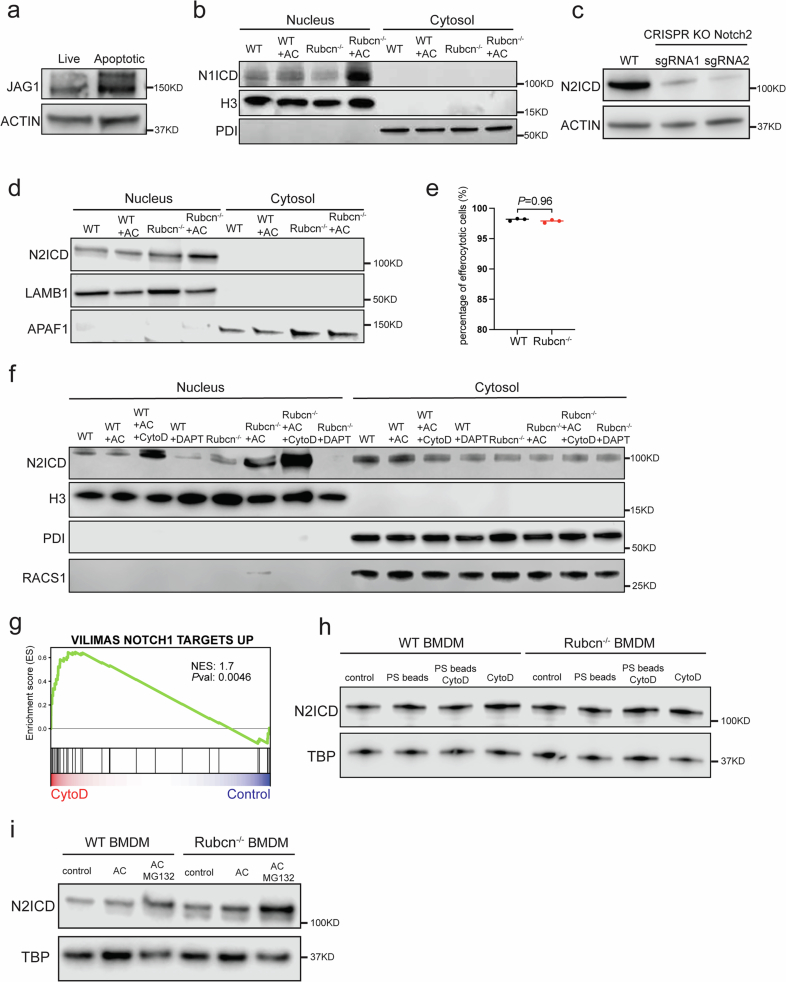
Notch receptor is proteolytically activated in Rubicon-deficient macrophages during efferocytosis. (**a**) Western blot of JAG1 in live and apoptotic Jurkat cells (5 *μ*M ABT-737 and 5 μM S63845 treatment for 45 mins). (**b**) Immunoblot of nuclear N1ICD in WT, *Rubcn*^*−/−*^ BMDM that had engulfed apoptotic Jurkat cells. Histone3 H3 (H3) and protein disulfide isomerase (PDI) were used as nuclei and ER markers, respectively. (**c**) Immunoblot of Notch2 in Jurkat cells stably expressing sgRNAs targeting Notch2. (**d**) Western blot of nuclear N2ICD in WT or *Rubcn*^*−/−*^ BMDM that had engulfed apoptotic Notch2-deficient Jurkat cells as in (**c**). (**e**) Quantification of efferocytosis efficiency in WT or *Rubcn*^*−/−*^ BMDM cultured with apoptotic Jurkat cells expressing tdTomato at 37 °C for 3 hours. Apoptotic Jurkat cells were washed off, and the BMDM were collected and stained with DAPI. The efferocytosis efficiency was determined as the frequency of DAPI^*−*^, tdTomato^+^ cells by flow cytometric analysis. WT (n = 3) and *Rubcn*^*−*/*−*^(n = 3). Student’s *t* test. (**f**) Western blot of nuclear N2ICD in WT or *Rubcn*^*−/−*^ BMDM with or without 1 μM CytoD treatment during 3-hours of efferocytosis of apoptotic Jurkat cells. For DAPT treatment, cells were pretreated with 1 μM DAPT overnight before the experiment. RACS1 was used as a Golgi marker. (**g**) GSEA analysis of RNAseq using WT BMDM cultured with apoptotic Jurkat cells in the presence of DMSO or 1 μM CytoD for 3 hours. (**h**) Immunoblot of N2ICD in WT and *Rubcn*^*−/−*^ BMDM that had been co-cultured with PS-coated beads in the presence or absence of 1 μM CytoD. (**i**) Immunoblot of N2ICD in WT and *Rubcn*^*−/−*^ BMDM that had been co-cultured with apoptotic Jurkat cells in the presence or absence of 20 μM MG132 for 3 hours. AC, apoptotic cells. GSEA significance was calculated using one-sided permutation testing based on a pre-ranked approach. Source data

Notch receptors are ubiquitously expressed in immune cells and we observed a similar level of the Notch ligand, Jagged1 (JAG1), in living and apoptotic cells (Extended Data Fig. 2a). We tested whether efferocytosis by Rubcn-deficient BMDMs induced proteolytic activation of the Notch receptor by coculturing apoptotic HT115 cells that express Notch ligands *JAG1* and *JAG2* (The Human Protein Atlas) with BMDMs and performed nuclear fractionation to assess the translocation of the N2ICD by immunoblotting. Rubcn-deficient BMDMs that had engulfed apoptotic cells showed increased nuclear N2ICD as compared to WT or *Atg5*^*−/−*^ BMDMs that had engaged efferocytosis (Fig. 1e). We also observed increased nuclear translocation of the N1ICD in efferocytotic *Rubcn*^*−/−*^ BMDMs compared to WT BMDMs (Extended Data Fig. 2b). To exclude possible contamination of N2ICD from apoptotic cells, we repeated this experiment with apoptotic Jurkat cells in which *Notch2* was ablated using CRISPR–Cas9 (Extended Data Fig. 2c). Again, we found that engulfment of apoptotic cells lacking Notch2 by *Rubcn*^*−/−*^ BMDMs induced higher nuclear N2ICD compared to that in WT BMDMs (Extended Data Fig. 2d). This was not attributable to a lower efferocytosis efficiency in *Rubcn*^*−/−*^ BMDMs (Extended Data Fig. 2e). In summary, these data suggest that Rubcn deficiency leads to increased Notch activation in macrophages during efferocytosis.

We asked whether efferocytosis has a role in the suppression of Notch receptor activation. We cocultured apoptotic cells with BMDMs in the presence of, cytochalasin D (CytoD), which inhibits actin polymerization and, thus, effercytosis^24^. While *Rubcn*^*−/−*^ BMDMs displayed a higher nuclear N2ICD upon feeding apoptotic cells in the presence or absence of CytoD, blocking efferocytosis increased N2ICD nuclear translocation in WT BMDMs that were cocultured with apoptotic cells (Fig. 1f and Extended Data Fig. 2f). Bulk RNA-seq analysis also revealed that CytoD treatment increased Notch signaling activation during efferocytosis in WT BMDMs (Extended Data Fig. 2g), suggesting that Notch signaling was suppressed during efferocytosis in WT macrophages. In contrast, the presence or absence of CytoD in BMDMs cocultured with PS-coated beads (mimicking apoptotic cells but without Notch ligands) had no effect on nuclear N2ICD in WT or *Rubcn*^*−/−*^ BMDMs (Extended Data Fig. 2h).

The stability of NICD is regulated by ubiquitinylation and degradation by the proteasome^21,25^. We, therefore, asked whether a defect in Rubcn results in impaired degradation of NICD. We found that, although the proteasome inhibitor MG132 led to slightly increased nuclear N2ICD in WT BMDMs that had engulfed dead cells, Rubcn-deficient BMDMs treated with MG132 showed a higher level of nuclear N2ICD compared to that of WT BMDMs upon efferocytosis (Extended Data Fig. 2i), suggesting that the activation of Notch receptor in *Rubcn*^*−/−*^ BMDMs could not be attributed to reduced NICD degradation.

**Extended Data Fig. 3:**
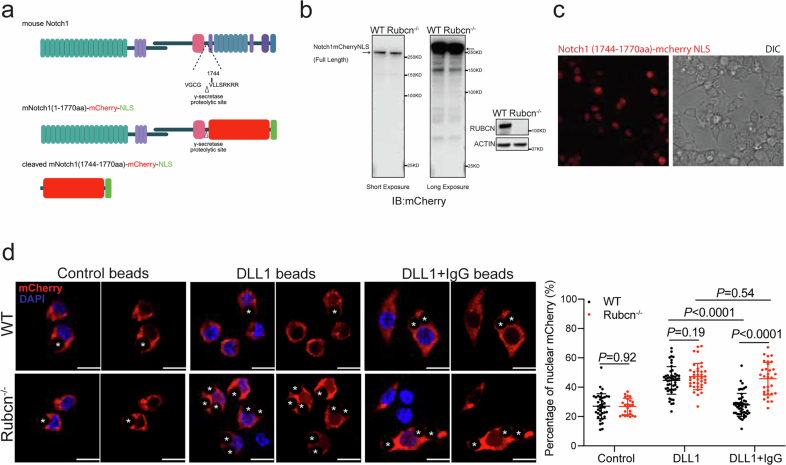
Rubicon is required for the inhibition of Notch activation mediated by IgG-FcR. (**a**) Schematic showing the design of the Notch1-mCherry-NLS reporter. Murine Notch1 is cleaved at amino acid 1744 by *γ*-secretase. To make the Notch activation reporter, murine Notch1 (1-1770aa) was fused to mCherry with a C-terminal nuclear localization sequence (NLS), so the activation of Notch induces the translocation of cleaved mCherry to the nucleus. (**b**) Immunoblot of Notch1-mCherry-NLS expression in WT and Rubicon-deficient Raw264.7 cells with anti-mCherry antibody. (**c**) Confocal microscopy image of murine Notch1 (1744-1770aa)-mCherry-NLS fusion protein stably expressed in RAW264.7 cells. DIC, differential interference contrast. (**d**) Confocal microscopy imaging of WT and *Rubcn*^*−/−*^ RAW264.7 cells expressing Notch1-mCherry-NS reporter. Cells were cultured with unconjugated (control) beads or beads conjugated with DLL1±biotin-mouse IgG1 Fc for 3 hours before fixing and imaging. Activation of Notch1 receptor was quantified by the intensity of mCherry in the nucleus over total for each cell (100 x nuclear/total). Control group: WT(n = 38), *Rubcn*^*−*/*−*^(n = 24); DLL1 group: WT(n = 50), *Rubcn*^*−*/*−*^(n = 38); DLL+IgG group: WT(n = 51), *Rubcn*^*−*/*−*^(n = 32). Results of two independent experiments were combined. *indicates engulfed beads. Each dot represents a single cell. Data are mean±s.d. Student’s *t* test. Scale bars, 10 μm. Illustrations in **a** created in BioRender; Verbist, K. https://biorender.com/4yjlad1 (2026). Source data

We generated a reporter in which murine Notch1 (amino acids 1–1770) was fused to mCherry with a C-terminal nuclear localization sequence (NLS) (Extended Data Fig. 3a,b). Upon Notch ligation, γ-secretase cleaves Notch at amino acid 1744 (ref. ^26^), liberating the intracellular region, which leads to nuclear localization of mCherry in cells expressing our reporter, indicating Notch signaling (Extended Data Fig. 3c). We treated parental and Rubcn-deficient RAW264.7 cells expressing TIM4, a PS receptor^27^, and our reporter with beads coupled with the Notch ligand DLL1, with or without PS to engage Rubcn recruitment. We found robust mCherry nuclear localization in parental and Rubcn-deficient cells exposed to DLL1-coupled beads and this was suppressed in the parental cells by the presence of PS on the DLL1-coupled beads. In contrast, the presence of PS did not affect nuclear mCherry localization in Rubcn-deficient cells (Fig. 1g). Therefore, the presence of PS inhibited Notch signaling in a manner that depends on Rubcn.

To determine whether PS and DLL1 must be on the same bead to inhibit Notch signaling in parental RAW264.7 cells, we repeated the above experiment, mixing beads conjugated with PS plus DLL1 and beads conjugated with DLL1 alone (Fig. 1h). Again, mCherry nuclear translocation in cells incubated with beads bearing PS plus DLL1 beads was reduced compared to cells incubated with beads bearing only DLL1. However, in parental cells that had engulfed or closely contacted both beads with PS plus DLL1 and beads containing only DLL1, mCherry nuclear translocation was induced at a level similar to cells incubated only with beads bearing DLL1 (Fig. 1h). Therefore, the presence of PS on the same particle bearing Notch ligand is required to suppress Notch activation.

To test whether another phagocytosis stimulus that also recruits Rubcn could similarly suppress Notch signaling, we used beads coated with DLL1 with or without murine IgG Fc fragments to engage FcRs^28^. Again, we found that the presence of IgG suppressed mCherry nuclear localization in parental cells while having no effect in Rubcn-deficient cells (Extended Data Fig. 3d). Thus, Rubcn inhibits Notch signaling during phagocytosis.

### Activation of Notch signaling in macrophages enhances antitumor immunity

**Fig. 2:**
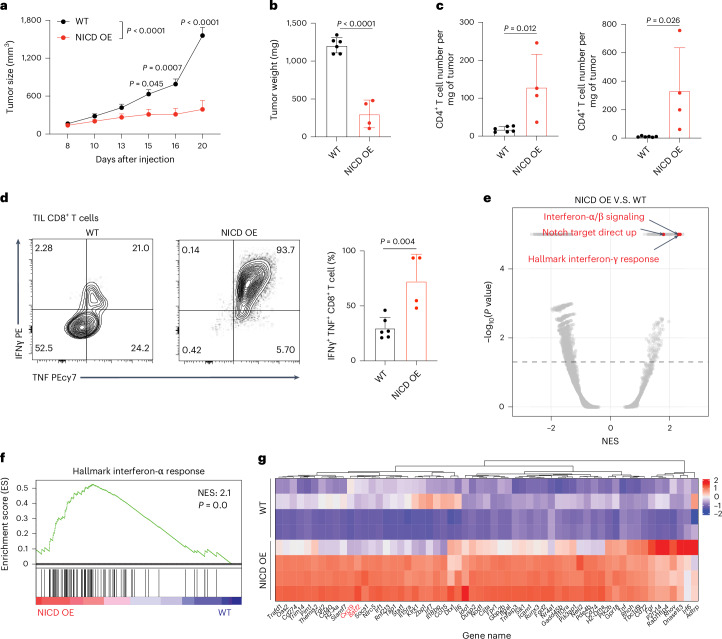
Activation of Notch signaling in macrophages promotes an antitumor effect. **a**, Tumor growth of Yumm1.7 cells in *LysM*-*cre*^−^;*loxp*-*STOP*-*loxp* (*LSL*)-*NICD* (WT, *n* = 9) and *LysM-cre*^+^;*LSL*-*NICD* (NICD overexpression (OE), *n* = 5) mice. All WT mice were littermates of NICD OE mice. Data are the mean ± s.e.m. Statistical analysis was conducted using a two-way ANOVA. **b**, Tumors from mice analyzed in **a** were collected and weighed (*n* = 6 for *cre*^−^ mice; *n* = 4 for *cre*^+^ mice). **c**, Absolute cell number of CD4^+^ T cells and CD8^+^ T cells in each tumor in **b**. **d**, Representative flow cytometry plots and frequency of IFNγ^+^TNF^+^ among tumor-infiltrated CD8^+^ T cells (*n* = 6 for *cre*^*−*^ mice; *n* = 4 for *cre*^+^ mice). **e**, Volcano plot of preranked GSEA results using gene sets from MSigDB against log_2_(fold change) for NICD OE versus WT. The *y* axis is the −log_10_(*P* value) with a pseudovalue of 1 × 10^*−*5^ for 0. The *x* axis is the normalized enrichment score (NES). The horizontal dashed line represents *P* = 0.05. **f**, GSEA of hallmark IFNα response genes in TAMs. **g**, Heat map showing IFNα response genes that are upregulated in TAMs from NICD OE mice. Data are the mean ± s.d. Statistical analysis was conducted using a Student’s *t*-test (**b**–**d**). GSEA significance was calculated using one-sided permutation testing based on a preranked approach. Source data

**Extended Data Fig. 4:**
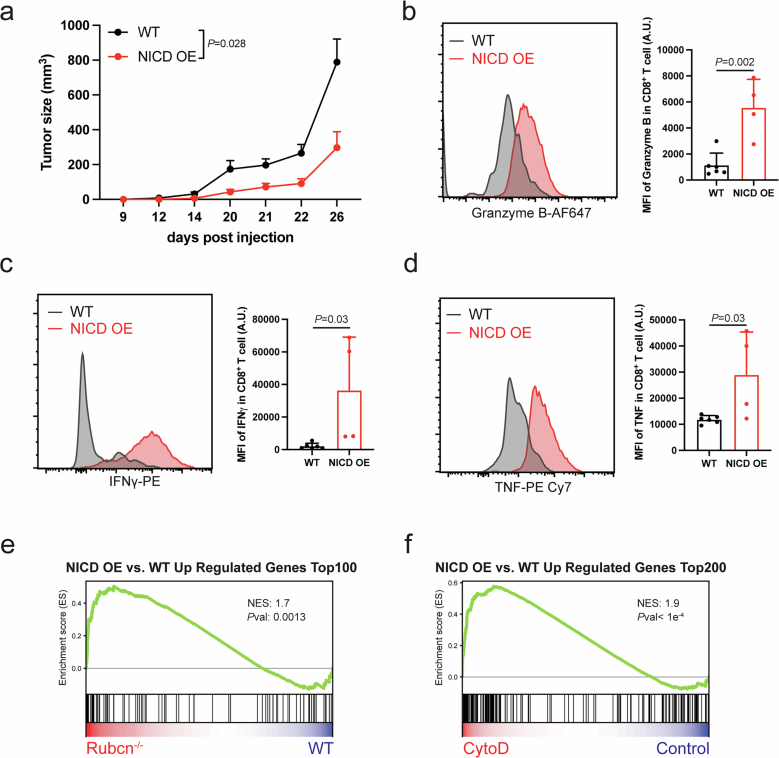
Notch activation in macrophages promotes an anti-tumor effect and associates with enhanced effector function of T cells. (**a**) Growth of B16BL6 melanoma flank tumors in WT (n = 12) and NICD OE (n = 5) mice. Data are mean±s.e.m. (b-d) Representative flow cytometry plots and quantification of MFI of Granzyme B (**b**), IFN*γ* (**c**), and TNF*α* (**d**) in tumor infiltrated CD8^+^ T cells from *LysM-cre*^*−*^*; LSL-NICD* (WT, n = 6) and *LysM-cre*^*+*^*; LSL-NICD* (NICD OE, n = 4) mice as analyzed in Fig. 2a. (**e** and **f**) GSEA of the RNA-seq data from efferocytotic peritoneal macrophages (Fig. 1d, Extended Data Fig. 1d, e) and CytD treated BMDM (Extended Data Fig. 2g) using the gene set upregulated in TAM ectopically expressing NICD. Statistical analysis for tumor growth (**a**) used 2-way ANOVA. Statistical analysis for absolute cell number (b-d) used Student’s *t* test. Data are mean±s.d. GSEA significance was calculated using one-sided permutation testing based on a pre-ranked approach. Source data

Notch activation in macrophages has been shown to drive proinflammatory polarization and IFN responses^29^. We, therefore, asked whether Notch activation in macrophages influences the antitumor response, as we observed in Rubcn-deficient macrophages^16^. We generated mice with activated Notch signaling in myeloid cells by crossing *LysM*-*cre*^+^ and *loxp*-*STOP*-*loxp*-*NICD*, the N1ICD lacking PEST sequences^30^. We observed that the growth of subcutaneously engrafted murine Yumm1.7 cells was suppressed in these animals (Fig. 2a,b). Similar results were obtained with engraftment of B16BL6 cancer cells (Extended Data Fig. 4a). This antitumor effect of activated Notch signaling in myeloid cells was associated with increased T cell infiltration (Fig. 2c). We assessed the tumor-infiltrating CD8^+^ T cells by intracellular staining and found that granzyme B, IFNγ and tumor necrosis factor (TNF) were significantly increased in these T cells in mice with activated Notch signaling in myeloid cells (Fig. 2d and Extended Data Fig. 4b–d).

TAMs represent one of the most abundant immune cell types in the TME and are known to have a critical role in promoting tumor growth by providing an immunosuppressive microenvironment^31^. We previously found that Rubcn-deficient TAMs displayed proinflammatory macrophage polarization associated with increased type I IFN signaling, enhancing the antitumor effect of T cells in the microenvironment^16^. To further explore the differentially regulated genes in TAM with active Notch signaling, we sorted CD45^+^CD11b^+^F4/80^+^Ly-6c^*−*^Ly-6g^*−*^ macrophages from tumors engrafted into mice with or without myeloid expression of NICD and obtained RNA-seq profiles. Gene set enrichment analysis (GSEA) of the differentially expressed genes revealed that Notch target genes and genes associated with IFN signaling were among the most significant changes in Notch-activated macrophages compared to WT macrophages (Fig. 2e,f). Among the IFN response genes upregulated in NICD ectopically expressed in TAMs (Fig. 2g) were *Batf2* and *Cxcl9* that have been reported to associate with increased tumor-infiltrating CD8^+^ T cells^32^ and an anti-TME in human^33^. Further GSEA of the RNA-seq data from efferocytotic peritoneal macrophages (Fig. 1d and Extended Data Fig. 1d,e) and CytoD-treated BMDMs (Extended Data Fig. 2g) revealed that genes upregulated in TAM ectopically expressing NICD were significantly enriched in Rubcn-deficient peritoneal macrophages and CytoD-treated BMDMs during efferocytosis (Extended Data Fig. 4e,f). Therefore, activation of Notch signaling primes macrophages for proinflammatory polarization and enhances antitumor effects.

### Rubcn regulates Notch receptor exclusion during phagocytosis

**Fig. 3:**
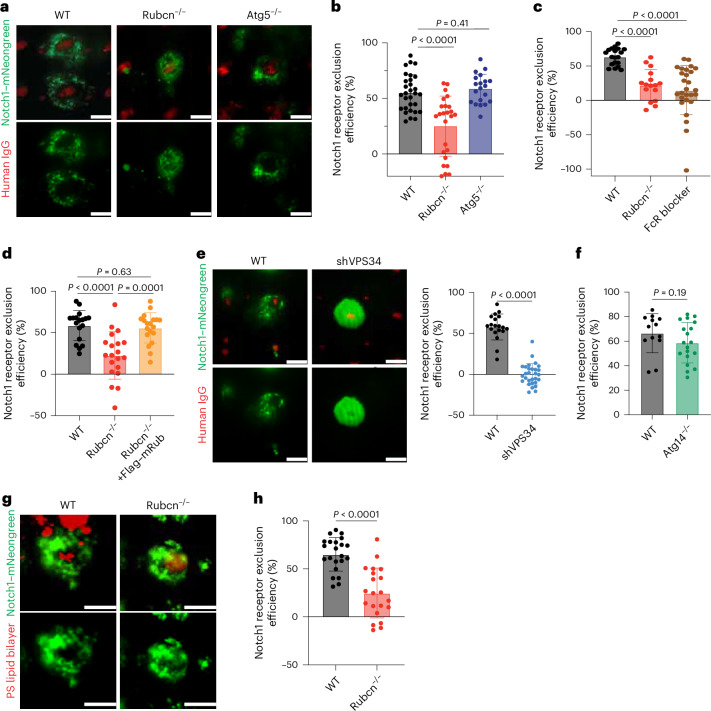
The Rubcn–VPS34 complex is required to exclude Notch. **a**,**b**, Representative TIRF images (**a**) and quantification (**b**) of Notch1–mNeongreen (green) exclusion from areas with IgG Fc (red) in WT (*n* = 30), *Rubcn*^*−/−*^ (*n* = 26) and *ATG5*^*−/−*^ (*n* = 20) THP-1 cells. **c**, THP-1 cells expressing Notch1–mNeongreen were pretreated with Fc blocker and anti-CD32 antibody for 30 min before seeding on arrayed human IgG Fc and cultured for 10 min. Notch1 exclusion was quantified as described in the Methods. Results of two independent experiments were combined. WT, *n* = 19; *Rubcn*^*−/−*^, *n* = 16; WT + FcR blocker, *n* = 27. **d**, Quantification of Notch1 exclusion in WT and *Rubcn*^*−/−*^ THP-1 cells with or without Flag-tagged murine Rubcn. WT, *n* = 18; *Rubcn*^*−/−*^, *n* = 20; *Rubcn*^*−/−*^ with Flag–mRubcn, *n* = 19. **e**,**f**, TIRF imaging of Notch1 exclusion in Dox-treated WT (*n* = 19) or shVPS34 (*n* = 30) expressing THP-1 cells (**e**) or *ATG14*^*−/−*^ THP-1 cells (*n* = 19) (**f**). **g**,**h**, Representative TIRF images (**g**) and quantification (**h**) of Notch–mNeongreen (green) exclusion from contact sites of PS-containing RBC membranes (red) in WT (*n* = 22) and *Rubcn*^*−/−*^ (*n* = 21) THP-1 cells. Results of two independent experiments were combined. Quantification was assessed as (1 − (*X* − *B*/*Y* − *B*)] × 100, where *X* is the MFI of green over red dots, *Y* is the MFI of green dots over whole cells and *B* is the MFI of green dots over background. Each point represents a single cell. Scale bars, 10 μm. Data are the mean ± s.d. Statistical analysis was conducted using a Student’s *t*-test (**b**–**f**,**h**). Source data

**Extended Data Fig. 5:**
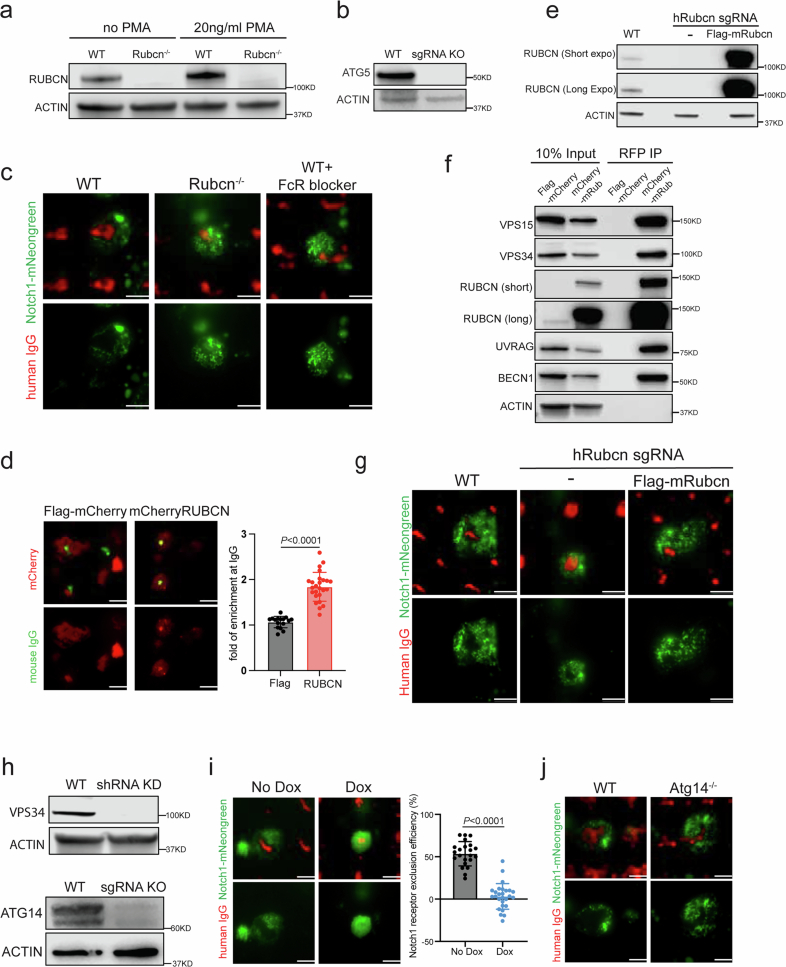
Ectopic expression of murine Rubicon restores Notch exclusion in THP-1 cells lacking endogenous Rubicon. (**a**) Western blot of Rubicon in parental and THP-1 cells stably expressing Cas9 and sgRNA targeting Rubicon. Cells treated with or without 20 ng/ml PMA for 16 hours were lysed for western blot. (**b**) Western blot of ATG5 in parental and THP-1 cells stably expressing Cas9 and sgRNA targeting Atg5 for ablation. (**c**) Representative images for Fig. 3c. THP-1 cells expressing Notch1-mNeongreen were pretreated with Fc blocker and anti-CD32 antibody for 30 mins before seeding on micropatterned human IgG and cultured for 10 mins at 37 °C. (**d**) TIRF images of Raw264.7 cells stably expressing the indicated fusion proteins and quantification of enrichment of Flag-mCherry (n = 16) and mCherry-Rubicon (n = 25) on mouse IgG contact regions. Data are mean±s.d. Student’s *t* test. (**e**) Immunoblot of Rubicon in parental and THP-1 cells with ablation of endogenous Rubicon as shown in (**a**) with or without expression of Flag tagged murine Rubicon (Flag-mRubcn). (**f**) THP-1 cells stably expressing Flag-mCherry or mCherry-tagged murine Rubicon (mCherry-mRubcn) were lysed and subjected to RFP-TRAP-immunoprecipitation (IP) and immunoblotted. 10% of total lysates served as input for IP. (**g**) Representative TIRF images of Notch1 exclusion in WT or *Rubcn*^*−/−*^ THP-1 cells with or without Flag-tagged murine Rubicon as in Fig. 3d. (**h**) Western blot of VPS34 and ATG14 in parental THP-1 cells and THP-1 cells expressing shVPS34 or sgRNAs targeting *Atg14*. (**i**) TIRF images and quantification of Notch exclusion in shVPS34 THP-1 cells with or without Dox treatment to induce the expression of shRNA against *Vps34*. Results of two independent experiments were combined. No dox group (n = 22), Dox group (n = 27). Data are mean±s.d. Student’s *t* test. (**j**) Representative images of Fig. 3f. TIRF images of Notch1-mNeongreen in WT and *Atg14*^*−/−*^ THP-1 cells cultured on human IgG dots. Each dot represents a single cell. Scale bars, 10 μm. Source data

We observed the expression of Notch ligand on apoptotic cells (Extended Data Fig. 2a). It remained a question of how Notch activation was suppressed by efferocytosis. A report demonstrated that opsonization of particles (for example, with antibodies) causes active integrins to form a diffusion barrier that induces an ectodomain size-dependent exclusion of CD45 (ref. ^34^). We reasoned that, as the ectodomain of Notch (over 1,400 aa) is much larger than that of CD45 (approximately 566 aa), such a diffusion barrier would similarly exclude Notch, preventing Notch ligands on the particle (for example, IgG bead, PS or apoptotic cell) from engaging Notch. To test this idea, we used a system^34^, in which small ‘dots’ of human IgG (engaging FcRs) were micropatterned on glass slides and then cultured with THP-1-derived macrophages. Notch1 was assessed by the expression of Notch1 fused to mNeonGreen (green) and imaged by total internal reflection fluorescence (TIRF) illumination. In parental THP-1 cells, Notch was excluded from the IgG dot within minutes of interaction. In contrast, IgG binding did not exclude Notch to the same extent when Rubcn was ablated (Fig. 3a,b and Extended Data Fig. 5a). In contrast, ATG5 ablation in THP-1 cells had no effect on Notch1 exclusion (Fig. 3a,b and Extended Data Fig. 5b). Pretreatment of THP-1 cells with FcR blocker to abrogate the interaction of micropatterned human IgG with FcRs resulted in reduced Notch exclusion (Fig. 3c and Extended Data Fig. 5c).

Rubcn is rapidly recruited to the plasma membrane by IgG-beads^15^ and we similarly observed Rubcn recruitment to micropatterned IgG (Extended Data Fig. 5d). We then ectopically expressed mouse Rubcn in THP-1 cells with or without endogenous Rubcn (Extended Data Fig. 5e,f). Consistent with previous observations^15,35,36^, mouse Rubcn coprecipitated with VPS34, VPS15 and Beclin1, components of the class III PI3K complex (Extended Data Fig. 5f), indicating that murine Rubcn was functional in THP-1 cells. The THP-1 cells lacking endogenous Rubcn and reconstituted with mouse Rubcn displayed a similar efficiency in Notch exclusion to that of parental cells (Fig. 3d and Extended Data Fig. 5e,g).

To further explore how Rubcn regulates Notch exclusion, we ablated VPS34, the catalytic subunit of the PI3K complex, by doxycycline (Dox)-inducible expression of short hairpin RNA (shRNA) in THP-1 cells expressing our Notch1–mNeongreen fusion reporter (Extended Data Fig. 5h). In parental cells treated with Dox, Notch was excluded from the IgG dot, whereas silencing VPS34 disrupted Notch exclusion (Fig. 3e and Extended Data Fig. 5i). ATG14 forms a class III PI3K complex with VPS34 lacking Rubcn, which is essential for autophagy but not LAP^14^. We ablated ATG14 in THP-1 cells (Extended Data Fig. 5h) and cultured these cells on micropatterned human IgG. We observed a similar efficiency in Notch exclusion in ATG14-deficient cells and in WT cells (Fig. 3f and Extended Data Fig. 5j). These findings suggest that the Rubcn–VPS34 complex but not the ATG14–VPS34 complex functions in the exclusion of Notch from the contact site.

**Extended Data Fig. 6:**
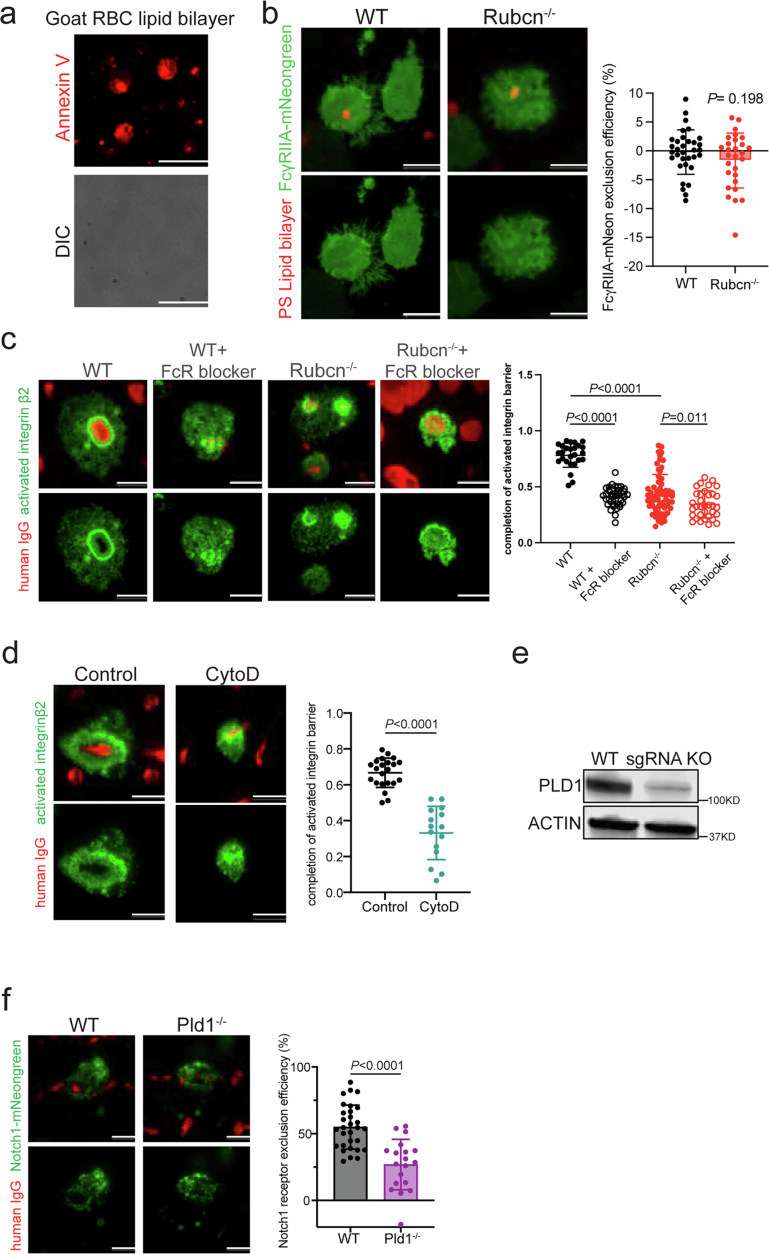
An active integrin barrier excludes Notch. (**a**) Representative confocal images of goat red blood cells (RBC) lysed by hypotonic pressure and stained with Alexa Fluor647 conjugated Annexin V to visualize PS exposure. Differential interference contrast (DIC) shows lipid bilayers but not whole cells remaining on the coverslip. (**b**) TIRF images and quantification of FcγRIIA-mNeongreen exclusion from PS containing lipid bilayer. WT (n = 33), *Rubcn*^*−/−*^ (n = 28) (**c**) TIRF images and quantification of active integrin *β*2 formation in WT and *Rubcn*^*−/−*^ THP-1 cells in the presence or absence of 16 μg/ml anti-CD16/CD32 antibody. Results of two independent experiments were combined. WT (n = 26), WT + FcR blocker (n = 36), *Rubcn*^*−/−*^ (n = 64) and *Rubcn*^*−/−*^ + FcR blocker (n = 34). (**d**) TIRF image of active integrin *β*2 in THP-1 cells. Cells were pre-treated with DMSO (n = 22) or 1 μM CytoD (n = 15) for 1 hour and then cultured on arrayed human IgG dots in the presence or absence of 1 *μ*M CytoD. (**e**) Western blot of PLD1 in parental cell and THP-1 cells stably expressing Cas9 and sgRNA targeting *Pld1*. (**f**) TIRF image of Notch-mNeongreen reporter in WT (n = 30) and PLD1-depleted THP-1 cells (n = 19) cultured on micropatterned human IgG for 10 mins at 37 °C. The WT control in (**f**) is the same as in Fig. 3a since all images were taken from the same experiment. Scale bars, 10 μm. Student’s *t* test (Extended Data Fig. 6b-d and f). Data are mean±s.d. otherwise indicated. Source data

As Rubcn is also recruited upon efferocytosis of dead cells^15,16^, we posited that initiation of efferocytosis would also induce Notch exclusion. To assess this, we made a goat red blood cell (RBC) smear on glass coverslips and burst the RBCs by hypotonic pressure, leaving ~3.5-μm PS-containing lipid bilayers on the coverslip, as assessed by Annexin V staining (Extended Data Fig. 6a). We then cultured THP-1-derived macrophages expressing human TIM4 and our Notch reporter on this preparation for 10 min before TIRF illumination to assess Notch exclusion. In WT control THP-1 cells, Notch was excluded from the lipid bilayers, whereas the exclusion was reduced in Rubcn-deficient THP-1 cells (Fig. 3g,h). In contrast, FcγRIIA, the ectodomain of which (less than 200 aa) is much smaller than that of Notch was not excluded from the PS contact region in either WT or Rubcn-deficient THP-1 cells (Extended Data Fig. 6b). These results, together with the observation that the presence of either PS or IgG on DLL1-coated beads suppressed Notch activation (Fig. 1g and Extended Data Fig. 3d) suggest that Notch exclusion upon the engagement of IgG or PS during phagocytosis relies on the Rubcn–VPS34 complex.

### The Rubcn–VPS34 complex promotes an integrin barrier through PLD

**Fig. 4:**
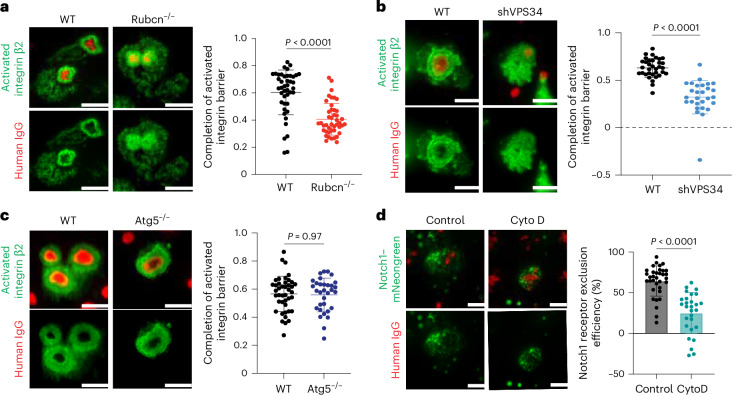
Rubcn promotes activated integrin barrier formation. **a**–**c**, Representative TIRF images (left) and quantification (right) of active β2 integrin staining in WT (*n* = 43) and *Rubcn*^*−/−*^ (*n* = 44) (**a**), VPS34-knockdown (*n* = 28) (**b**) and *ATG5*^*−/−*^ (*n* = 32) (**c**) THP-1 cells that were cultured on arrayed human IgG dots. **d**, TIRF imaging of Notch exclusion. THP-1 cells were pretreated with 1 μM CytoD for 15 min and then seeded on micropatterned human IgG for another 15 min. Control, *n* = 34; CytoD, *n* = 28. Scale bars, 10 μm. Data are the mean ± s.d. Statistical analysis was conducted using a Student’s *t*-test (**a**–**d**). Source data

Integrin activation is linked to phagocytosis that is mediated by IgG–FcR and PS–TIM4 engagement^34,37^. Activated integrin β2 establishes an actin-tethered diffusion barrier that is required to demarcate the CD45 depletion zone at IgG–FcR contact regions^34^. Thus, we asked whether Rubcn participates in this activated integrin barrier formation to exclude Notch. To assess this, we used an antibody that recognizes the active form of human β2 integrin and stained THP-1 cells cultured on slides with micropatterned human IgG. Using TIRF illumination, we observed a ring of activated β2 integrin surrounding the IgG dots. This apparent barrier was reduced in cells lacking Rubcn (Fig. 4a). Preventing the interaction of IgG with FcRs by FcR blocker treatment impaired the formation of activated β2 integrin around IgG dots (Extended Data Fig. 6c). Silencing of VPS34 resulted in the disruption of the activated β2 integrin ring around IgG dots (Fig. 4b). In contrast, ablation of ATG5 in THP-1 cells had no effect on barrier formation around IgG contact regions (Fig. 4c). Thus, activated integrin barrier formation is regulated by the Rubcn–VPS34 complex but not by downstream autophagy signaling.

Upon binding to cognate ligands, integrins are activated and trigger the association of Talin and Kindlin with the actin cytoskeleton. This increases the tensile force on integrins, which is essential to stabilize the activated integrin^38^. CytoD is a well-recognized inhibitor of actin polymerization^39^, which is also likely involved in maintaining the activation state of integrins. We, therefore, revisited the effect of CytoD on Notch activation upon efferocytosis in WT cells. THP-1 cells treated with CytoD failed to establish the activated integrin barrier, which was concordant with compromised Notch exclusion at IgG contact regions (Fig. 4d and Extended Data Fig. 6d). These results suggest that the generation and maintenance of the activated integrin barrier are essential for Notch exclusion, which led us to ask how the Rubcn–VPS34 complex regulates integrin activation.

**Fig. 5:**
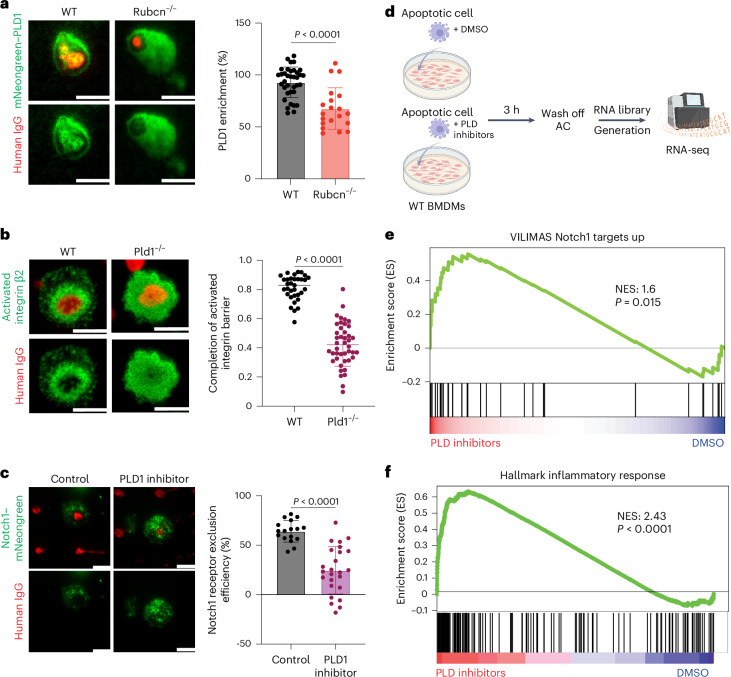
PLD is involved in activated integrin barrier formation and regulation of Notch exclusion. **a**, TIRF imaging of WT (*n* = 36) and *Rubcn*^*−/−*^ (*n* = 20) THP-1 cells expressing mNeongreen–PLD1 cultured on micropatterned human IgG for 10 min. **b**, Representative TIRF images (left) and quantification (right) of active β2 integrin staining in WT (*n* = 33) and *PLD1*^*−/−*^ (*n* = 43) THP-1 cells cultured on arrayed human IgG dots. **c**, TIRF imaging of Notch exclusion. THP-1 cells were pretreated with 15 μg ml^−1^ PLD1 inhibitor VU0359595 for 20 h and seeded on micropatterned human IgG for 10 min. Results of two independent experiments were combined. Control, *n* = 17; PLD inhibitor, *n* = 25. Scale bars, 10 μm. **d**, Schematic of experimental design. WT BMDMs were cultured with apoptotic Jurkat cells in the presence of DMSO or 10 μg ml^−1^ PLD1 inhibitor VU0359595 and 2.5 μg ml^−1^ PLD2 inhibitor VU0364739 hydrochloride for 3 h. ACs were then washed off before RNA extraction for RNA-seq. **e**,**f**, GSEA of upregulated Notch1 target genes (**e**) and Hallmark genes of inflammatory response (**f**) in WT BMDMs treated with DMSO or PLD inhibitors during efferocytosis as in **d**. Data are the mean ± s.d. Statistical analysis was conducted using a Student’s *t*-test (**a**–**c**). Scale bars, 10 μm. GSEA significance was calculated using one-sided permutation testing based on a preranked approach. Illustrations in **d** were created in BioRender; Verbist, K. https://biorender.com/j30t950 (2026). Source data

The VPS34 complex generates phosphatidylinositol 3-phosphate, which is recognized by proteins harboring a PX domain^40^. One such protein is PLD1, which is known to function in the activation of integrins^41^. We speculated that the recruitment of PLD1 might function in the generation of the β2 integrin barrier that forms at sites of IgG recognition to exclude Notch. We therefore expressed mNeonGreen–PLD1 in THP-1 cells and examined its recruitment to IgG dots. We found that enrichment of PLD1 at IgG dots was dependent on Rubcn (Fig. 5a).

We next ablated PLD1 in THP-1 cells and cultured cells on human IgG micropatterned glass slides to assess the generation of the activated β2 integrin barrier (Extended Data Fig. 6e). We observed that PLD1 ablation compromised the efficiency of barrier formation (Fig. 5b). In accordance with this observation, we found that inhibition of PLD1 with an inhibitor (VU0359595)^42^ or PLD1 ablation prevented Notch exclusion at IgG contact regions (Fig. 5c and Extended Data Fig. 6f). Therefore, the Rubcn–VPS34 complex promotes the recruitment of PLD1, leading to the formation of an activated integrin barrier to exclude Notch.

**Extended Data Fig. 7:**
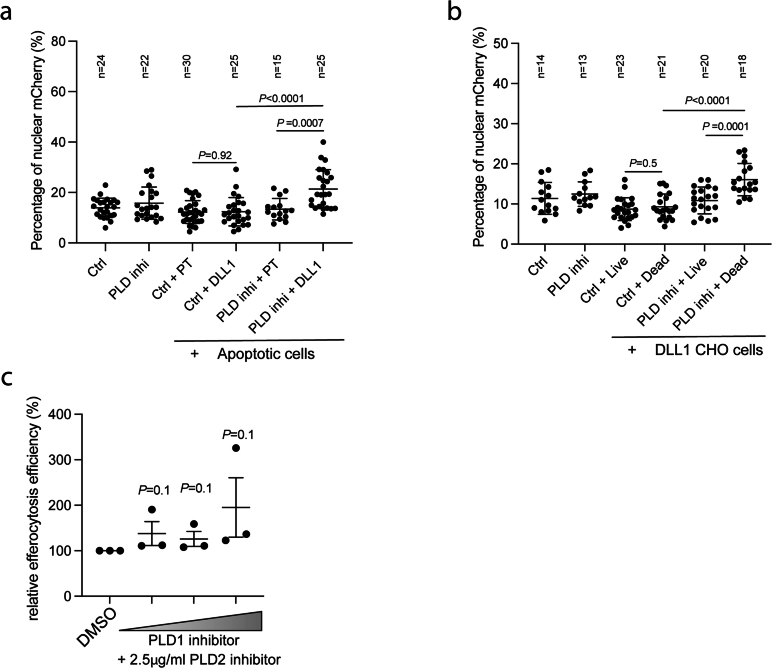
PLD inhibitor treatment increases Notch activation in macrophages cultured with apoptotic but not live DLL1-expressing CHO-K1 cells. (**a** and **b**) Quantification of the percentage of nuclear mCherry in *Hoxb8* BMDM expressing Notch1-mCherry-NLS reporter. *Hoxb8* BMDM were treated with DMSO or 1 μg/ml PLD1 inhibitor, VU035959, combined with 1 μg/ml PLD2 inhibitor, VU0364739, for 3 hours before co-culturing with apoptotic parental and DLL1-expressing CHO-K1 cells (**a**) or co-culturing with live or apoptotic DLL1-expressing CHO-K1 cells (**b**). Activation of Notch1 receptor was quantified by the intensity of mCherry in the nucleus over total for each cell (100 x nuclear/total). Data are mean ± s.d. Each dot represents a single cell. (**c**) WT BMDM were treated with DMSO or 2.5 μg/ml, 5 μg/ml or 10 μg/ml PLD1 inhibitor, VU0359595, for 17 hours then co-cultured with apoptotic Jurkat cells expressing tdTomato in the presence of DMSO or the same concentration of PLD1 inhibitor plus PLD2 inhibitor, VU0364739 (2.5 μg/ml), for 3 hours. Efferocytosis was determined by flow cytometry. Data analysis is from three independent experiments and are presented as mean±s.e.m. Student’s *t* test was used in all analyses. Source data

To further investigate the role of PLD in the regulation of Notch signaling during efferocytosis, we generated BMDMs derived from *ER*-*Hoxb8* immortalized bone marrow (*Hoxb8* BMDMs)^43^ expressing our Notch reporter and cultured *Hoxb8* BMDMs with apoptotic parental CHO-K1 cells or CHO-K1 cells expressing human DLL1 in the presence or absence of PLD inhibitors. Compared to control conditions in which neither parental nor DLL1-expressing apoptotic CHO-K1 cells induced nuclear mCherry translocation, PLD inhibitor treatment significantly increased the percentage of nuclear mCherry, indicative of Notch activation, in *Hoxb8* BMDMs that had engulfed apoptotic DLL1 CHO-K1 cells (Extended Data Fig. 7a). In contrast, culturing *Hoxb8* BMDMs with live CHO-K1 cells expressing DLL1 in the presence or absence of PLD inhibitors did not increase mCherry nuclear localization (Extended Data Fig. 7b). Therefore, Notch ligand on dead but not living cells appears to be necessary for the activation of Notch in *Hoxb8* BMDMs when PLD is inhibited.

We next cultured WT BMDMs with apoptotic Jurkat cells in the presence of DMSO or PLD inhibitors and subjected BMDMs to RNA-seq (Fig. 5d). We found that Notch signaling was activated in BMDMs treated with PLD inhibitors during efferocytosis (Fig. 5e), which could not be attributed to decreased efferocytosis upon treatment with PLD inhibitors (Extended Data Fig. 7c). Inhibition of PLD significantly polarized the efferocytotic macrophages toward proinflammatory responses (Fig. 5f).

### The antitumor effect of Rubcn ablation and PLD inhibition requires RBPJ

**Extended Data Fig. 8:**
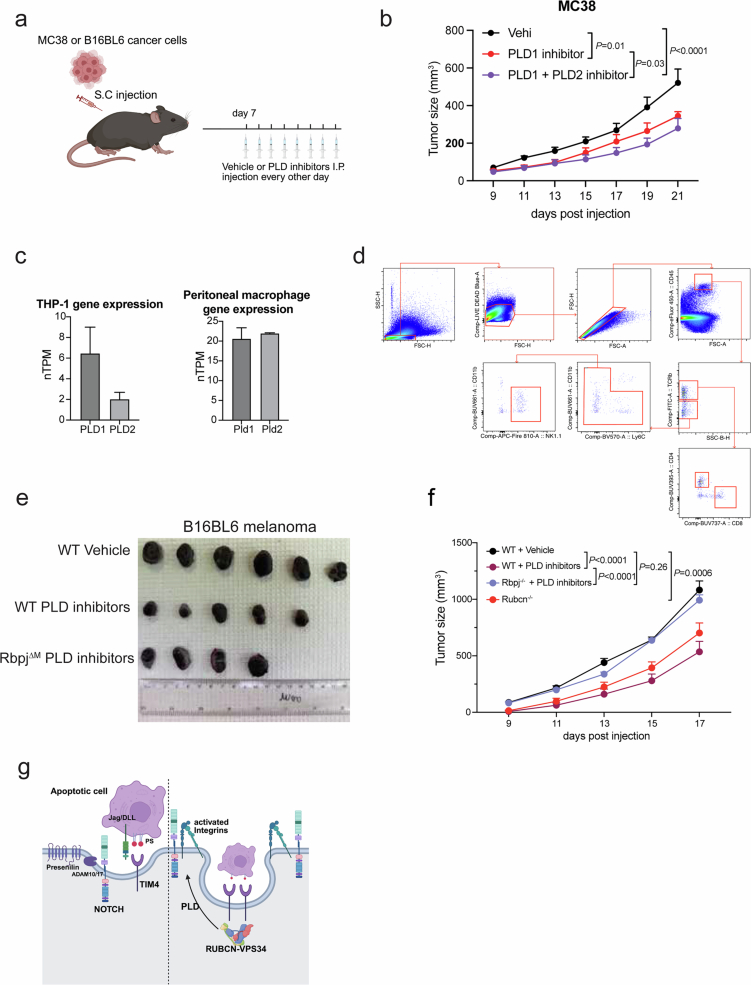
The effect of PLD inhibitors in tumor growth suppression requires Rbpj. (**a**) Schematic of experimental design. MC38 or B16BL6 cancer cells were subcutaneously (s.c.) injected into 6 to 8-week-old WT C57BL/6 mice. Treatment of vehicle or PLD inhibitors through intraperitoneal (i.p.) injection were started on day 7 post tumor injection and performed every other day until the end of the experiment. (**b**) Growth of s.c. engrafted MC38 colon adenocarcinoma tumors in WT mice that were treated with vehicle control (n = 8) or 15 mg/kg (body weight) PLD1 inhibitor (n = 10), VU0359595, or a combination of PLD1 inhibitor and 5 mg/kg (body weight) PLD2 inhibitor (n = 10), VU0364739, every other day via i.p. injection starting on day 7 post tumor cell injection. Data are mean ± s.e.m., 2-way ANOVA. (**c**) *PLD1* and *PLD2* RNA expression in THP-1 cells (normalized TPM data retrieved from Human Protein Atlas) (n = 2) and peritoneal macrophages (n = 4). (**d**) Gating strategy for discrimination of tumor infiltrating NK cells, CD4^+^ T cells and CD8^+^ T cells. (**e**) B16BL6 melanoma tumors collected from mice as in Fig. 6d. (**f**) Growth of s.c. engrafted B16BL6 melanoma in WT (mix of WT or *Rubcn*^*−/+*^; previous studies had shown no difference in tumor growth in *Rubcn*^*−/+*^ vs *Rubcn*^*+/+*^ mice^16^), *Rubcn*^*−/−*^ (*LysM-cre*^*−*^*, Rbpj*^*f/f*^*, Rubcn*^*−/−*^) and *Rbpj*^*ΔM*^ (*LysM-cre*^*+*^*, Rbpj*^*f/f*^*, Rubcn*^*−/+*^ or *Rubcn*^*+/+*^) mice. Six WT mice were treated with vehicle. Six WT mice and 5 *Rbpj*^*ΔM*^ mice were treated with PLD inhibitors. *Rubcn*^*−/−*^ mice received no treatment with PLD inhibitors. All treatments were performed as in (**a**). Data are mean ± s.e.m., 2-way ANOVA. (**g**) A proposed model of Notch suppression during efferocytosis. In WT cells, the engagement of PS and a PS-receptor (such as TIM4) on the dead cell and phagocytes, respectively, induces recruitment of Rubicon-VPS34 complex which activates integrin to form a diffusion barrier and therefore excludes Notch. Illustrations in **a** and **g** created in BioRender; **a**, Verbist, K. https://biorender.com/zm9exfj (2026); **g**, Verbist, K. https://biorender.com/vm6143x (2026). Source data

Our observations suggested that the activity of PLD1 is required to exclude Notch where engulfment occurs (Fig. 5). We, therefore, tested whether PLD inhibitors influence antitumor immunity, as we showed in mice with overactivated Notch signaling in myeloid cells (Fig. 2). Mice subcutaneously engrafted with the murine MC38 colon adenocarcinoma were treated with vehicle or PLD inhibitors every other day starting from day 7 after tumor cell injection (Extended Data Fig. 8a). Tumor growth was significantly delayed by the PLD1 inhibitor treatment (Extended Data Fig. 8b).

**Fig. 6:**
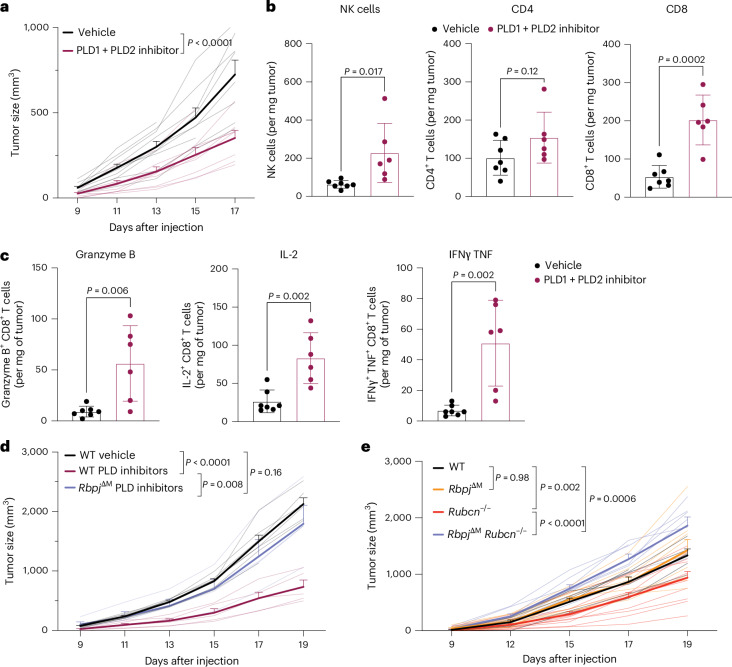
Ablation of Notch signaling abolishes antitumor effect of Rubcn deficiency and PLD inhibition. **a**, Growth of subcutaneously engrafted B16BL6 melanoma in WT mice treated with vehicle control or a combination of 15 mg kg^−1^ (body weight) PLD1 inhibitor VU0359595 and 5 mg kg^−1^ (body weight) PLD2 inhibitor VU0364739 every other day through i.p. injection starting on day 7 after tumor cell injection (*n* = 10 mice per group). **b**, Absolute cell number of NK cells, CD4^+^ T cells and CD8^+^ T cells in tumors shown in **a**. Statistical analysis was conducted using a Student’s *t*-test. Data are the mean ± s.d. **c**, Absolute cell number of cytotoxic CD8^+^ T cells that are granzyme B^+^, IL-2^+^ or IFNγ^+^TNF^+^ in tumors shown in **a**. Statistical analysis was conducted using a Student’s *t*-test. Data are the mean ± s.d. **d**, Tumor growth in WT, *LysM*-*cre*^*−*^ and *LysM*-*cre*^+^;*Rbpj*^f/f^ mice treated with vehicle or a combination of PLD1 and PLD2 inhibitors as in **a**. Vehicle group, *n* = 8 mice; WT inhibitor group, *n* = 6 mice; *Rbpj*^ΔM^ inhibitor group, *n* = 4 mice. **e**, Growth of subcutaneously engrafted B16BL6 melanoma in control (WT or *Rubcn*^*−*/+^, *n* = 6), *Rubcn*^*−*/*−*^ (*LysM*-*cre*^*−*^;*Rbpj*^f/f^;*Rubcn*^*−*/*−*^, *n* = 12), *Rbpj*^ΔM^ (*LysM*-*cre*^+^;*Rbpj*^f/f^, *n* = 9) and *Rbpj*^ΔM^ *Rubcn*^*−*/*−*^ (*LysM*-*cre*^+^;*Rbpj*^f/f^;*Rubcn*^*−*/*−*^, *n* = 8) mice. Statistical analysis for tumor growth was conducted using a two-way ANOVA. Data are the mean ± s.e.m. Source data

Murine macrophages express both *Pld1* and *Pld2*, a *PLD1* homolog reported to stimulate integrin-mediated adhesion^44^, whereas PLD1 is dominant in THP-1 cells, as assessed by mRNA expression (Extended Data Fig. 8c). We found that combined treatment with inhibitors against PLD1 and PLD2 showed better efficacy in inhibiting tumor growth than inhibition of PLD1 alone (Extended Data Fig. 8b). Similarly, PLD inhibitors delayed tumor growth in a B16BL6 melanoma model with increased tumor infiltration of natural killer (NK) cells and CD8^+^ T cells (Fig. 6a,b and Extended Data Fig. 8d). Moreover, CD8^+^ T cells in tumors treated with PLD inhibitors exhibited enhanced expression of effector molecules compared to those in the vehicle control group (Fig. 6c).

We asked whether Notch signaling is required for the antitumor effect of PLD inhibitor treatment. We generated mice lacking Notch-mediated transcription in myeloid cells by crossing *LysM*-*cre*^+^ and *Rbpj*^*flox/flox*^ (*Rbpj*^ΔM^), a key transcription factor for Notch target gene expression^21^. The antitumor effect of PLD inhibitors was abolished in mice in which RBPJ was ablated in macrophages (Fig. 6d and Extended Data Fig. 8e,f). Similarly, we observed that, while tumor growth was suppressed in Rubcn-deficient animals, this antitumor effect was significantly curtailed by RBPJ ablation in macrophages (Fig. 6e). Taken together, Notch signaling is required for the antitumor effects of Rubcn deficiency and PLD inhibitors.

**Extended Data Fig. 9:**
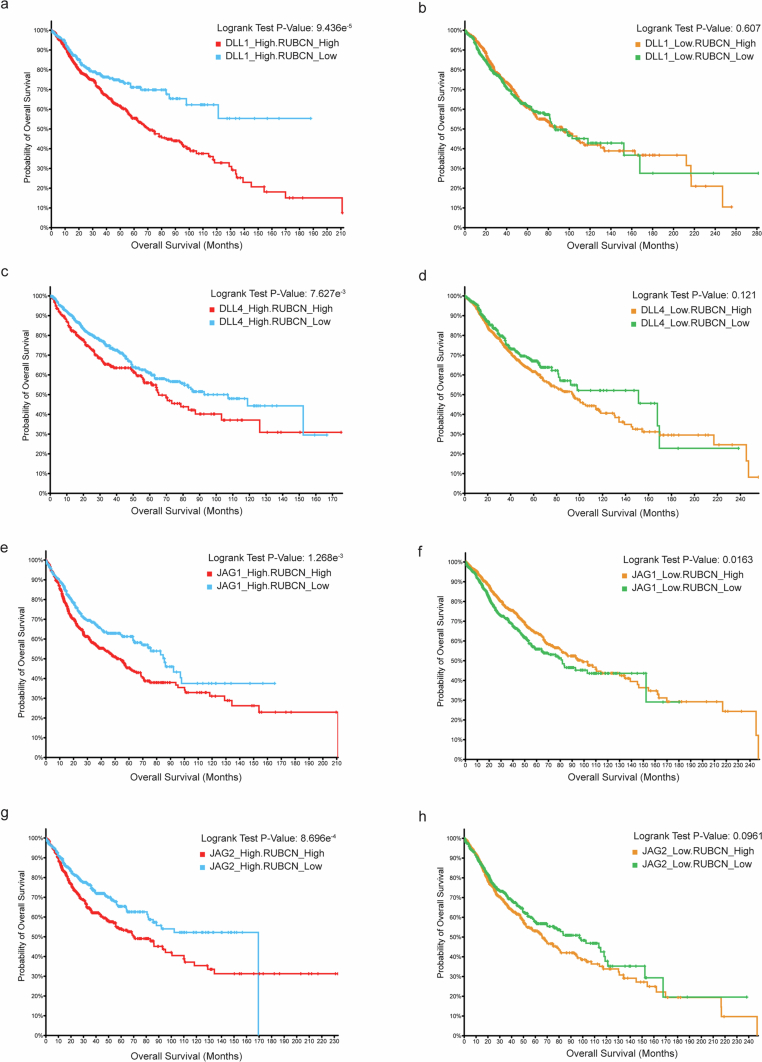
Improved survival of patients in the TCGA database with high Notch ligand and low Rubicon expression. Kaplan-Meier survival curves obtained from all TCGA solid tumor studies (n = 10720). (**a** and **b**) Survival outcomes in patients with low (bottom 25%) or high (top 25%) expression of Rubicon and high (**a**) or low (**b**) expression of *DLL1*. (cand d) Survival outcomes in patients with low (bottom 25%) or high (top 25%) expression of Rubicon and high (**c**) or low (**d**) expression of *DLL4*. (**e** and **f**) Survival outcomes in patients with low (bottom 25%) or high (top 25%) expression of Rubicon and high (**e**) or low (**f**) expression of *JAG1*. (**g** and **h**) Survival outcomes in patients with low (bottom 25%) or high (top 25%) expression of Rubicon and high (**g**) or low (**h**) expression of *JAG2*. Statistical significance evaluated using the Log-rank test.

One possible prediction that emerges from our results is that tumors that express Notch ligands will have an improved prognosis when the TME is deficient in Rubcn as compared to those in which the TME expresses high levels of Rubcn. We analyzed all solid tumors in The Cancer Genome Atlas (TCGA) database, subsetted them into high (top 25%) and low (bottom 25%) expression of the Notch ligands, DLL1, DLL4, JAG1 and JAG4 and compared survival data for those in which Rubcn was high (top 25%) or low (bottom 25%). We found that when any of the Notch ligands was low, Rubcn expression did not affect outcome. However, when any of the Notch ligands was high, those in which Rubcn expression was low consistently displayed improved survival as compared to those in which Rubcn expression was high (Extended Data Fig. 9). We discuss the limitations of this analysis below.

## Discussion

Signaling pathways induced by efferocytosis to facilitate dead cell digestion and metabolism, to stimulate phagocyte proliferation^45^ and to activate the anti-inflammatory program have been well documented^4,6,11,46,47^. We found that efferocytosis also actively suppresses the Notch pathway, which, if activated in myeloid cells, can alter the immunosuppressive TME to promote antitumor immunity.

Previous studies have shown that integrin forms a diffusion barrier upon IgG–FcR engagement and contributes to CD45 exclusion in an ectodomain size-dependent manner^34^. As the ectodomain of Notch is larger than that of CD45, we reasoned that a similar effect would apply to Notch. We found that the presence of IgG suppresses Notch signaling and induces an activated integrin barrier that excludes Notch. Both the integrin barrier and the exclusion of Notch were dependent on the Rubcn–VPS34 complex and PLD. We extended this to dying cells by micropatterning lysed RBCs, thus exposing PS to be recognized by our macrophage cell line.

On the basis of our observations, we propose a model of suppression of Notch signaling during efferocytosis (Extended Data Fig. 8g). Once phagocytes encounter dead cells, the engagement of PS on dead cells with PS receptors on phagocytes triggers an activated integrin barrier at the contact site, which excludes Notch from interacting with its cognate ligand on dead cells. The sustained formation of this active integrin barrier requires the Rubcn–VPS34 complex and its recruitment of PLD.

Although Notch can be activated in *cis* by Notch ligands presented on the same cell^23^, we found that Notch signaling in macrophages and macrophage cell lines in which our proposed pathway was not functional (for example, Rubcn ablation and PLD1 inhibition) required Notch ligand on the apoptotic cell being engulfed (Extended Data Fig. 7a,b). We suggest that this excludes *cis* signaling during efferocytosis. While mechanical force provided by Notch ligand endocytosis has been associated with receptor activation^48^, other force-generating mechanisms are also likely to be involved in Notch activation. For example, Notch receptor activation could be achieved by immobilization of the ligands on a culture plate^49^. Similarly, restricting the lateral diffusion of recombinant DLL4 on lipid membranes leads to increased Notch reporter activity^50^. We speculate that mechanical forces generated during phagocytosis^51^, such as the tethering of PS on dead cells to the macrophage during efferocytosis, may also contribute to Notch receptor activation in Rubcn-deficient macrophages.

The role of Notch signaling in cancer cells is confounding and context dependent as its oncogenic and tumor-suppressive functions have been extensively reported^52–55^. In contrast, the function of Notch signaling in macrophages has been demonstrated to drive proinflammatory polarization^17–20^. In line with this, we observed that overactivation of Notch by expressing an N1ICD C-terminal PEST mutant in macrophages suppressed tumor growth associated with enhanced antitumor immunity, such as increased infiltration and activation of cytotoxic CD8^+^ T cells. Although the model may produce nonphysiological levels of Notch signaling, this result provides insight that activating Notch in macrophages can serve as a potential anticancer therapeutic. In this study, we found that blocking the activity of Rubcn or PLD reduces the efficiency of Notch exclusion, permits Notch signaling upon efferocytosis and drives the proinflammatory polarization of macrophages. This promoted an antitumor effect in mice^16,56^, which we attribute to activation of Notch, as depletion of RBPJ specifically in myeloid cells prevented the anticancer effects of PLD inhibitors or Rubcn ablation. An observed increase in tumor size in *Rubcn*^*−/−*^*Rbpj*^ΔM^ mice as compared to WT may suggest additional effects of global Rubcn ablation in this context.

In our analysis of expression of Notch ligands versus Rubcn in the TCGA database and effects on survival, we do not know if the reported Rubcn expression was in macrophages in the TME, if the differential prognosis was because of improved anticancer immunity or if the response to efferocytosis in the TME was responsible for these effects. Nevertheless, these results are consistent with our findings. It is possible that persons whose tumors express high levels of Notch ligands and low levels of Rubcn may be candidates for immune checkpoint blockade therapy and may be responsive to inhibitors of efferocytosis.

While Notch activation was required for the effects we observed in our studies, we cannot exclude the possibility that additional signals induced upon efferocytosis also have roles in the anticancer effects of Rubcn ablation in vivo. Similar to Rubcn, myeloid ablation of components of the LAP pathway downstream of the Rubcn–VPS34 complex, including ATG5, can promote anticancer effects^16^. We found no role of ATG5, however, in the exclusion of Notch and inhibition of Notch signaling upon efferocytosis, suggesting that it is not LAP per se but the recruitment of Rubcn–VPS34 (functional in LAP) to the contact site that restricts Notch signaling, which also raised the possibility that additional LAP-dependent signals can influence the generation of anticancer immunity.

## Methods

### Mice

Rubcn-deficient mice have been described elsewhere^16^ and NICD mice were obtained from Jackson Laboratories (008159). *RBPJ*^f/f^ mice were gifts from B. Lee (Baylor College of Medicine) and T. Honjo (Kyoto University). *Atg5*^f/f^ and *Fip200*^f/f^ mice were gifts from T. A. Ferguson (Washington University) and J.-L. Guan (University of Michigan), respectively. *Lysm*^*cre*^ mice from Jackson Laboratories (004781) were backcrossed to the C57BL/6J background. Male and female recipient C57BL/6J mice (6–8 weeks old) for tumor studies were purchased from Jackson Laboratories (000664). Age-matched and sex-matched littermates were used as controls. Mice were bred and housed in pathogen-free facilities, in a 12-h light–dark cycle in ventilated cages, with chow and water supply ad libitum at the Animal Resources Center in St. Jude Children’s Research Hospital. Procedures were approved by the Institutional Animal Care and Use Committee at St. Jude and in compliance with all relevant ethical guidelines.

### Cells

Cell lines Yumm1.7 (CRL-3362), HEK293 (CRL-1573), RAW264.7 (TIB-71), Jurkat (TIB-152), CHO-K1 (CCL-61) and L-929 (CCL-1) were purchased from the American Type Culture Collection. HT115 cells (85061104) were purchased from Sigma. MC38 (mouse colon carcinoma) and B16BL6 (mouse melanoma) cells were a gift from Y. Feng (St. Jude Children’s Research Hospital). *ER-Hoxb8* immortalized primary bone marrow cells, used to generate *Hoxb8* BMDMs were a gift from K. Ravichandran (Washintong University at St. Louis). The THP-1 cell line was a gift from P. Thomas (St. Jude Children’s Research Hospital). RAW264.7 Rubcn-knockout and ATG5-knockout cells were generated by CRISPR–Cas9 as previously described^16,57^. HT115, Yumm1.7, HEK293, MC38, RAW264.7 and L-929 cells were cultured in DMEM (Gibco, 11971-025) containing 10% (v/v) heat-inactivated FBS, 2 mM L-glutamine (Gibco, 25030-164), 100 IU per ml penicillin and 100 μg ml^−1^ streptomycin (Corning, 30-001-Cl). THP-1 and Jurkat were cultured in RPMI1 1640 (Gibco, 21870-076), supplemented with 10% heat-inactivated FBS, 2 mM L-glutamine and 100 IU per ml penicillin and streptomycin. *Hoxb8* cells were cultured in RPMI supplemented with 10% FBS, 20 ng ml^−1^ mouse granulocyte-macrophage colony-stimulating factor (StemCell Technologies, 78206) and 1 μM β-estradiol (Sigma, E8875). Cells were maintained in a humidified incubator at 37 °C and 5% CO_2_. Cells were routinely tested for *Mycoplasma* contamination using the MycoAlert *Mycoplasma* detection kit (Lonza, LT07).

THP-1 and Jurkat knockout cell lines were generated by CRISPR–Cas9 using pLentiCRISPR-tagBFP (Addgene, 75160) or pLentiCRISPR–mCherry (Addgene, 75161) with single guide RNAs (sgRNAs) targeting genes of interest. VPS34 knockdown was accomplished through Dox-inducible expression of shRNA targeting VPS34 using pLKO-Tet-ON system (Addgene, 98399). The enrichment of knockout cells was achieved by repetitive fluorescence-activated cell sorting (FACS) using fluorescent proteins and the knockdown cells were selected using puromycin.

*Rubcn* sgRNA sequences: GCTAAGTGACGCTCATGTCA; TATACTGTCTATCCCCGAGC

*Atg5* sgRNA sequences: AAAAAGATCACAAGCAACTC; AAGAAGACATTAGTGAGATA

*Atg14* sgRNA sequences: TTGTTAGGGAGGCTAATCCA; AAAATGGATAACAGATCAGT

*Pld1* sgRNA sequences: GTGAGCCCACAAATAGACGG; TAGGAGGCAAAACGTCAGAG

*Notch2* sgRNA sequences: TTGATGTCCATCTCACAACG; CATTGGTGGATACAGATGCG

*Pik3c3/Vps34* shRNA target sequences: GAGATGTACTTGAACGTAATG; CCACGAGAGATCAGTTAAATA

For preparation of BMDMs, mice of both sexes were killed and bone marrow cells were harvested from the femurs and differentiated in LCM (DMEM containing 20% (v/v) FBS, 30% (v/v) L-929 conditioned medium, 2 mM L-glutamine and 100 IU per ml penicillin and streptomycin). Cells were differentiated for 6 days in 15-cm non-tissue-culture-treated petri dishes with medium supplemented on day 4. L-929 conditioned medium was generated by seeding 200,000 cells per T75 tissue-culture-treated flask (Corning, 430641U). Cells were cultured with complete DMEM for 10 days. Supernatants were collected and filtered (Corning, 431097). Aliquots were frozen at −80 °C and thawed upon each use.

### Plasmids

All plasmids were validated by Plasmidsaurus whole-plasmid sequencing. Mouse Notch1 (amino acids 1–1770) was cloned from Notch1 full-length plasmid (Addgene, 41728) followed by mCherry–NLS or mNeongreen–NLS (synthesized by Integrated DNA Technologies (IDT)) and inserted into pMXs retrovirus vector. Human PLD1 was cloned from plasmid (Addgene, 45268) and tagged with N-terminal mNeongreen. Human FcγRIIA followed by mNeongreen (synthesized by IDT) was cloned into the pLJM1 lentivirus vector. Rubcn constructs were N-terminally tagged with either FLAG or mCherry as described previously^15^. Human *TIMD4* (NM_138379.3) was synthesized by IDT and cloned into lentiCRISPR backbone with dCas9 removed.

### Nuclear fractionation for immunoblotting

To test the Notch receptor proteolytic activation in vitro, BMDMs were seeded at 6.5 × 10^6^ cells per 10-cm dish in LCM one night before experiments. Jurkat or HT115 cells were induced to undergo apoptosis with a combination of 5 μM ABT737 (MedChemExpress, HY-50907) and 5 μM S63845 (MedChemExpress, HY-100741) at 37 °C for 45 min followed by thorough washes with cold Dulbecco’s PBS (DPBS; Thermo, 14190144). To block efferocytosis or proteasome-mediated degradation, BMDMs were pretreated with 1 μM CytoD (Cayman Chemical, 11330) or 20 μM MG132 (MedChemExpress, HY-13259) for 30 min at 37 °C. To inhibit γ-secretase, BMDMs were pretreated with 1 μM DAPT (MCE Cat no. HY-13027) overnight before the experiment. Apoptotic cells were added to BMDMs at a ratio of 5:1 for 3 h at 37 °C with or without the inhibitors as indicated. After 3 h, apoptotic cells were then washed off with cold DMEM. To isolate nuclei for immunoblotting, BMDMs were scraped off and centrifuged. The cell pellet was resuspended in hypotonic buffer (20 mM Tris-HCl pH 7.4, 10 mM KCl, 2 mM MgCl_2_, 2 mM CaCl_2_, 0.5 mM DTT and protease inhibitor cocktail (Roche, 11836170001)) at a concentration of 200 μl per 10^6^ cells and incubated on ice for 3 min. Digitonin (stock 25 mg ml^−1^ in DMSO) was added at a final concentration of 40 μg ml^−1^ and incubated on ice for 3 min, followed by centrifugation at 1,000*g* at 4 °C for 5 min to separate the nuclei (pellet) and cytoplasm (supernatant). Supernatant was transferred to a new Eppendorf tube and centrifuged at top speed at 4 °C for 5 min. The nucleus-containing pellet was washed with 400 μl of isotonic buffer (20 mM Tris-HCl pH 7.4, 150 mM KCl, 2 mM MgCl_2_, 2 mM CaCl_2_, 0.5 mM DTT and protease inhibitor cocktail) containing 0.25% (v/v) CHAPS per 10^6^ cells and incubated on ice for 10 min. The nuclei were centrifuged at 1,000*g* at 4 °C for 3 min. The cytoplasmic fraction was transferred to a new tube with 4× loading buffer to dilute to 1×. The nucleus pellet was lysed with 1× loading buffer. For Extended Data Fig. 2b,f, nuclei were isolated with the Nuc EZ Prep kit (Sigma, NUC101-1KT). Next, 1 ml of lysis buffer was used in the initial lysis step, after which the commercial protocol was followed. All samples were heated at 94 °C for 10 min before western immunoblotting. Nuclear fractionation experiments were repeated at least twice in the same setting or different settings to confirm reproducibility.

### Immunoprecipitation

To coimmunoprecipitate the Rubcn–VPS34 complex, cells were washed with 1× cold PBS and then lysed with lysis buffer (50 mM Tris-HCl pH 7.4, 150 mM NaCl, 5 mM EDTA and 1% Igepal-CA630 (v/v; Sigma, I8896), supplemented with protease inhibitor) for 20 min on ice. Cell lysates were centrifuged at top speed for 10 min at 4 °C, supernatants were collected and protein concentration was quantified (BCA assay; Thermo, 23225). Cell lysates were diluted to 0.2% Igepal-CA630 using the same buffer without detergent. Diluted lysates were subjected to immunoprecipitation with ChromoTek RFP-Trap agarose beads (25 μl per sample; Proteintech, rta) or binding control agarose beads (25 μl per sample; Proteintech, bab) for 4 h at 4 °C with rotation. After extensive washes with 0.2% Igepal-CA630 lysis buffer, protein complexes were solubilized in loading buffer and analyzed by standard immunoblotting.

### In vitro Notch activation

A Notch receptor activation reporter was generated as described above and used to make RAW264.7 reporter cell lines. To make beads conjugated to DLL1 and DLL1 + IgG or PS, 5 μg of biotinylated DLL1 (Acro Biosystems, DL1-H82E5-25ug) or 5 μg of biotinylated DLL1 plus 5 μg of biotinylated mouse IgG1 Fc (Acro Biosystems, IG1-M8211-25ug) or 5 μg of biotinylated PS (Echelon Biosciences, L-31B16; dissolved in ethanol) were used per 100 μl of Streptavidin-coated polystyrene particles (Spherotech, SVP-50-5). For culture with PS + DLL1 beads and DLL1-only beads (Fig. 1h), 10 μl of Streptavidin Fluoresbrite YG microspheres (Polysciences, 24157-1) were used to conjugate 5 μg of biotinylated DLL1 and then mixed with PS + DLL1 beads as described above. The conjugation followed the commercial protocol except that the biotinylated PS was first incubated with Streptavidin beads in 10 μl of ethanol for 15 min at room temperature followed by conjugation with bio-DLL1 in 100 μl of PBS for 30 min at 4 °C. Then, 30 μl of beads were applied to 150,000 cells seeded on 18-mm glass slides (Fisher Scientific, 50-192-9532) the previous night. After 3 h of incubation at 37 °C, cells were fixed with 4% (v/v) formaldehyde (Thermo, 28908) for 10 min at room temperature and then washed with DPBS. The slides were mounted with antifade glass mounting medium supplemented with NucBlue (Thermo, P36985) and imaged using a Marianas spinning disk confocal microscope (Intelligent Imaging Innovations) comprising an inverted AxioObserver Z.1 (Zeiss), CSU-W with SoRA (Yokogawa), Prime95B scientific complementary metal–oxide–semiconductor (sCMOS) camera (Photometrics) and solid-state laser illumination with wavelengths as appropriate. Cells were segmented on the basis of nuclear staining using Imaris software.

To test Notch activation during efferocytosis in vitro, *Hoxb8* cells were seeded on 18-mm glass slides and differentiated with LCM for 6 days to generate *Hoxb8* BMDMs^58^. Live CHO-K1 cells expressing DLL1 were seeded one night before the experiment under the indicated conditions. Otherwise, parental or CHO-K1 cells expressing DLL1 were induced to undergo apoptosis with 5 μM ABT737 and 10 μM S63458 and were added to differentiated *Hoxb8* BMDMs that had been treated with or without 1 μg ml^−1^ PLD1 inhibitor VU0359595 and 1 μg ml^−1^ PLD2 inhibitor VU0364739 hydrochloride for 3 h. *Hoxb8* BMDMs were incubated with apoptotic cells at 37 °C for 3 h before washing off dead cells and then subjected to fixation and imaging as described above.

### Micropatterning

Micropatterning of IgG was performed as described^34^. Briefly, a polydimethylsiloxane stamp (3-μm-diameter pillar and 20-μm spacing; Research Micro Stamps) was incubated with human IgG (Sigma, I2511) at room temperature for 5 min. Conformal contact was made for 10 s with the glass coverslip to transfer IgG. Then, the coverslip was assembled with eight-well chambers (Ibidi, 80808) and blocked with blocking buffer (PBS, 2% (w/v) BSA and 0.05% (v/v) Tween-20) for 30 min at room temperature. To visualize the IgG, coverslips were stained with Alexa Fluor 647-conjugated donkey anti-human IgG secondary antibody (Jackson ImmunoResearch, 709605149) for 15 min.

For micropatterning of membranes with PS, 15 μl of goat RBCs (Innovative Research, IGTRBC100P15ML) was added in 500 μl of water with 2 μl of Vybrant DiD cell-labeling solution (Thermo, V22887). Then, 15 μl of the diluted goat RBCs were quickly smeared onto a glass cover slide. The slide was dried at room temperature for 5 min and overlaid with Ibidi sticky well (Ibidi, 80808). Slides were washed with complete RPMI medium three times. Activated THP-1 cells expressing Notch1–mNeongreen were then seeded on top of the RBC membranes for 10 min at 37 °C. Cells were imaged by TIRF as described above. To visualize PS, Alexa Fluor-conjugated annexin V (Biolegend, 640911) was used in annexin V binding buffer.

### TIRF microscopy

TIRF microscopy was performed using a Marianas multimodal system (Intelligent Imaging Innovations) comprising an inverted AxioObserver Z.1 inverted microscope equipped with a TIRF slider (Zeiss), ×100 (numerical aperture (NA): 1.45) oil objective, 488-nm and 647-nm solid-state laser lines and emission filters as appropriate and a Prime95B sCMOS camera (Photometrics). Images were acquired and analyzed using Slidebook software (version 2024.1) and Imaris. Additional TIRF microscopy was performed using an inverted Ti2 microscope equipped with a TIRF illumination arm (Nikon), ×100 (NA: 1.45) oil objective, 488-nm and 647-nm laser lines and emission filters as appropriate and an iXon DU897 electron-multiplying charge-coupled device camera (Andor). Images were acquired using NIS Elements software (version 5.21.03). Because of irregularities in the glass slides, slight adjustments to attain TIRF illumination sometimes varied from image to image without affecting data acquisition.

### Immunofluorescence

THP-1 cells were treated with 20 ng ml^−1^ phorbol 12-myristate 13-acetate (PMA; InvivoGen, tlrl-pma) for 16 h at 37 °C. Cells were then washed by replacing fresh complete RPMI medium and cultured for another 24 h at 37 °C. To image activated integrin β2, activated THP-1 cells were seeded on micropatterned slides and incubated for 30 min at 37 °C. Cells were fixed with 4% (v/v) formaldehyde for 10 min at room temperature and washed with PBS, followed by permeabilization with buffer containing PBS and 0.1% (v/v) Triton X-100 for 5 min at room temperature. Cells were then treated with blocking buffer described above for 30 min at room temperature. Anti-human activated integrin β2 Dylight 488 antibody (Leinco Technologies, clone KIM127) was diluted 1:100 in blocking buffer and applied to cells overnight at 4 °C. Cells were then washed three times with wash buffer (PBS and 0.01% (v/v) Tween-20) and immediately imaged by TIRF as described above.

### Analysis of integrin barrier formation

Individual cells from TIRF images were analyzed by sampling a line that sections the micropatterned dot and the integrin barrier. Fluorescence intensities on the selected channels in this region of interest were then determined with Slidebook software. The completion of an activated integrin barrier was calculated as 1 − *X*/*Y*, where *X* is the lowest intensity of integrin (in the center of dot) and *Y* is the highest intensity of integrin (enriched around the dot).

### Analyzing exclusion

Activated THP-1 cells expressing a fluorescent protein-tagged reporter as indicated in figures (for example, Notch1–mNeongreen–NLS and FcγRIIA–mNeongreen, obtained as described above) were seeded on fresh human micropatterned slides and incubated for 10 min at 37 °C. The exclusion of reporter at plasma membrane was imaged by TIRF as described above. To analyze the exclusion, the mean fluorescence intensity (MFI) of channels of interest over the IgG (channel Cy5) was assessed as *X* and the MFI of the interested channel over the whole cell or background was assessed as *Y* and *B*, respectively. The percentage of exclusion from the micropatterned dot was calculated as (1 − (*X* − *B*/*Y* − *B*)) × 100.

For FcR blocking, 0.3 × 10^6^ activated THP-1 cells were first incubated with 18 μl of human FcR Blocker (BD Biosciences, 564219) and 15.2 μg of anti-human CD32 antibody (BioXell, BE0224) at 37 °C for 30 min and then seeded onto human IgG micropatterned slides for 10 min. For CytoD inhibition treatment, activated THP-1 cells were pretreated with 1 μM CytoD for 15 min and then cultured on human micropatterned slides for another 15 min. For PLD1 inhibitor treatment, activated THP-1 cells were pretreated with 15 μg ml^−1^ PLD1 inhibitor VU0359595 (Cayman Chemical, 10955) for 18 h at 37 °C followed by TIRF imaging as described above.

### In vivo tumor studies

For in vivo tumor generation, 0.25 × 10^6^ of the indicated tumor cells were injected subcutaneously into mice (aged 6–10 weeks). Sample sizes were chosen on the basis of preliminary data. Tumor-bearing mice were randomly assigned to the groups. Investigators were not blinded to the tumor analysis. Animals were killed when tumor measurements exceeded ~2,000 mm^3^ as approved by the Institutional Animal Care and Use Committee. Tumors were measured with calipers three times a week and tumor volumes were calculated using the formula (width^2^ × length)/2. Mice were removed if there was no tumor growth during the experiment. For PLD1 and PLD2 inhibitor treatment, vehicle (40% (v/v) PEG-300 and 60% (v/v) DPBS) or 15 mg kg^−1^ (body weight) PLD1 inhibitor VU0359595 (AOBIOUS, AOB1035) ± 5 mg kg^−1^ (body weight) PLD2 inhibitor VU0364739 hydrochloride (Tocris, 417110) were administered through i.p. injection every other day beginning on day 7 after tumor injection. For the examination of tumor-infiltrating lymphocytes, tumors were excised, minced and digested with 0.5 mg ml^−1^ collagenase IV (Roche) + 200 IU per ml DNase I (Sigma) for 40 min at 37 °C and then passed through 70-μm filters to remove undigested tumor tissues.

### Flow cytometry

Antibodies are listed in the Supplementary Information. For surface markers, the cells were stained for 30 min on ice in PBS containing 2% fetal calf serum and 0.1% sodium azide (Sigma-Aldrich, S2002). To examine intracellular cytokines and transcription factors, isolated cells were stimulated with a cell activation cocktail (TOCRIS, 5476) in the presence of monensin (BD Biosciences, 554724) for 4 h at 37 °C and then stained using a fixation and permeabilization kit (Ebiosciences, 00-5523-00) per the manufacturer’s instructions. Fixable viability dye (Thermo Fisher) was used to exclude dead cells. Samples were acquired on a Cytek Aurora (SpectroFlo) flow cytometer and analyzed using FlowJo software.

### RT–qPCR

To assess the expression of genes during efferocytosis in vitro, 2.5 × 10^6^ WT or *Rubcn*^*−/−*^ BMDM cells were seeded per 6-cm dish and cultured one night before the experiment. To induce efferocytosis, apoptotic Jurkat cells were incubated with BMDMs at 37 °C for 3 h before being washed off with DMEM, followed by total RNA extraction with TRIzol. To assess the expression of Notch target genes in vivo, apoptotic parental or DLL1-expressing CHO-K1 cells were labeled with live/dead red (Thermo, L34972) and injected i.p. into WT and *Rubcn*^*−/−*^ mice (three mice for each condition). Then, 3 h after injection, peritoneal macrophages were collected and subjected to sorting of DAPI^*−*^, CD45–APCfire750^+^, Ly-6g/Ly-6c–PEcy7^*−*^, CD11b–BV785^+^, F4/80–APC^+^ and live/dead red^+^ efferocytotic macrohphages into TRIzol LS followed by RNA extraction. Complementary DNA (cDNA) was generated with the high-capacity cDNA RT kit (Thermo, 4368814) and RT–qPCR was performed. PCR primers are listed below.

*Tlr9*: forward, ATGGTTCTCCGTCGAAGGACT; reverse, GAGGCTTCAGCTCACAGGG

*Lta*: forward, CCACCTCTTGAGGGTGCTTG; reverse, CATGTCGGAGAAAGGCACGAT

*Nlrp12*: forward, AAGACCGCAATGCACGATTAG; reverse, TGGAGCGTTCCCACTCTACA

*Ffar2*: forward, CTTGATCCTCACGGCCTACAT; reverse, CCAGGGTCAGATTAAGCAGGAG

*Dtx1*: forward, ATCAGTTCCGGCAAGACACAG; reverse, CGATGAGAGGTCGAGCCAC

*Hes1*: forward, TCAGCGAGTGCATGAACGAG; reverse, CATGGCGTTGATCTGGGTCA

*Actb*: forward, TGTTACCAACTGGGACGACA; reverse, GGGGTGTTGAAGGTCTCAAA

*Rpl6*:forward, AAGCCCAAGAAGGCGAAGC; reverse, GCAGCCGAGTATTTCCTTTTGTA

### SLAM-seq

SLAM-seq was performed using a commercially available protocol (Lexogen, 061.24 and 229.24). Briefly, WT or *Rubcn*^*−/−*^ BMDMs were seeded at 60–80% confluency one night before the experiment. BMDMs were fed with apoptotic Jurkat cells at a ratio of 5:1 dead cells to macrophages. To label nascent RNA, BMDMs were treated with 100 μM 4-thiouridine (s^4^U) and incubated at 37 °C for 2 h. Dead cells were then washed off with warm complete medium. Cells were then cultured for another 2 h in the presence of 100 μM s^4^U at 37 °C. Total RNA was extracted with TRIzol LS (Thermo, 10296010) for library construction.

### RNA-seq

For efferocytotic BMDM samples, BMDMs from WT or *Lysm*^*cre*^*ATG5*^fl/fl^ mice were seeded at a concentration of 6 × 10^6^ per 10-cm dish in LCM one night before experiments. Jurkat cells were treated with 5 μM ABT737 and 5 μM S63845 for 1.5 h at 37 °C and thoroughly washed with complete DMEM before incubation with BMDMs for 2 h at 37 °C. Apoptotic cells were then washed off with complete DMEM. BMDMs were then cultured in complete DMEM for another 2 h at 37 °C and then lysed with TRIzol LS (Thermo, 10296010). For peritoneal macrophages, DPBS or 4 × 10^6^ apoptotic Jurkat cells expressing tdT were injected i.p. into WT and *Rubcn*^*−/−*^ mice. Then, 3 h after injection, peritoneal macrophages were collected through i.p introduction and collection of 5 ml of cold DPBS plus 3% (v/v) FBS. Control (tdT^−^) or efferocytotic macrophages (tdT^+^) underwent FACS on the basis of the markers DAPI^*−*^, CD45^+^ (clone 30-F11), CD11b^+^ (clone M1/70) and F4/80^+^ (clone BM8) directly into TRIzol LS. For TAMs, tumors were collected and minced into small pieces using razor blades. The tumors were digested in dissociation buffer containing 1 mg ml^−1^ collagenase IV (Worthington Biochemicals, LS004188) and 0.5 mg ml^−1^ of DNase I (Sigma, DN25-1G) at 37 °C for 30 min on a three-dimensional orbital mixer. The cell suspensions were then passed through 70-μm filters to remove undigested tissues. Macrophages in tumors underwent FACS on the basis of the markers DAPI^*−*^, CD45^+^ (clone 30-F11), CD11b^+^ (clone M1/70), F4/80^+^ (clone BM8), Ly-6c^*−*^ (clone HK1.4) and Ly-6g^*−*^ (clone 1A8) into TRIzol LS. Total RNA was purified by following the commercially available TRIzol RNA extraction protocol and quantified using the Agilent 4200 TapeStation. Libraries were prepared using the KAPA RNA HyperPrep Kit with RiboErase (Fisher Scientific, 50-196-5257). Libraries were quantified and size distribution was determined using the Agilent 4200 TapeStation before paired-end sequencing was performed.

For RNA-seq with CytoD treatment, WT BMDMs were seeded at 1 × 10^6^ cells per well in six-well plate one night before experiment. On the second day, apoptotic Jurkat (induced by 5 μM ABT737 and 5 μM S63845 for 1 h) were cultured with DMSO-treated or 1 μM CytoD-treated WT BMDMs (pretreated with 1 μM CytoD for 1 h before adding apoptotic cells) for 3 h. Then, the dead cells were washed off with complete DMEM and the BMDMs were lysed with TRIzol LS for RNA extraction.

For RNA-seq with PLD inhibitors, WT BMDMs were seeded at 1 × 10^6^ cells per well in a six-well plate one night before the experiment and pretreated with DMSO or 10 μg ml^−1^ VU0359595 for 16 h. On the second day, apoptotic Jurkat cells were cultured with BMDMs in the presence of DMSO or 10 μg ml^−1^ VU0359595 and 2.5 μg ml^−1^ VU0364739 hydrochloride for 3 h after which the apoptotic cells and drugs were washed off. BMDMs were then subjected to RNA extraction with TRIzol LS.

### RNA-seq analysis

Paired-end 100-cycle sequencing was performed on Illumina NovaSeq sequencers per the manufacturer’s directions (Illumina). Reads were first trimmed for adaptors using fastp (version 0.20.0; paired-end mode, parameters: ‘--detect_adapter_for_pe --trim_poly_x --cut_by_quality5–cut_by_quality3 --cut_mean_quality 15 --length_required 20 --low_complexity_filter --complexity_threshold 30’)^59^. Then, alignment to the hybrid reference genome hg38(GRCh38.p13) and mm10(GRCm38.p6) was performed using STAR (version 2.7.1a; parameters: ‘--outFilterMultimapNmax 20 --alignSJoverhangMin 8 --alignSJstitchMismatchNmax 5 -1 5 5 --alignSJDBoverhangMin 10 --outFilterMismatchNmax 999 --outFilterMismatchNoverReadLmax 0.04 --alignIntronMin 20 --alignIntronMax 100000 --alignMatesGapMax 100000 --outSAMmapqUnique 60 --outSAMmultNmax 1 --outSAMstrandField intronMotif --outSAMattributes NH HI AS nM NM MD --outSAMunmapped Within --chimSegmentMin 12 --chimJunctionOverhangMin 12 --chimSegmentReadGapMax 3 --chimMultimapNmax 10 --chimMultimapScoreRange 10 --chimNonchimScoreDropMin 10 --chimOutJunctionFormat 1 --quantMode TranscriptomeSAM GeneCounts --twopassMode Basic --peOverlapNbasesMin 12 --peOverlapMMp 0.1’)^60^. Gene-level quantification was determined by HTSeq (version 0.11.2, union mode)^61^ based on GENCODE v31 and vM22 gene annotation^62^. After confirming that the samples with human dead cells had significantly more human reads, murine gene counts were extracted for downstream analysis. After normalization by trimmed mean of *M*-value normalization, we applied the empirical Bayes statistics test to detect significant differentially expressed genes after linear fitting from the Voom package (R 3.23, edgeR 3.12.1, limma 3.26.9)^63^. For interaction differentially expressed genes, the model.matrix function was used to generate a design matrix (for example, ‘mm <- model.matrix(~If_Dead_Cell*If_KO)’). GSEA (version 4.0.3)^64^ prerank mode was run with MSigDB (version 7.4)^65^ gene sets against the gene rank by log_2_ fold change.

### SLAM-seq data analysis

Single-end reads of 100-bp reads obtained from all samples were adaptor-trimmed using cutadapt (version 1.9; default parameters: ‘-m 25 -O 6’)^66^ and aligned to hybrid reference genome hg38 (GRCh38.p13) and mm10 (GRCm38.p6) by SLAMDUNK (version 0.4.0; parameters: ‘-5 12 -n 100 -c 2 -mv 0.2 -m -rl 100’)^67^. The 3′UTR BED file for Gencode v31 and vM22 (for each gene, 3′UTR regions from different isoforms were merged to avoid duplicated counting)^62^ were used for counting reads and collapsed into gene-based counts (SLAMDUNK collapse function). All samples achieved >100 million reads (median: 120 million) and >98.4% mapping rates. Quality control results from SLAMDUNK (alleyoop function, all default parameters except ‘-mq 2’ for rates subfunction) were extensively reviewed. Quality control results were comparable to published data from a SLAM-seq paper^68^.

We first filtered genes that required at least ‘readCount’ = 10, ‘tcReadCount’ = 2 and counts per million > 1 from the SLAMDUNK results. Then, for each contrast, we performed two differential expression analyses using two normalization methods. For the nascent RNA analysis, we normalized by tcReadCount using the trimmed mean of *M*-values normalization method. For the normalized nascent RNA analyses, we normalized to total readCount. Lastly, for both analyses, we applied empirical bayes statistics test to tcReadCount after linear fitting from voom packge (R 3.23, edgeR 3.12.1, limma 3.26.9)^63^. For interaction differentially expressed genes, the model.matrix function was used to generate a design matrix (for example, ‘mm <- model.matrix(~If_Dead_Cell*If_KO)’). GSEA (version 4.0.3)^64^ prerank mode was run with MSigDB (version7.4)^65^ gene sets against the gene rank by log_2_ fold change.

### TCGA database analysis

RSEM expression values (batch-normalized from Illumina HiSeq RNASeqV2) were retrieved from TCGA PanCancer Atlas Studies (December 20, 2024) at cBioPortal (https://www.cbioportal.org/). After removing non-solid tumor samples (acute myeloid leukemia, chronic myeloid leukemia, diffuse large B cell lymphoma, miscellaneous, formalin-fixed paraffin-embedded and contro), we grouped samples by quartile (top or bottom 25%) expression of Notch ligands and *Rubcn* genes. We then plotted Kaplan–Meier survival curves using cBioPortal by assigned groups.

### Statistics

Statistical significance was calculated using either an unpaired two-tailed Student’s *t*-test or two-way analysis of variance (ANOVA) through GraphPad Prism software, as specified in figure legends. Data distribution was assumed to be normal but this was not formally tested. No statistical methods were used to predetermine sample sizes. Data collection and analysis were not performed blind to the conditions of the experiments.

### Reporting summary

Further information on research design is available in the Nature Portfolio Reporting Summary linked to this article.

## Online content

Any methods, additional references, Nature Portfolio reporting summaries, source data, extended data, supplementary information, acknowledgements, peer review information; details of author contributions and competing interests; and statements of data and code availability are available at 10.1038/s41590-026-02452-3.

## Supplementary information

Supplementary Information Key resource table.

Reporting Summary

## Source data

Source Data Fig. 1 Statistical source data.

Source Data Fig. 2 Statistical source data.

Source Data Fig. 3 Statistical source data.

Source Data Fig. 4 Statistical source data.

Source Data Fig. 5 Statistical source data.

Source Data Fig. 6 Statistical source data.

Source Data Extended Data Fig. 1 Statistical source data.

Source Data Extended Data Fig. 2 Statistical source data.

Source Data Extended Data Fig. 3 Statistical source data.

Source Data Extended Data Fig. 4 Statistical source data.

Source Data Extended Data Fig. 5 Statistical source data.

Source Data Extended Data Fig. 6 Statistical source data.

Source Data Extended Data Fig. 7 Statistical source data.

Source Data Extended Data Fig. 8 Statistical source data.

Source Data Uncropped and unprocessed blots.

## Data Availability

Sequence data were deposited to the Gene Expression Omnibus under accession code GSE283550. Source data are provided with this paper.
