# Supplementary material for: Exclusion of Notch from the contact site during efferocytosis restricts anticancer immunity
Source: Nat Immunol. 2026 Mar 3;27(4):750–61. doi: 10.1038/s41590-026-02452-3 (PMC13043306; doi:10.1038/s41590-026-02452-3)
Supplement: Supplementary file 1 — Key resource table. [file 41590_2026_2452_MOESM1_ESM.pdf]

nature immunology

Supplementary information https://doi.org/10.1038/s41590-026-02452-3

Exclusion of Notch from the contact site during efferocytosis restricts anticancer immunity

In the format provided by the authors and unedited

Supplementary Table 1

REAGENT or RESOURCE	SOURCE	IDENTIFIER
Experimental models: Organisms/strains		
C57BL/6	The Jackson Laboratory	Stock #000664
Rosa$^{N1\text{-}IC}$	The Jackson Laboratory	Stock #008159
B6: Lysm^{cre}	The Jackson Laboratory	Stock #004781
Atg5$^{f/f}$	Thomas A. Ferguson Washington University	N/A
RBPJ$^{f/f}$	Brendan Lee Baylor College Tasuku Honjo Kyoto University	N/A
Rubcn$^{-/-}$	(Cunha et al., 2018)	N/A
Antibodies		
anti-Notch2	Cell Signaling Technology	Cat#5732S
Anti-cleaved Notch1 (Val1744)	Cell Signaling Technology	Cat#4147S
anti-TBP	Cell Signaling Technology	Cat#44059S
anti-Apaf-1	Cell Signaling Technology	Cat#8969S
anti-Rubcn	Cell Signaling Technology	Cat#8465
anti-Atg5	Cell Signaling Technology	Cat#12994
anti-beta actin HRP conjugated	Santa Cruz	Cat#sc-47778
anti-Jagged1	Cell Signaling Technology	Cat#70109T
anti-Lamin B1	Cell Signaling Technology	Cat#12586S
anti-PIK3R4/VPS15	Cell Signaling Technology	Cat#14580
anti-PIK3C3/VPS34 (Clone D9A5)	Cell Signaling Technology	Cat#4263
anti-Beclin1	Cell Signaling Technology	Cat#3738
anti-Uvrag	Cell Signaling Technology	Cat#13115S
anti-Atg14	Cell Signaling Technology	Cat#96752
anti-Pld1	Cell Signaling Technology	Cat#3832S
Anti-PDI	Cell Signaling Technology	Cat#3501
Anti-RCAS1	Cell Signaling Technology	Cat#12290
Anti-Dll1	Abcam	Cat#ab85346
anti-human CD18/CR3 Dylight 488 (clone KIM127)	Leinco Technologies	Cat#C565
Human IgG	Sigma	Cat#12511
anti-human IgG Alexa Fluor647	Jackson ImmunoResearch	Cat#709-605-149
PerCP/Cy5.5 anti-mouse TCRβ	BioLegend	Cat#109228
Alexa Fluor647 anti-human/mouse GranzymeB	BioLegend	Cat#515405
BV605 anti-mouse NK1.1	BioLegend	Cat#108739

St. Jude - Confidential

BV421 anti-mouse CD45	BioLegend	Cat#103133
BUV737 anti-mouse CD4	BD Biosciences	Cat#612844
BUV805 anti-mouse CD8α	BD Biosciences	Cat#612898
PE anti-mouse IFNγ	BD Biosciences	Cat#505808
PE-Cy7 anti-mouse TNFα	BD Biosciences	Cat#561041
BV785 anti-mouse CD11b	BioLegend	Cat#101243
FITC anti-mouse MHC-II	eBioscience	Cat#11-5321-82
PE-CF594 anti-Mouse CD11c	BD Biosciences	Cat#562454
APC-Fire750 anti-mouse CD45	BioLegend	Cat#147714
eF450 anti-mouse CD45.2	eBioscience	Cat#48-0454-82
FITC anti-mouse TCRβ	BD Biosciences	Cat#553170
BUV737 anti-Mouse CD8	BD Biosciences	Cat#741811
BUV395 anti-Mouse CD4	BD Biosciences	Cat#563790
PerCP-eF710 anti-mouse MHC-II	eBioscience	Cat#46-5321-82
PE-Cy7 anti-mouse CD11c	eBioscience	Cat#25-0114-82
BUV661 anti-mouse CD11b	BD Biosciences	Cat#612977
Spark YG 593 anti-mouse F4/80	BioLegend	Cat#157311
BV570 anti-mouse Ly6c	BioLegend	Cat#128029
APC/Fire 810 anti-mouse NK-1.1	BioLegend	Cat#156519
APC anti-mouse CD45	BD Bioscience	Cat#559864
Alexa Fluor 532 anti-mouse TCRβ	eBioscience	Cat#58-5961-82
PerCP anti-mouse CD8a	BioLegend	Cat#100732
PE-Cy5 anti-mouse Granzyme-B	BioLegend	Cat#372226
PerCP-Cy 5.5 anti-mouse IL-2	eBioscience	Cat#45-7021-82
APC/Cy7 anti-mouse TNFα	BD Bioscience	Cat#506344
APC anti-mouse F4/80	Invitrogen	Cat#17-4801-82
PEcy7 anti-mouse Gr1	Biolegend	Cat#108416
Alexa Fluor647 Annexin V	Biolegend	Cat#640911

Bacterial strains		
NEB® Stable Competent E. coli (High Efficiency)	NEB	Cat#C3040I
Stellar™ Competent Cells	TAKARA	Cat#636766

Chemicals, peptides, and recombinant proteins		
Collegenase IV	Worthington	Cat#LS004188
DNase I	Sigma	Cat#DN25-1G
GlycoBlue™ Coprecipitant	Thermo Fisher Scientific	Cat#AM9515
Ionomycin	Sigma	Cat#I0634
Monensin	BD Biosciences	Cat#554724
Collagenase/Hyaluronidase	StemCell Technologies	Cat#07912
ABT-737	Medchemexpress	Cat#HY-50907
S63845	Medchemexpress	Cat#HY-100741
Percoll	Cytiva	Cat#17089101
Phorbol 12-myristate 13-acetate (PMA)	InvivoGen	Cat#tlrl-pma
Streptavidin Coated Polystyrene Particles	Spherotech	Cat#SVP-50-5
Biotinylated DLL1	Acro Biosystems	Cat#DL1-H82E5-25ug
Biotinylated mouse IgG1 Fc	Acro Biosystems	Cat#IG1-M8211-25ug

Biotinylated Phosphatidylserine	Echelon Biosciences	Cat#L-31B16
ChromoTek RFP-Trap® agarose beads	Proteintech	Cat#rta
Cytochalasin D	Cayman Chemical	Cat#11330
MG132	Medchemexpress	Cat#HY-13259
protease inhibitor cocktail	Roche	Cat#11836170001
Digitonin	Sigma	Cat#D141
Igepal-CA630	Sigma	Cat#I8896
Doxycycline	Fisher Scientific	Cat#16061652
Blasticidin	InvivoGen	Cat#ant-bl-1
Puromycin	Thermo	Cat#A1113803
Lipofectamine 2000	Thermo	Cat#11668019
Prolong Antifade Glass Mounting medium with NucBlue	Thermo	Cat#P36985
VU0359595	AOBIOUS	Cat#AOB1035
VU0364739 hydrochloride	Tocris	Cat#417110
TRIzol LS	Thermo	Cat#10296010
goat red blood cells	Innovative Research	Cat#IGTRBC100P15ML

Critical commercial assays		
In Fusion Cloning Kit	Takara	Cat#638948
KAPA RNA HyperPrep Kit with RiboErase (HMR)	Fisher Scientific	Cat#50-196-5257
Foxp3 / Transcription Factor Staining Buffer Set	Thermo	Cat# 00-5523-00
SLAMseq Kinetics Kit – Anabolic Kinetics Module	Lexogen	Cat# 061.24
SPRIselect Beads	Beckman Coulter	Cat# B23318
TransIT-293 Transfection Reagent	Mirus	Cat# MIR 2705
LIVE/DEAD™ Fixable Aqua Dead Cell Stain Kit	Thermo	Cat# L34957
LIVE/DEAD™ Fixable Blue Dead Cell Stain Kit	Thermo	Cat# L23105
**Deposited data**		
RNA-seq	This paper	GEO: GSE283550
SLAM-seq	This paper	GEO: GSE283550

Experimental models: Cell lines		
Yumm1.7	ATCC	Cat# CRL-3362
HEK293	ATCC	Cat# CRL-1573
RAW264.7	ATCC	Cat# TIB-71
Jurkat	ATCC	Cat# TIB-152
L-929	ATCC	Cat# CCL-1
HT115	Sigma	Cat# 85061104
MC38 and B16BL6	Yongqiang Feng Lab St. Jude Children's Research Hospital	N/A
THP-1	Paul Thomas Lab St. Jude Children's Research Hospital	N/A

Oligonucleotides		
sgRNA and shRNA sequences (see Methods)	This paper	N/A

Recombinant DNA		
pMXs-Notch1(1-1770aa)-mCherry-NLS	This paper	N/A
pMXs-Notch1(1-1770aa)-mNeongreen-NLS	This paper	N/A
pMXs-mNeongreen-PLD1	This paper	N/A
pMXs-Flag-Rubcn	(Boada-Romero et al., 2023)	N/A
pMXs-mCherry-Rubcn	(Boada-Romero et al., 2023)	N/A
Human Timd4-T2A-TagBFP lentiviral plasmid	This paper	N/A

References

Boada-Romero, E., Guy, C. S., Palacios, G., Mari, L., Li, Z., & Green, D. R. (2023). Phosphatidylserine clustering by membrane receptors triggers LC3-associated phagocytosis. *bioRxiv*, 2023.2009.2006.556449. doi:10.1101/2023.09.06.556449

Cunha, L. D., Yang, M., Carter, R., Guy, C., Harris, L., Crawford, J. C., . . . Green, D. R. (2018). LC3-Associated Phagocytosis in Myeloid Cells Promotes Tumor Immune Tolerance. *Cell, 175*(2), 429-441 e416. doi:10.1016/j.cell.2018.08.061

St. Jude - Confidential
