# Supplementary material for: Exclusion of Notch from the contact site during efferocytosis restricts anticancer immunity
Source: Nat Immunol. 2026 Mar 3;27(4):750–61. doi: 10.1038/s41590-026-02452-3 (PMC13043306; doi:10.1038/s41590-026-02452-3)
Supplement: Supplementary file 2 — Reporting Summary [file 41590_2026_2452_MOESM2_ESM.pdf]

nature portfolio

Corresponding author(s): Douglas R. Green

Last updated by author(s): Jan 7th, 2026

Reporting Summary

Nature Portfolio wishes to improve the reproducibility of the work that we publish. This form provides structure for consistency and transparency in reporting. For further information on Nature Portfolio policies, see our Editorial Policies and the Editorial Policy Checklist.

Please do not complete any field with "not applicable" or n/a. Refer to the help text for what text to use if an item is not relevant to your study.
For final submission: please carefully check your responses for accuracy; you will not be able to make changes later.

Statistics

For all statistical analyses, confirm that the following items are present in the figure legend, table legend, main text, or Methods section.

n/a	Confirmed	
☐	☒	The exact sample size (*n*) for each experimental group/condition, given as a discrete number and unit of measurement
☐	☒	A statement on whether measurements were taken from distinct samples or whether the same sample was measured repeatedly
☐	☒	The statistical test(s) used AND whether they are one- or two-sided<br>*Only common tests should be described solely by name; describe more complex techniques in the Methods section.*
☐	☒	A description of all covariates tested
☐	☒	A description of any assumptions or corrections, such as tests of normality and adjustment for multiple comparisons
☐	☒	A full description of the statistical parameters including central tendency (e.g. means) or other basic estimates (e.g. regression coefficient) AND variation (e.g. standard deviation) or associated estimates of uncertainty (e.g. confidence intervals)
☐	☒	For null hypothesis testing, the test statistic (e.g. *F*, *t*, *r*) with confidence intervals, effect sizes, degrees of freedom and *P* value noted<br>*Give P values as exact values whenever suitable.*
☒	☐	For Bayesian analysis, information on the choice of priors and Markov chain Monte Carlo settings
☒	☐	For hierarchical and complex designs, identification of the appropriate level for tests and full reporting of outcomes
☒	☐	Estimates of effect sizes (e.g. Cohen's *d*, Pearson's *r*), indicating how they were calculated

Our web collection on statistics for biologists contains articles on many of the points above.

Software and code

Policy information about availability of computer code

Data collection	BD FACSDiva software (LSRII and Fortessa) or SpectroFlo (Cytek Aurora)was used to collect flow cytometry data. Slidebook 6(3i) was used to collect confocal microscopy data. Li-Cor Oddyssey was used for immunoblot data acquisition.
Data analysis	Flowjo 10.8.2 for FACS; GraphPad 9.5.0 for statistics; slidebook 6 and Imaris x64 were used for microscope images; For RNA-seq: fastp (version 0.20.0); STAR (version 2.7.1a); HTSeq(v0.11.2); R(3.23); edgeR(3.12.1); limma (3.26.9); GSEA(v4.0.3); MSigDB(v7.4); For SLAM-seq: cutadapt(version 1.9); SLAMDUNK (version 0.4.0); R(3.23); edgeR(3.12.1); limma (3.26.9); GSEA(v4.0.3); MSigDB(v7.4); For Plotting: python(2.7); gseapy(0.9.3); R(3.6); ggplot2(3.4.1); ComplexHeatmap(2.15.4); For TCGA: cbioportal.org(TCGA PanCancer Atlas Studies 2024-12-20)

For manuscripts utilizing custom algorithms or software that are central to the research but not yet described in published literature, software must be made available to editors and reviewers. We strongly encourage code deposition in a community repository (e.g. GitHub). See the Nature Portfolio guidelines for submitting code & software for further information.

Data

Policy information about availability of data

All manuscripts must include a data availability statement. This statement should provide the following information, where applicable:

- Accession codes, unique identifiers, or web links for publicly available datasets
- A description of any restrictions on data availability
- For clinical datasets or third party data, please ensure that the statement adheres to our policy

RNA-seq and Slam-seq that support the findings of this study have been deposited in the Gene Expression Omnibus (GEO; https://www.ncbi.nlm.nih.gov/geo/) under accession number GSE283550. RSEM expression values (batch normalized from Illumina HiSeq RNASeqV2) were retrieved for TCGA PanCancer Atlas Studies (2024-12-20) at cbioportal.org. Code is deposited at https://doi.org/10.6084/m9.figshare.c.6186670.

Human research participants

Policy information about studies involving human research participants and Sex and Gender in Research.

Reporting on sex and gender	*Use the terms sex (biological attribute) and gender (shaped by social and cultural circumstances) carefully in order to avoid confusing both terms. Indicate if findings apply to only one sex or gender; describe whether sex and gender were considered in study design whether sex and/or gender was determined based on self-reporting or assigned and methods used. Provide in the source data disaggregated sex and gender data where this information has been collected, and consent has been obtained for sharing of individual-level data; provide overall numbers in this Reporting Summary. Please state if this information has not been collected. Report sex- and gender-based analyses where performed, justify reasons for lack of sex- and gender-based analysis.*
Population characteristics	*Describe the covariate-relevant population characteristics of the human research participants (e.g. age, genotypic information, past and current diagnosis and treatment categories). If you filled out the behavioural & social sciences study design questions and have nothing to add here, write "See above."*
Recruitment	*Describe how participants were recruited. Outline any potential self-selection bias or other biases that may be present and how these are likely to impact results.*
Ethics oversight	*Identify the organization(s) that approved the study protocol.*

Note that full information on the approval of the study protocol must also be provided in the manuscript.

Field-specific reporting

Please select the one below that is the best fit for your research. If you are not sure, read the appropriate sections before making your selection.

☒ Life sciences ☐ Behavioural & social sciences ☐ Ecological, evolutionary & environmental sciences

For a reference copy of the document with all sections, see nature.com/documents/nr-reporting-summary-flat.pdf

Life sciences study design

All studies must disclose on these points even when the disclosure is negative.

Sample size	No sample size calculation was performed to predetermine sample size. Sample size was selected to maximize the chance of uncovering mean difference which is also statistically significant.
Data exclusions	N/A
Replication	All the experimental finding were reproduced as validated by at least two independent experiments.
Randomization	Age-and sex-matched mice were assigned randomly to experimental and control groups. For other experiments samples are randomly located into experiment groups.
Blinding	The investigators were not blinded to group allocation during data collection or analysis, as there was no subjective measurement in our experiments. This approach is considered standard for experiments of the type performed in this study.

Reporting for specific materials, systems and methods

We require information from authors about some types of materials, experimental systems and methods used in many studies. Here, indicate whether each material, system or method listed is relevant to your study. If you are not sure if a list item applies to your research, read the appropriate section before selecting a response.

Materials & experimental systems

n/a	Involved in the study
☐	☒ Antibodies
☐	☒ Eukaryotic cell lines
☒	☐ Palaeontology and archaeology
☐	☒ Animals and other organisms
☒	☐ Clinical data
☒	☐ Dual use research of concern

Methods

n/a	Involved in the study
☒	☐ ChIP-seq
☐	☒ Flow cytometry
☒	☐ MRI-based neuroimaging

Antibodies

Antibodies used

anti-Notch2 Cell Signaling Technology Cat#5732S

anti-cleaved Notch1(Val1744) Cell Signaling Technology Cat# 4147

anti-TBP Cell Signaling Technology Cat#44059S
anti-Apaf-1 Cell Signaling Technology Cat#8969S
anti-Rubcn Cell Signaling Technology Cat#8465
anti-Atg5 Cell Signaling Technology Cat#12994
anti-beta actin HRP conjugated Santa Cruz Cat#sc-47778
anti-Jagged1 Cell Signaling Technology Cat#70109T
anti-Lamin B1 Cell Signaling Technology Cat#12586S
anti-PIK3R4/VPS15 Cell Signaling Technology Cat#14580
anti-PIK3C3/VPS34 (Clone D9A5) Cell Signaling Technology Cat#4263
anti-Beclin1 Cell Signaling Technology Cat#3738
anti-Uvrag Cell Signaling Technology Cat#13115S
anti-Atg14 Cell Signaling Technology Cat#96752
anti-NOX2/gp91-phox Santa Cruz Cat#sc-130543
anti-Pld1 Cell Signaling Technology Cat#3832S
Anti-PDI Cell Signaling Technology Cat#3501
Anti-RCAS1 Cell Signaling Technology Cat#12290
Anti-Dll1 Abcam Cat#ab85346
anti-human CD18/CR3 Dylight 488 (clone KIM127) Leinco Technologies Cat#C565
Human IgG Sigma Cat#12511
anti-human IgG Alexa Fluor647 Jackson ImmunoResearch Cat#709-605-149
PerCP/Cy5.5 anti-mouse TCRb BioLegend Cat#109228
Alexa Fluor647 anti-human/mouse GranzymeB BioLegend Cat#515405
BV605 anti-mouse NK1.1 BioLegend Cat#108739
BV421 anti-mouse CD45 BioLegend Cat#103133
BUV737 anti-mouse CD4 BD Biosciences Cat#612844
BUV805 anti-mouse CD8α BD Biosciences Cat#612898
PE anti-mouse IFNg BD Biosciences Cat#505808
PE-Cy7 anti-mouse TNFa BD Biosciences Cat#561041
BV785 anti-mouse CD11b BioLegend Cat#101243
FITC anti-mouse MHC-II eBioscience Cat#11-5321-82
PE-CF594 anti-Mouse CD11c BD Biosciences Cat#562454
APC-Fire750 anti-mouse CD45 BioLegend Cat#147714
eF450 anti-mouse CD45.2 eBioscience Cat#48-0454-82
FITC anti-mouse TCRb BD Biosciences Cat#553170
BUV737 anti-Mouse CD8 BD Biosciences Cat#741811
BUV395 anti-Mouse CD4 BD Biosciences Cat#563790
PerCP-eF710 anti-mouse MHC-II eBioscience Cat#46-5321-82
PE-Cy7 anti-mouse CD11c eBioscience Cat#25-0114-82
BUV661 anti-mouse CD11b BD Biosciences Cat#612977
Spark YG 593 anti-mouse F4/80 BioLegend Cat#157311
BV570 anti-mouse Ly6c BioLegend Cat#128029
APC/Fire 810 anti-mouse NK-1.1 BioLegend Cat#156519
APC anti-mouse CD45 BD Bioscience Cat#559864
Alexa Fluor 532 anti-mouse TCRb eBioscience Cat#58-5961-82
PerCP anti-mouse CD8a BioLegend Cat#100732
PE-Cy5 anti-mouse Granzyme-B BioLegend Cat#372226
PerCP-Cy 5.5 anti-mouse IL-2 eBioscience Cat#45-7021-82
APC/Cy7 anti-mouse TNFα BD Bioscience Cat#506344
APC anti-mouse F4/80 Invitrogen Cat#17-4801-82
PEcy7 anti-mouse Gr1 Biolegend Cat#108416

A lexa Fluor647 Annexin V Biolegend Cat#640911

Validation

Antibodies against Notch2, Rubcn, ATG5, VPS34, UVRAG, ATG14, PLD1were validated by CRISPR-Cas9-based knock-out in cell lines with western immunoblot method. Other antibodies for imaging and flow cytometry are validated by manufacturer.

St. Jude - Confidential

Eukaryotic cell lines

Policy information about cell lines and Sex and Gender in Research

Cell line source(s)	Cell lines Yumm1.7 (Cat no. CRL-3362), HEK293 (Cat no. CRL-1573), RAW264.7 (Cat No. TIB-71), Jurkat (Cat no. TIB-152), CHO-K1 (CCL-61) and L-929 (Cat. No. CCL-1) were purchased from ATCC. HT115 cells (Cat no. 85061104) were purchased from Sigma. MC38 (mouse colon carcinoma) and B16BL6 (mouse melanoma) cells were a gift from Dr. Yongqiang Feng lab (St. Jude Children's Research Hospital, Memphis, TN, USA). The THP-1 cell line is a gift from Dr. Paul Thomas (St. Jude Children's Research Hospital, Memphis, TN, USA).
Authentication	The cell line used was not authenticated
Mycoplasma contamination	All cell lines were checked for mycoplasma contamination and found to be negative.
Commonly misidentified lines (See ICLAC register)	No commonly misidentified cell line were used.

Animals and other research organisms

Policy information about studies involving animals; ARRIVE guidelines recommended for reporting animal research, and Sex and Gender in Research

Laboratory animals	Mice were housed and bred at the St. Jude Children's Research Hospital Animal Resource Center in specific pathogen-free conditions. Mice were on 12-hour light/dark cycles that coincide with daylight in Memphis, TN, USA. The St. Jude Children's Research Hospital Animal Resource Center housing facility was maintained at 20–25 °C and 30–70 % humidity. All genetic models were on the C57BL/6 background. Both male and female mice were used for analysis and quantification. All mice were used at 2-6 months old.
Wild animals	This study did not involve wild animals
Reporting on sex	Sex of the mice was not considered during experimental planning.
Field-collected samples	The study did not involve samples collected from the field.
Ethics oversight	Mouse studies were conducted in accordance with protocols approved by the St. Jude Children's Research Hospital Committee on Care and Use of Animals and in compliance with all relevant ethical guidelines.

Note that full information on the approval of the study protocol must also be provided in the manuscript.

Flow Cytometry

Plots

Confirm that:

☒ The axis labels state the marker and fluorochrome used (e.g. CD4-FITC).

☒ The axis scales are clearly visible. Include numbers along axes only for bottom left plot of group (a 'group' is an analysis of identical markers).

☒ All plots are contour plots with outliers or pseudocolor plots.

☒ A numerical value for number of cells or percentage (with statistics) is provided.

Methodology

Sample preparation	The spleens were gently separated under nylon mesh using the flat end of a 3-mL syringes. Red blood cells were removed by ACK lysing buffer, followed by washing cells with isolation buffer. After spinning down, the cell pellets were resuspended and filtered with nylon mesh before staining.<br>For the examination of tumour infiltrating lymphocytes, tumours were excised, minced and digested with 0.5 mg/ml Collagenase IV (Roche) + 200 UI/ml DNase I (Sigma) for 40 min at 37 °C, and then passed through 70-μm filters to remove undigested tumor tissues.
Instrument	Fortessa (BD Bioscience) or Aurora (Cytek).
Software	Flowjo 10.8.2
Cell population abundance	*Describe the abundance of the relevant cell populations within post-sort fractions, providing details on the purity of the samples and how it was determined.*
Gating strategy	Based on the pattern of FSC-A/SSC-A, cells in the lymphocyte gate were used for analysis of T cell and myeloid cell subsets. Singlets were gated

according to the pattern of FSC-H vs. FSC-A. Positive populations were determined by the specific antibodies, which were distinct from negative populations.

☒ Tick this box to confirm that a figure exemplifying the gating strategy is provided in the Supplementary Information.

This checklist template is licensed under a Creative Commons Attribution 4.0 International License, which permits use, sharing, adaptation, distribution and reproduction in any medium or format, as long as you give appropriate credit to the original author(s) and the source, provide a link to the Creative Commons license, and indicate if changes were made. The images or other third party material in this article are included in the article's Creative Commons license, unless indicated otherwise in a credit line to the material. If material is not included in the article's Creative Commons license and your intended use is not permitted by statutory regulation or exceeds the permitted use, you will need to obtain permission directly from the copyright holder. To view a copy of this license, visit http://creativecommons.org/licenses/by/4.0/
