# Supplementary material for: Exclusion of Notch from the contact site during efferocytosis restricts anticancer immunity
Source: Nat Immunol. 2026 Mar 3;27(4):750–61. doi: 10.1038/s41590-026-02452-3 (PMC13043306; doi:10.1038/s41590-026-02452-3)
Supplement: Supplementary file 17 — Uncropped and unprocessed blots. [file 41590_2026_2452_MOESM17_ESM.pdf]

Figure 1e

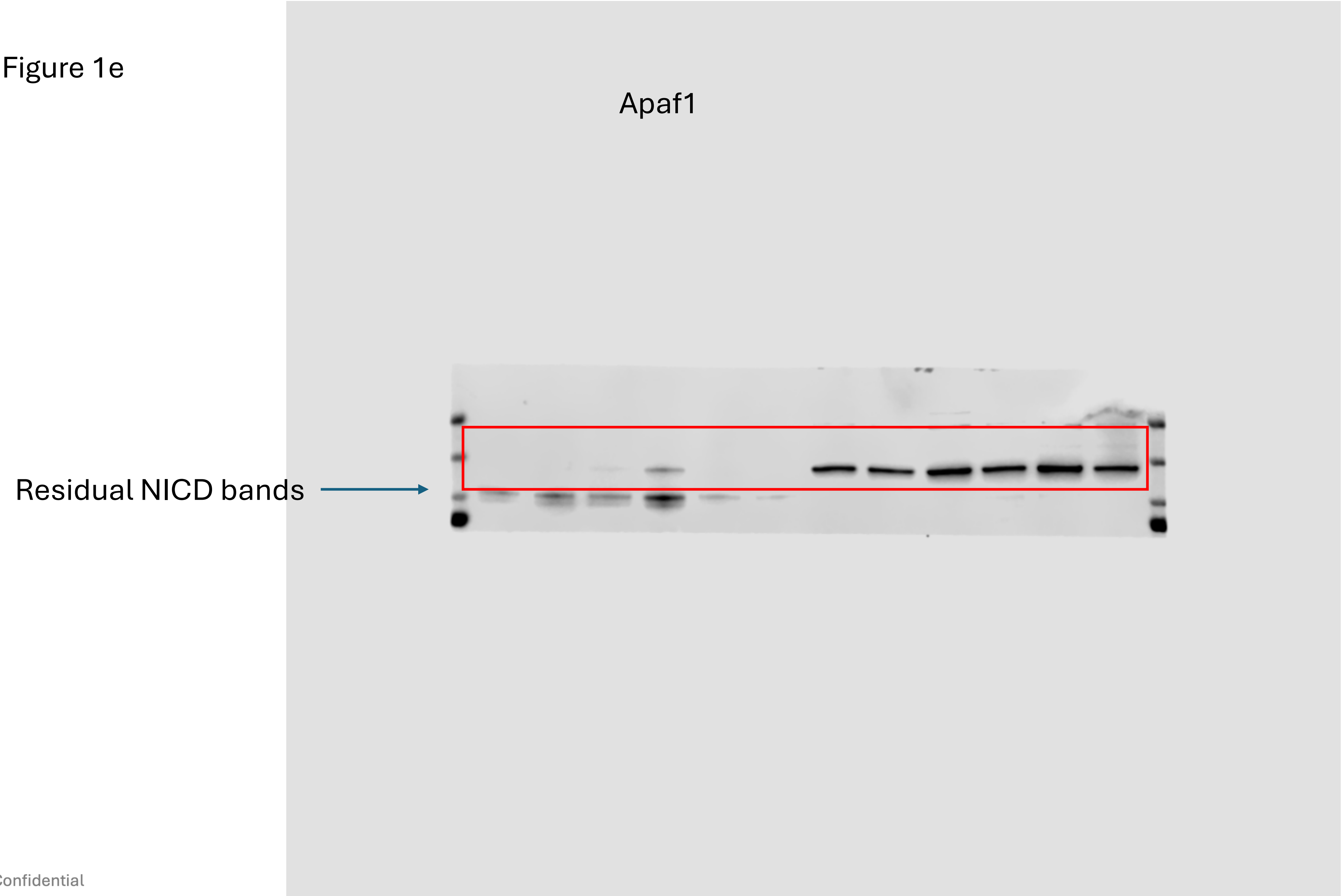

Figure 1e

N2ICD

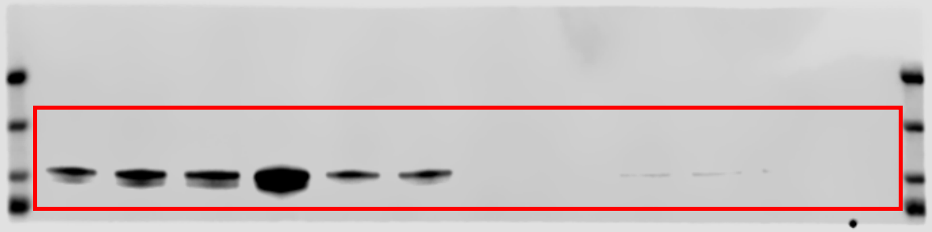

Figure 1e

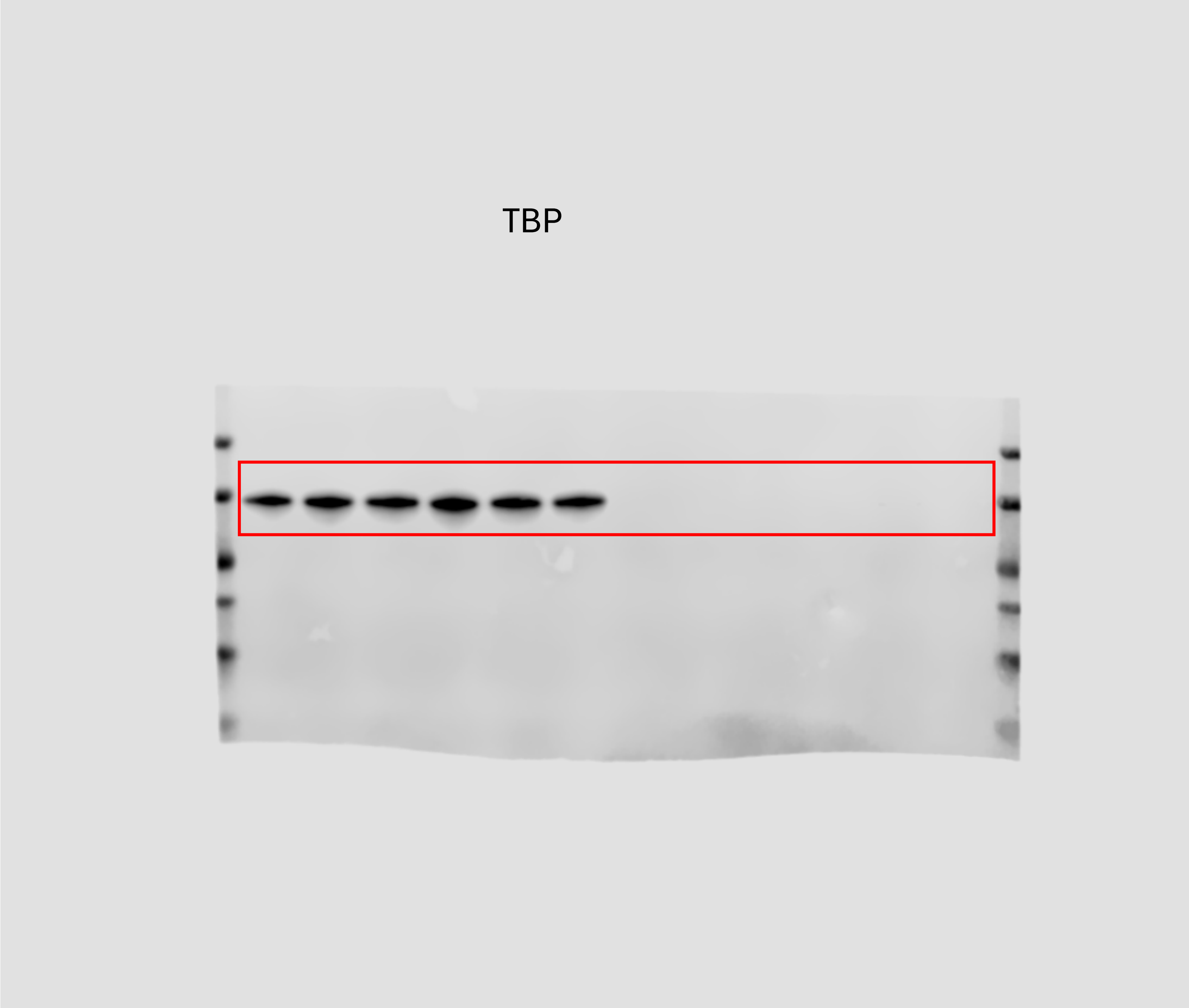

Figure 1f

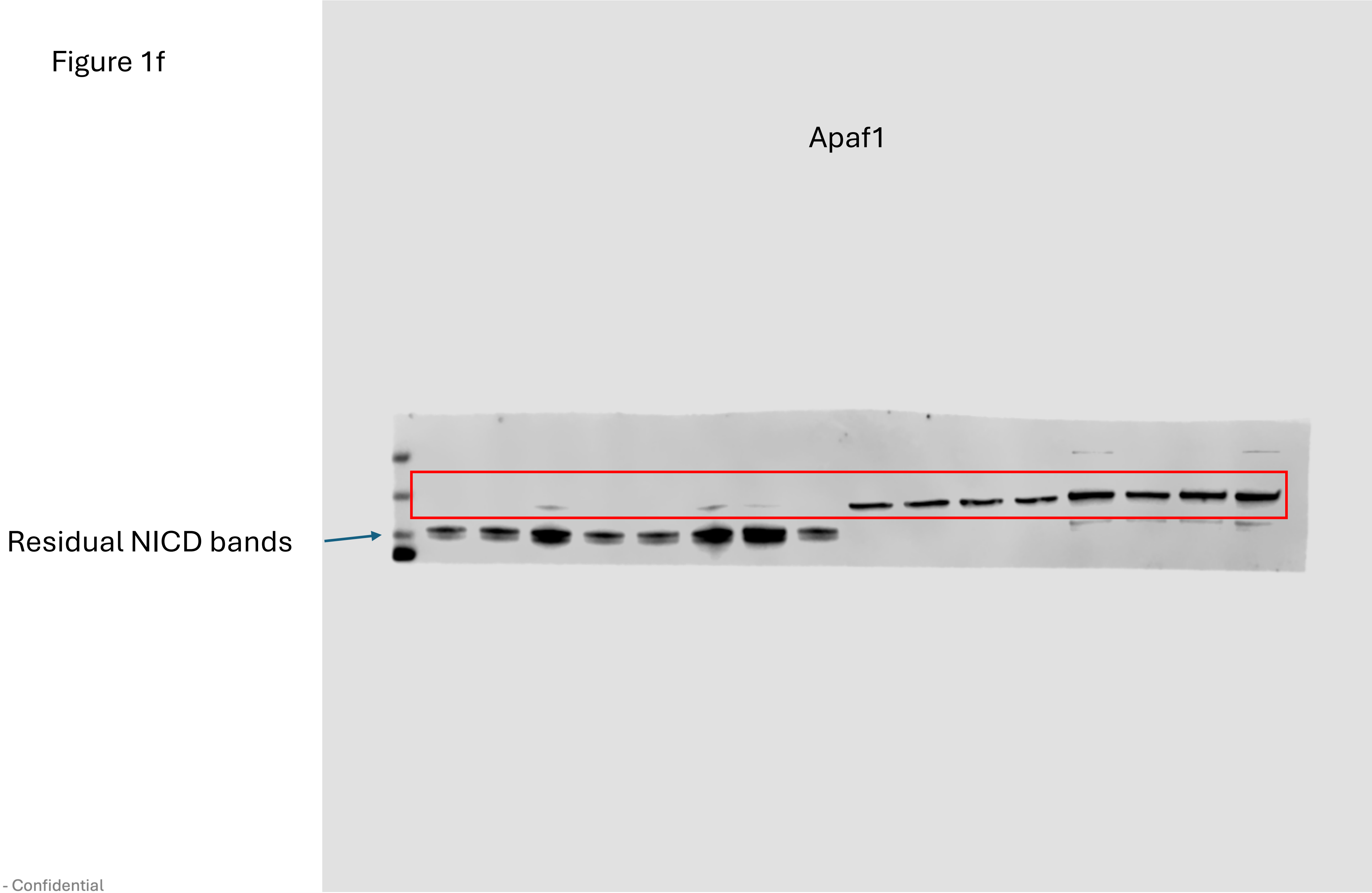

Figure 1f

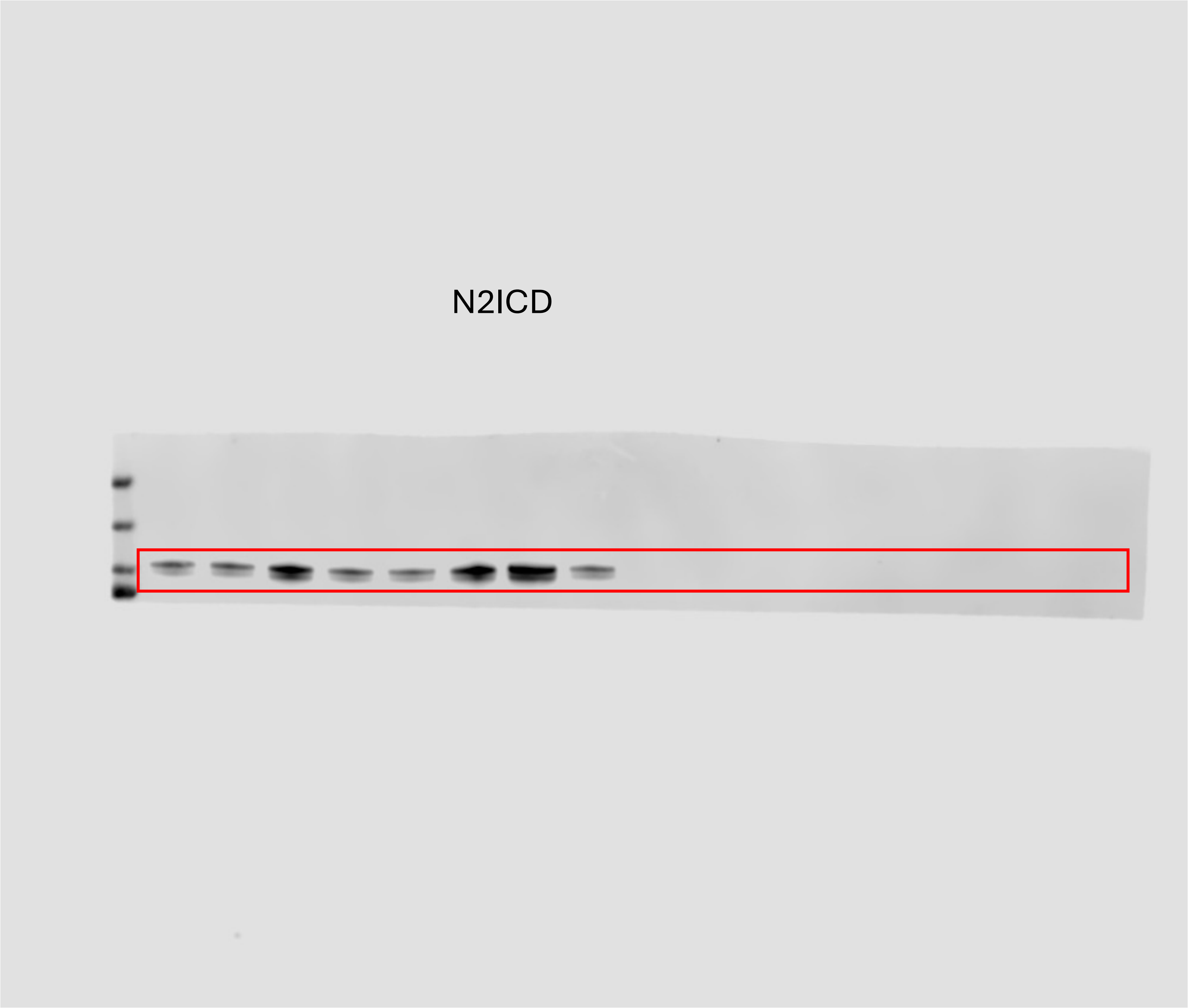

Figure 1f

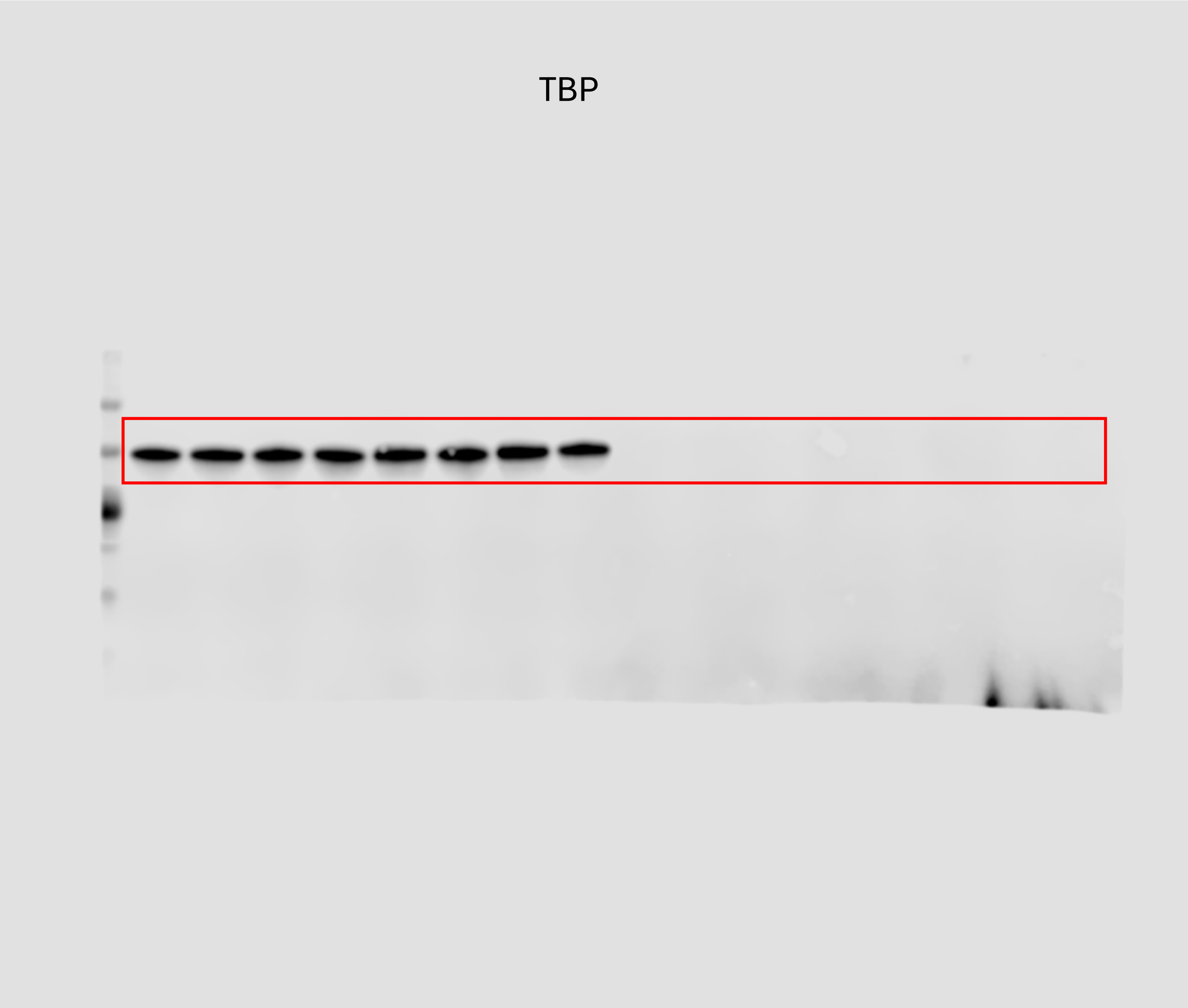

Extended data 1a

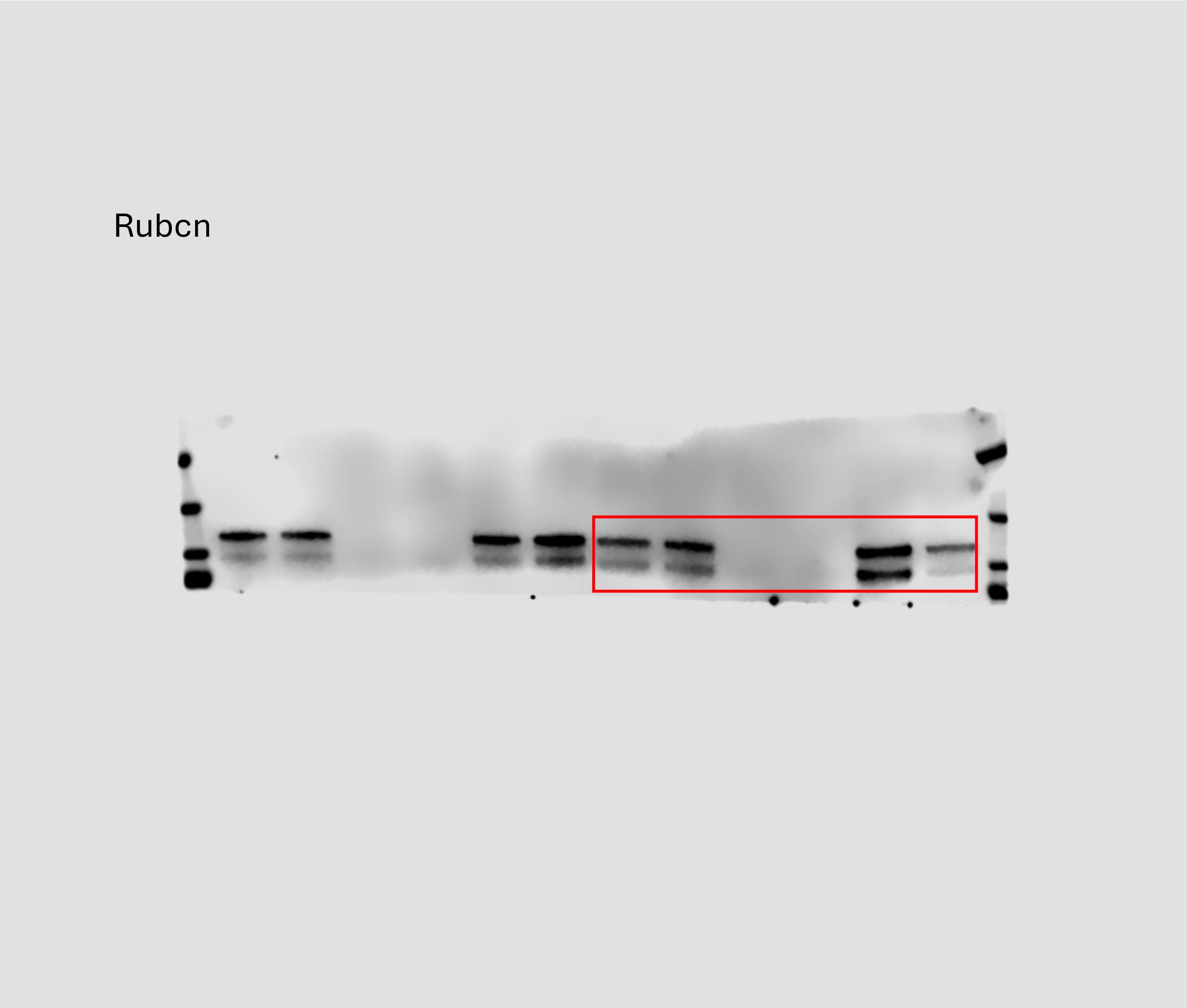

Extended data 1a

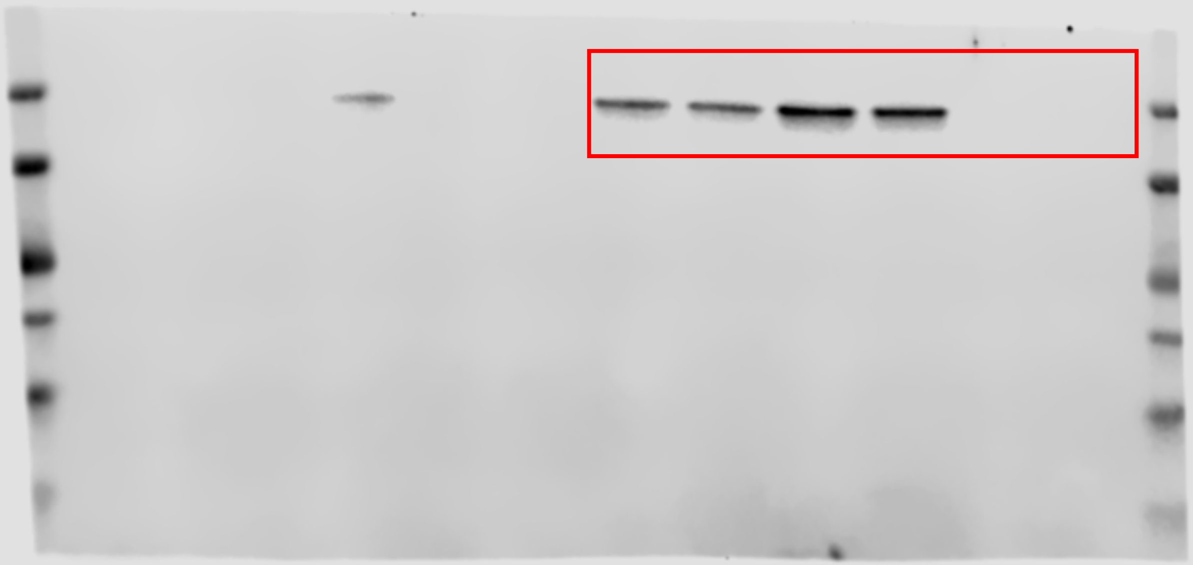

Extended data 1a

actin

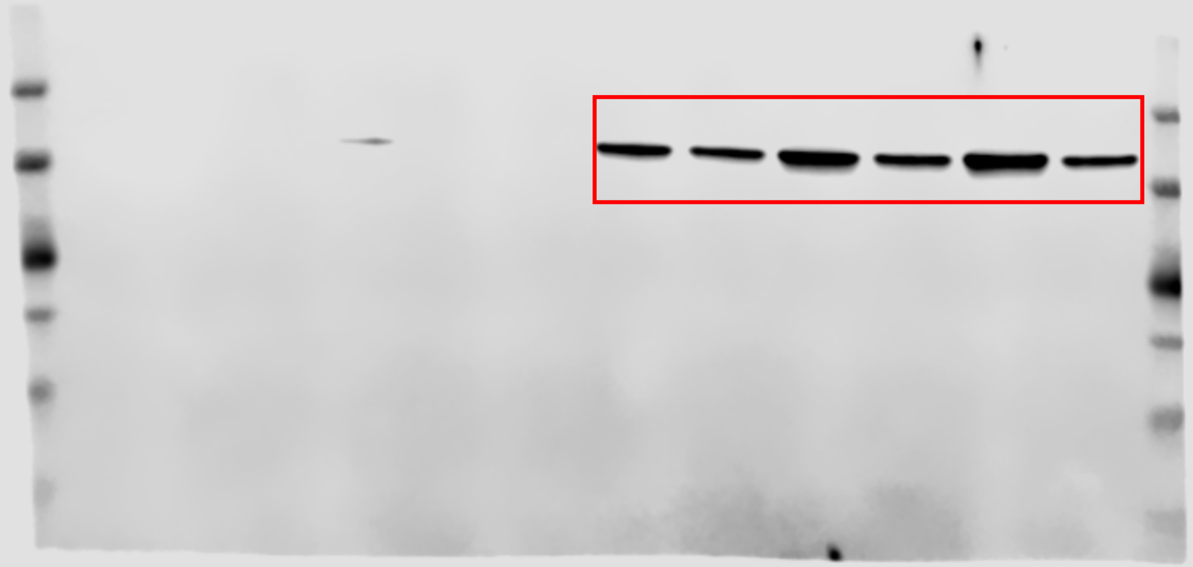

Extended Data 2a

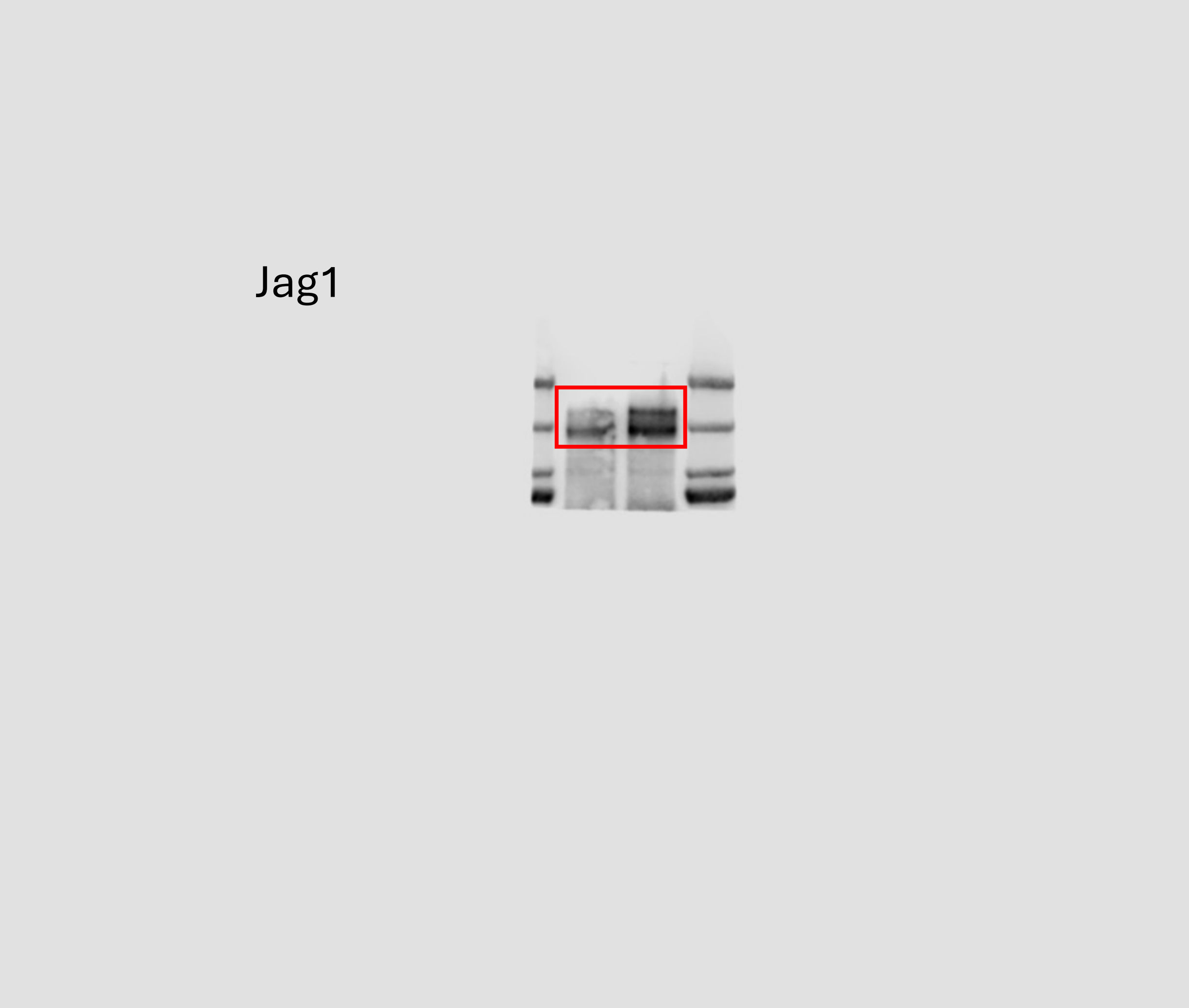

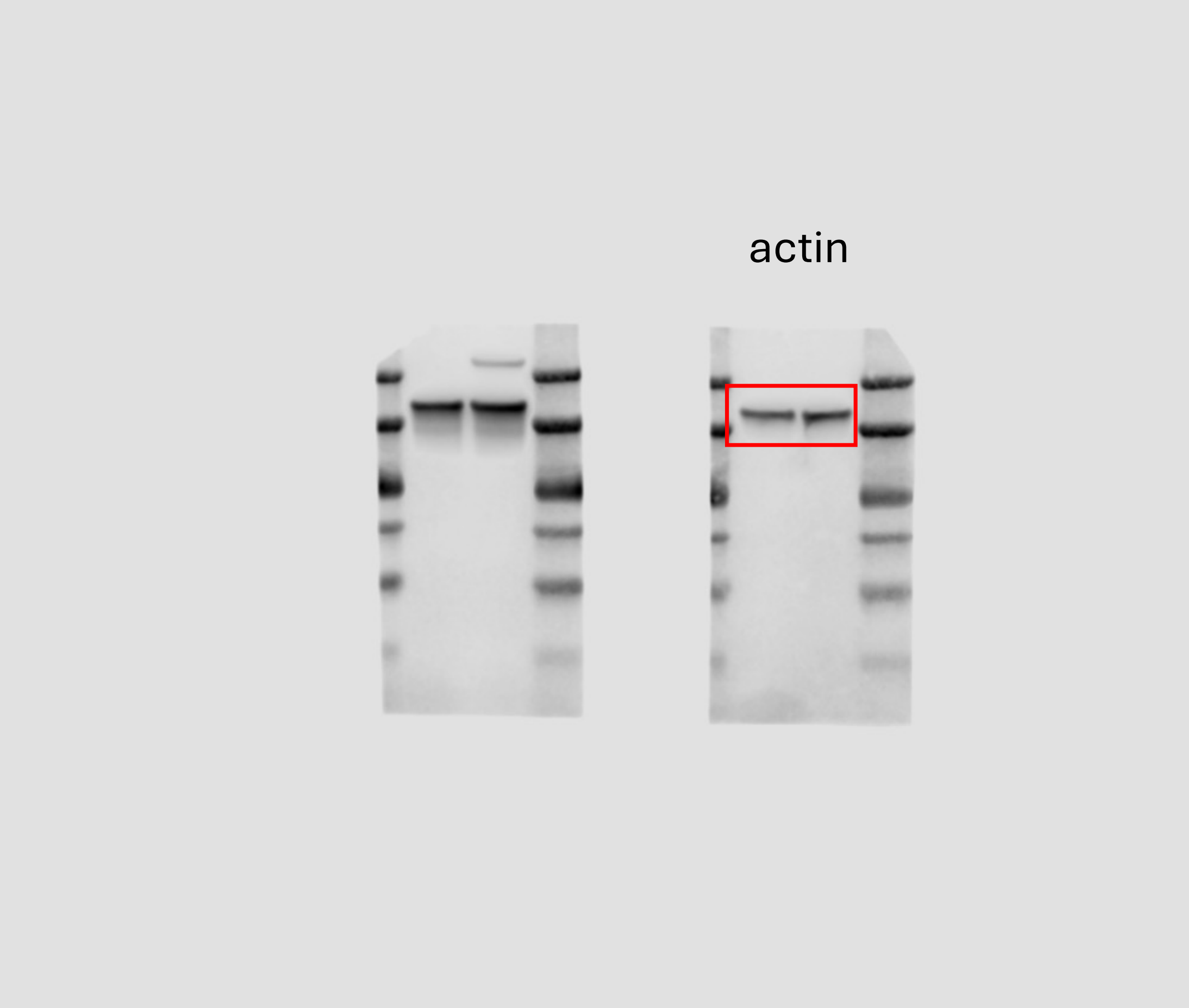

Extended Data Fig. 2b

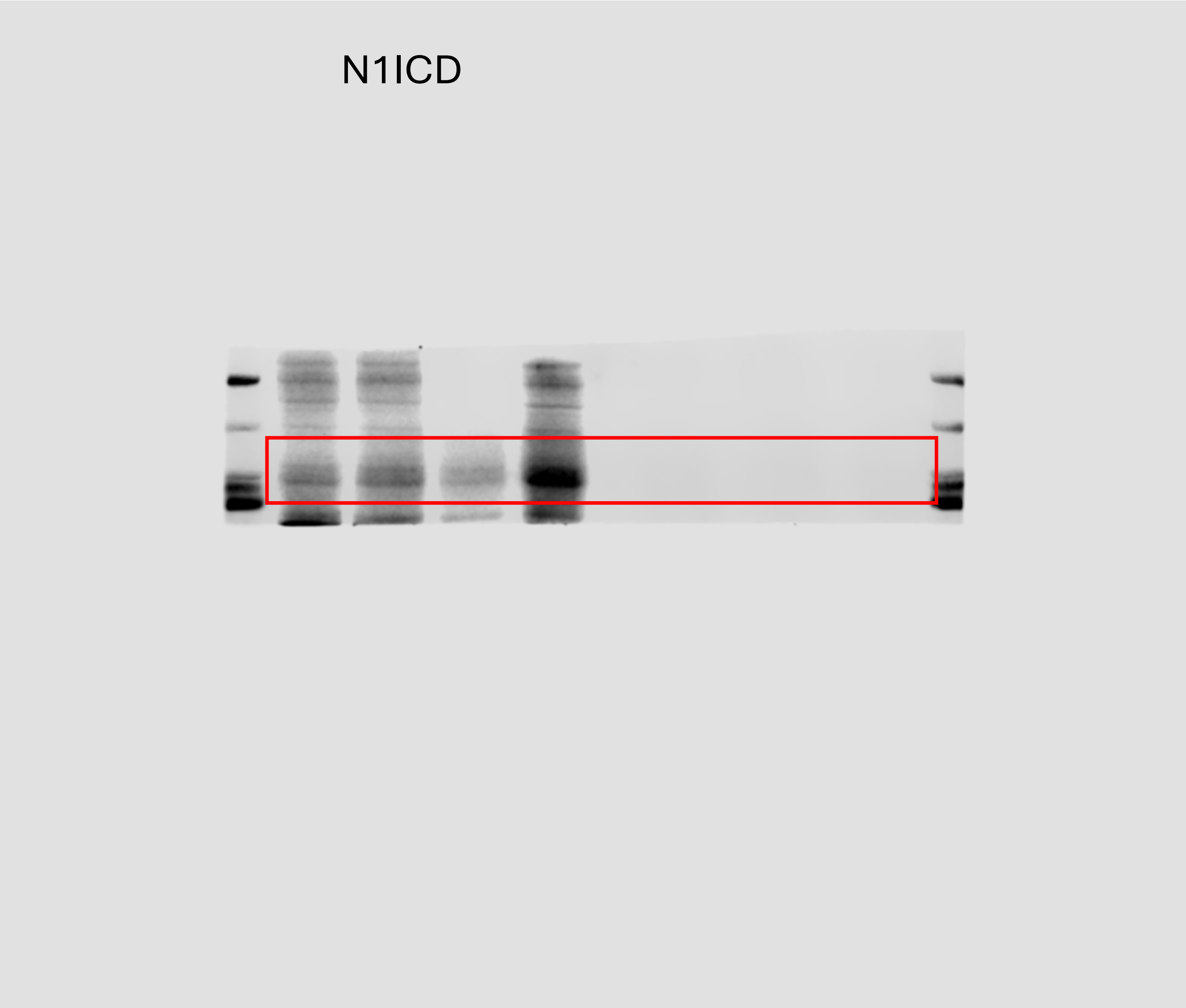

Extended Data Fig. 2b

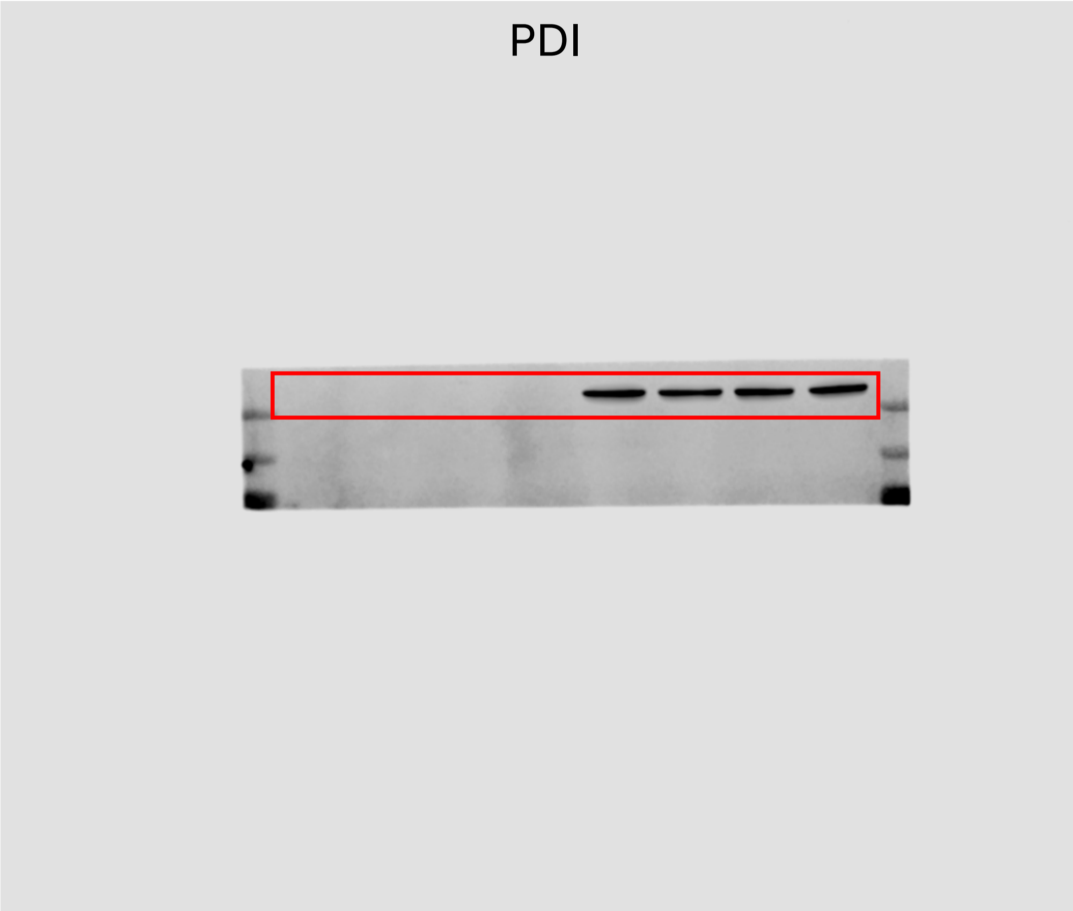

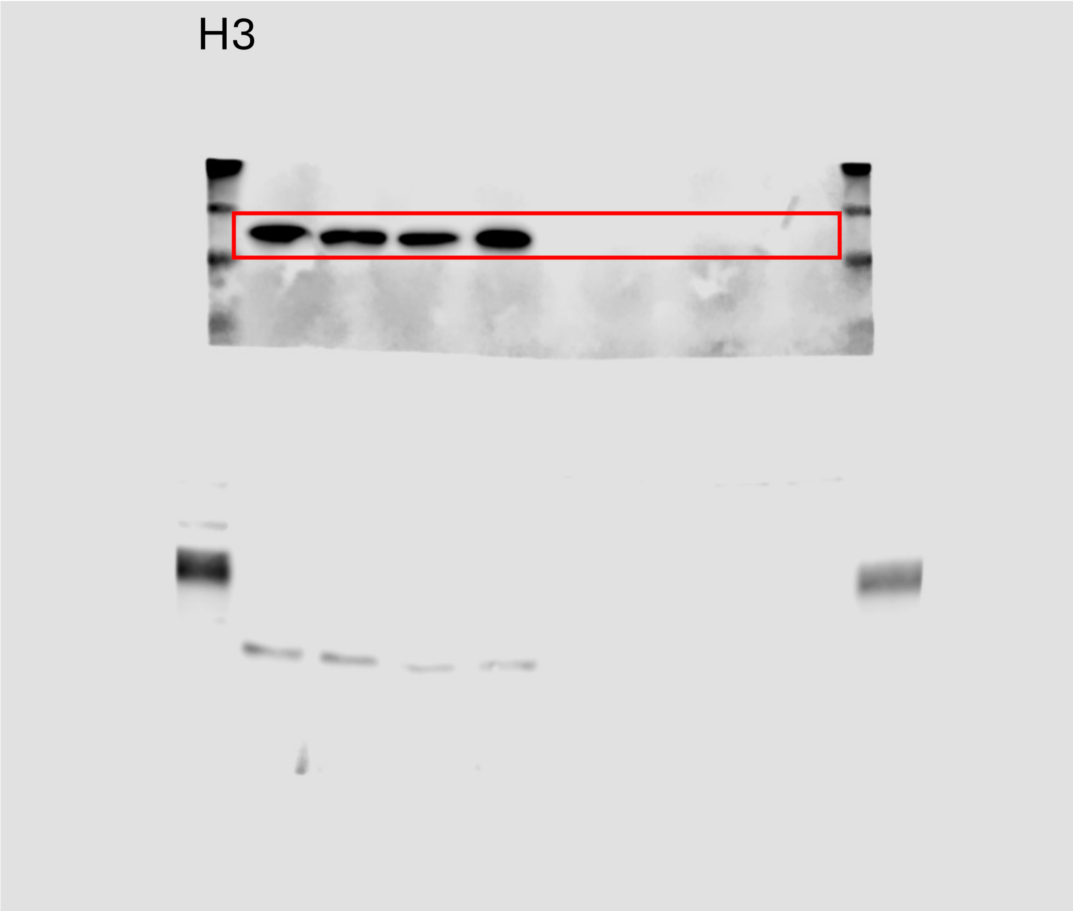

Extended Data Fig. 2c

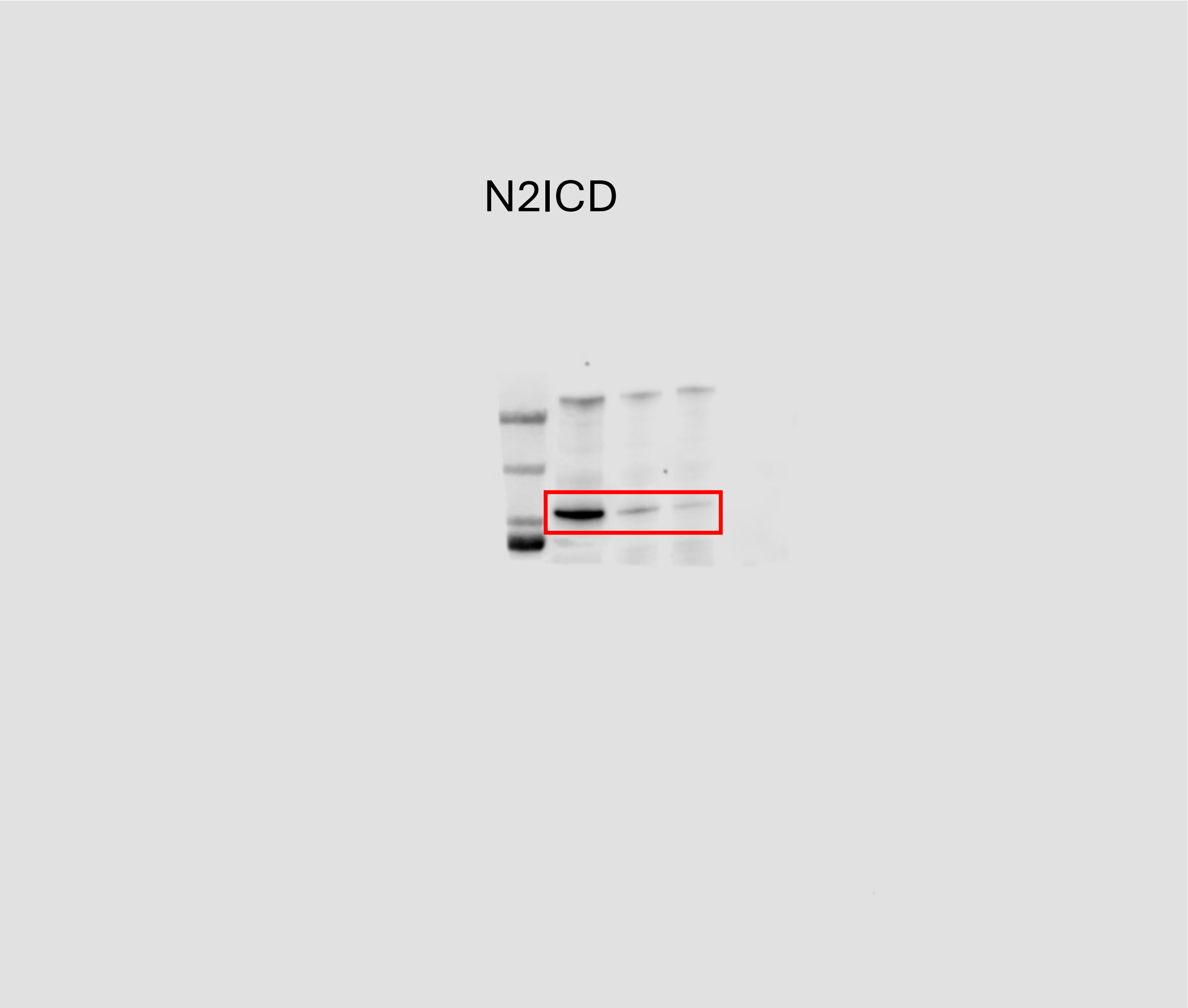

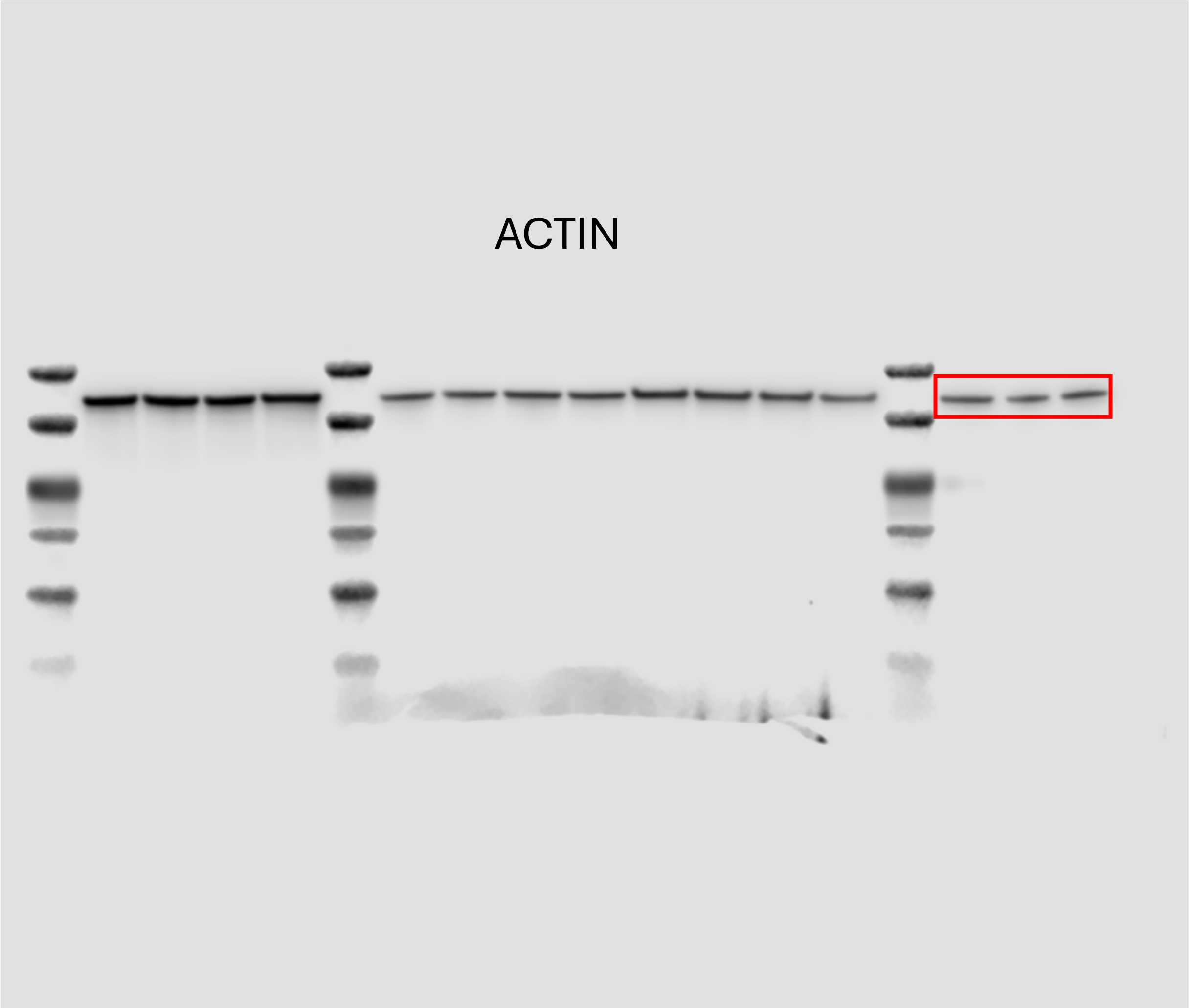

Extended Data Fig.2d

N2ICD

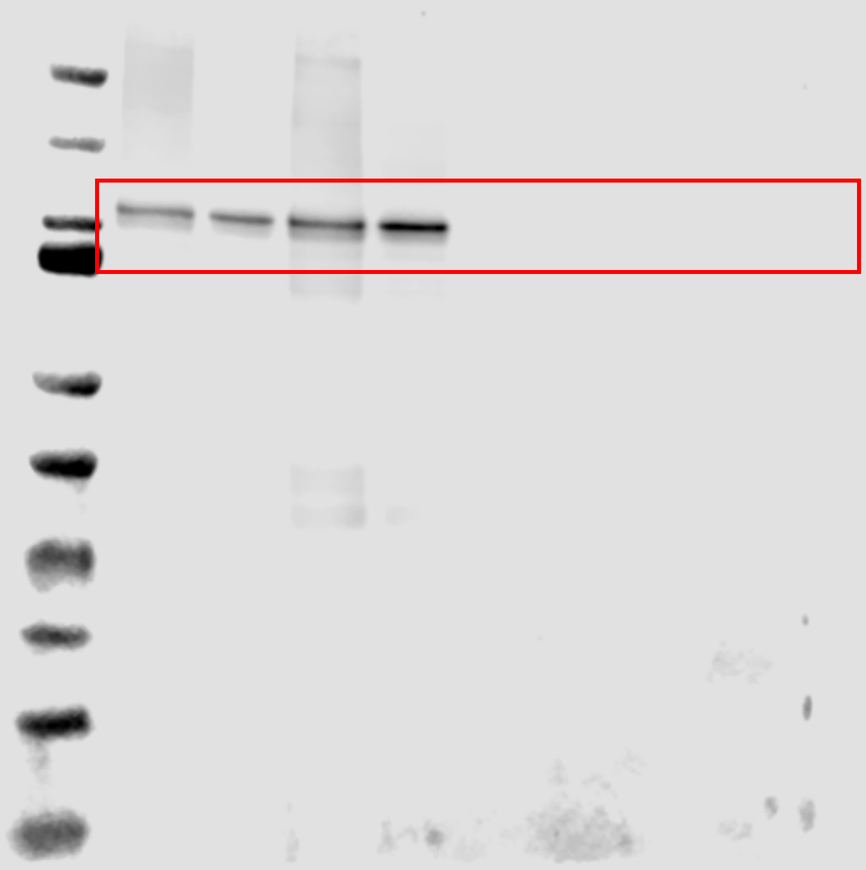

Extended Data Fig.2d

lamb1

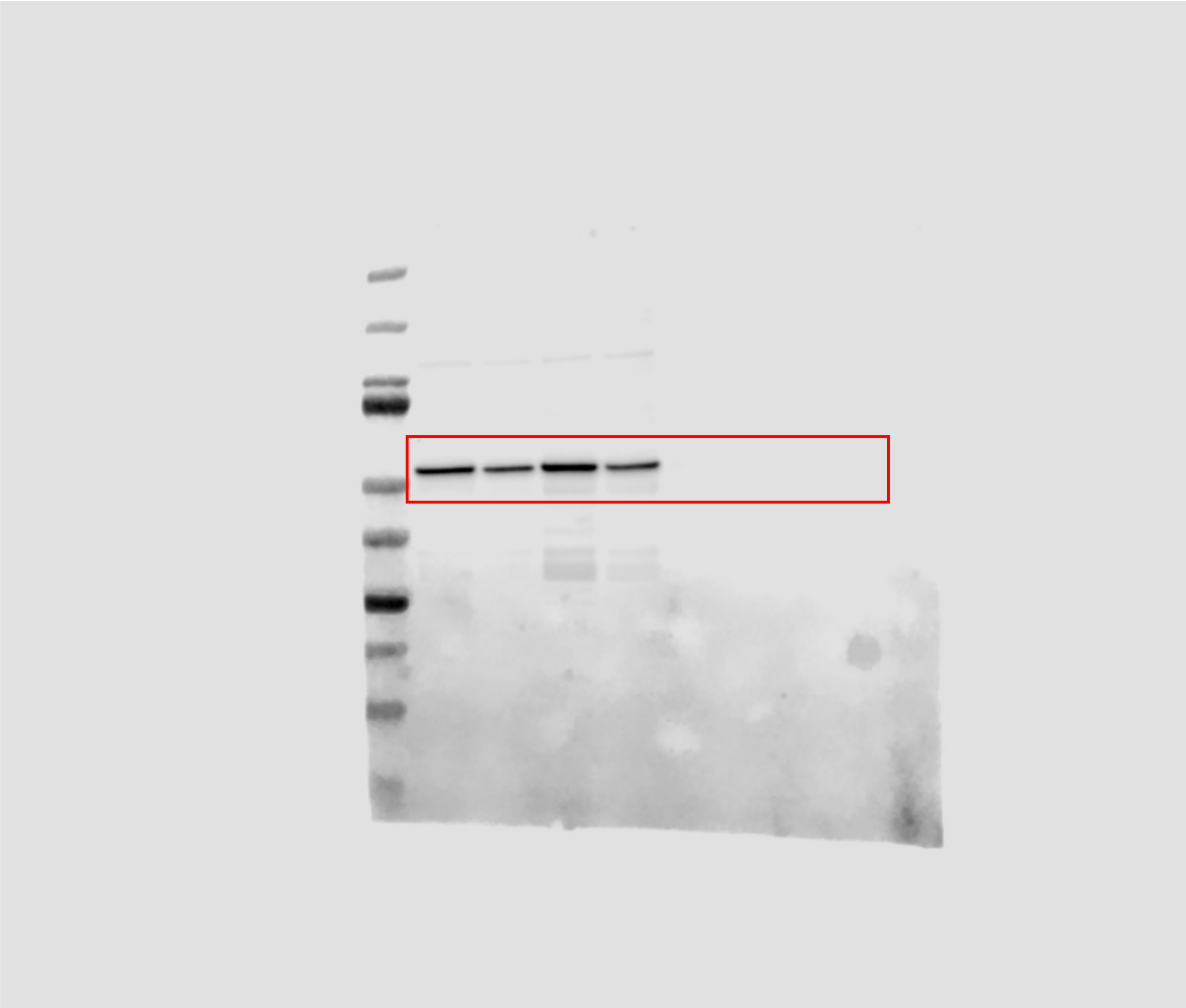

Extended Data Fig.2d

Apaf1

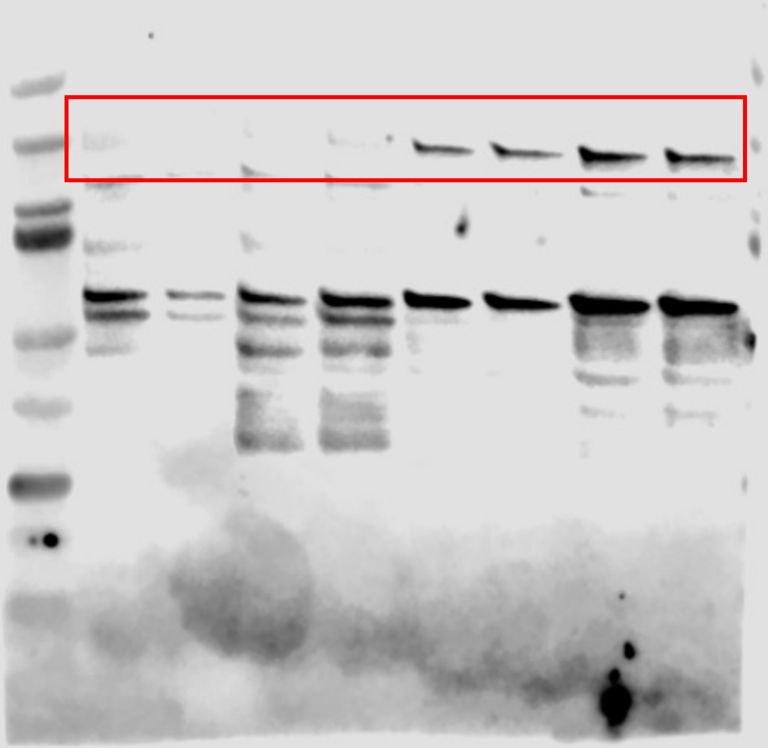

Extended Data Fig. 2f

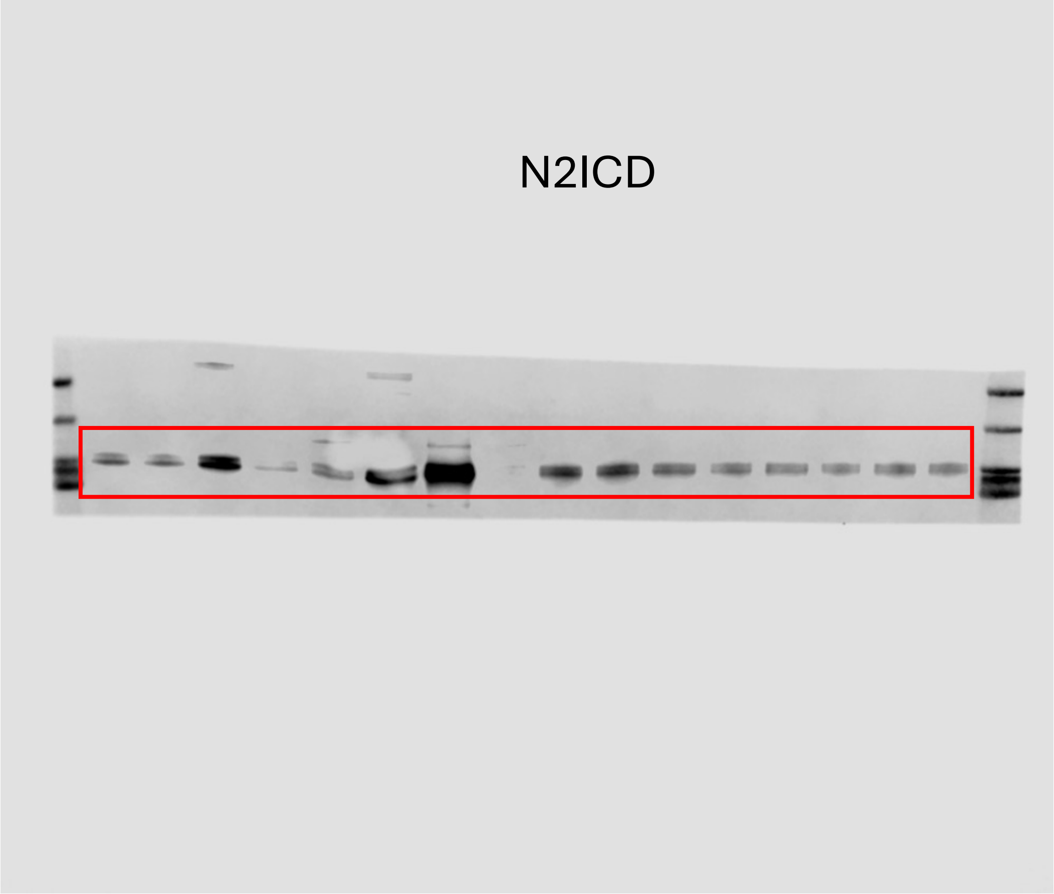

Extended Data Fig. 2f

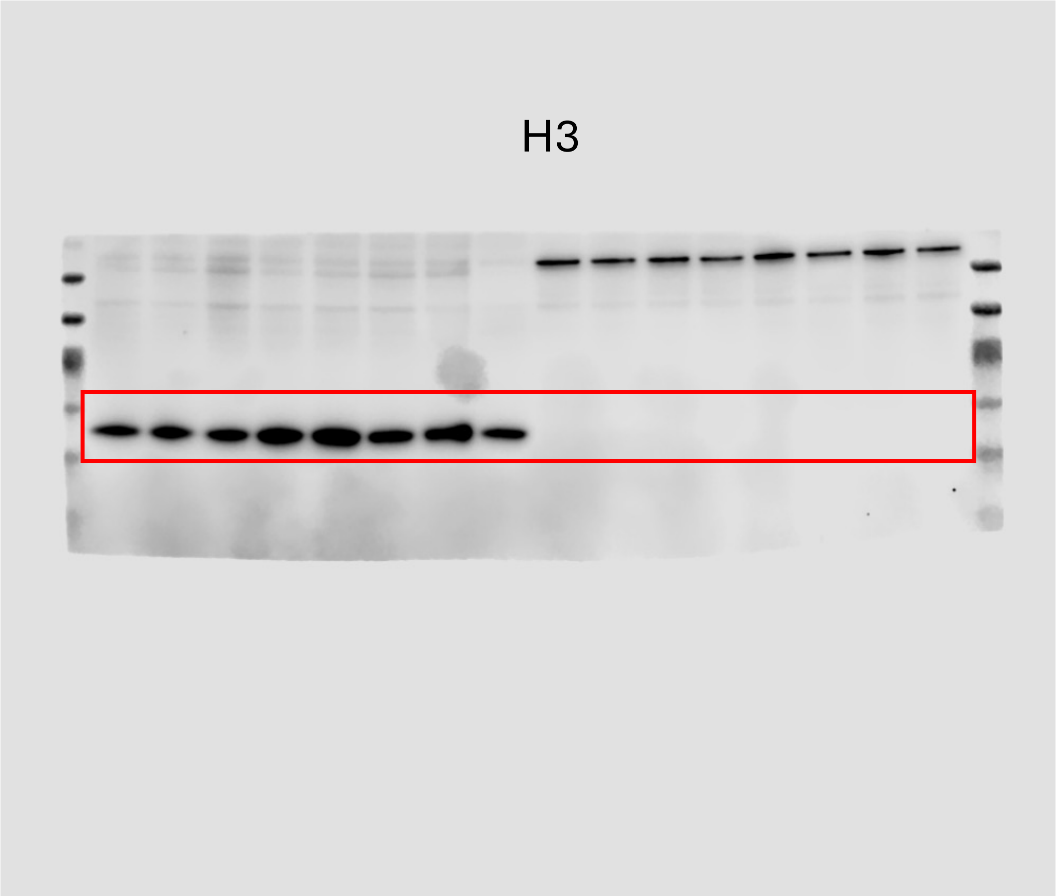

Extended Data Fig. 2f

PDI

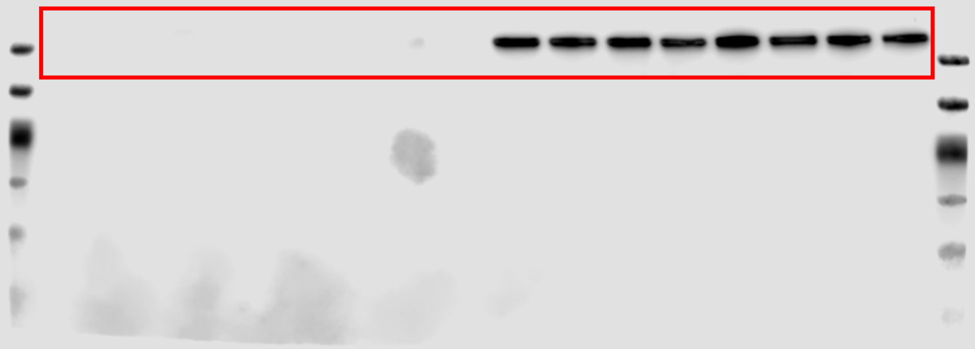

RACS1

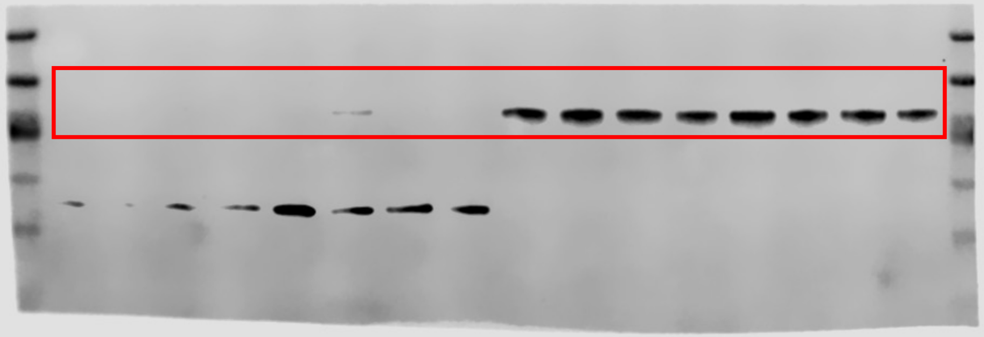

Extended Data Fig2h

N2ICD

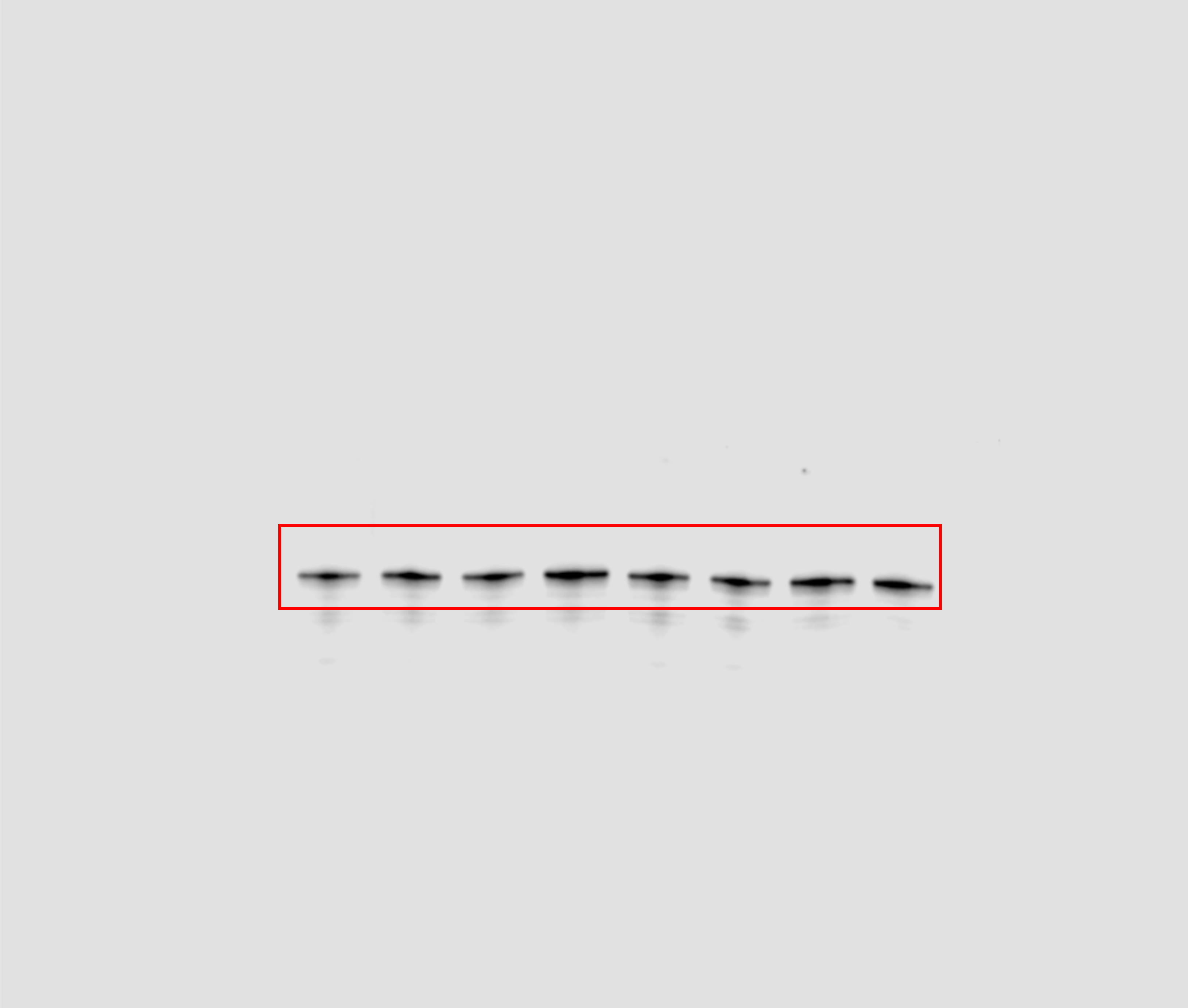

Extended Data Fig2h

TBP

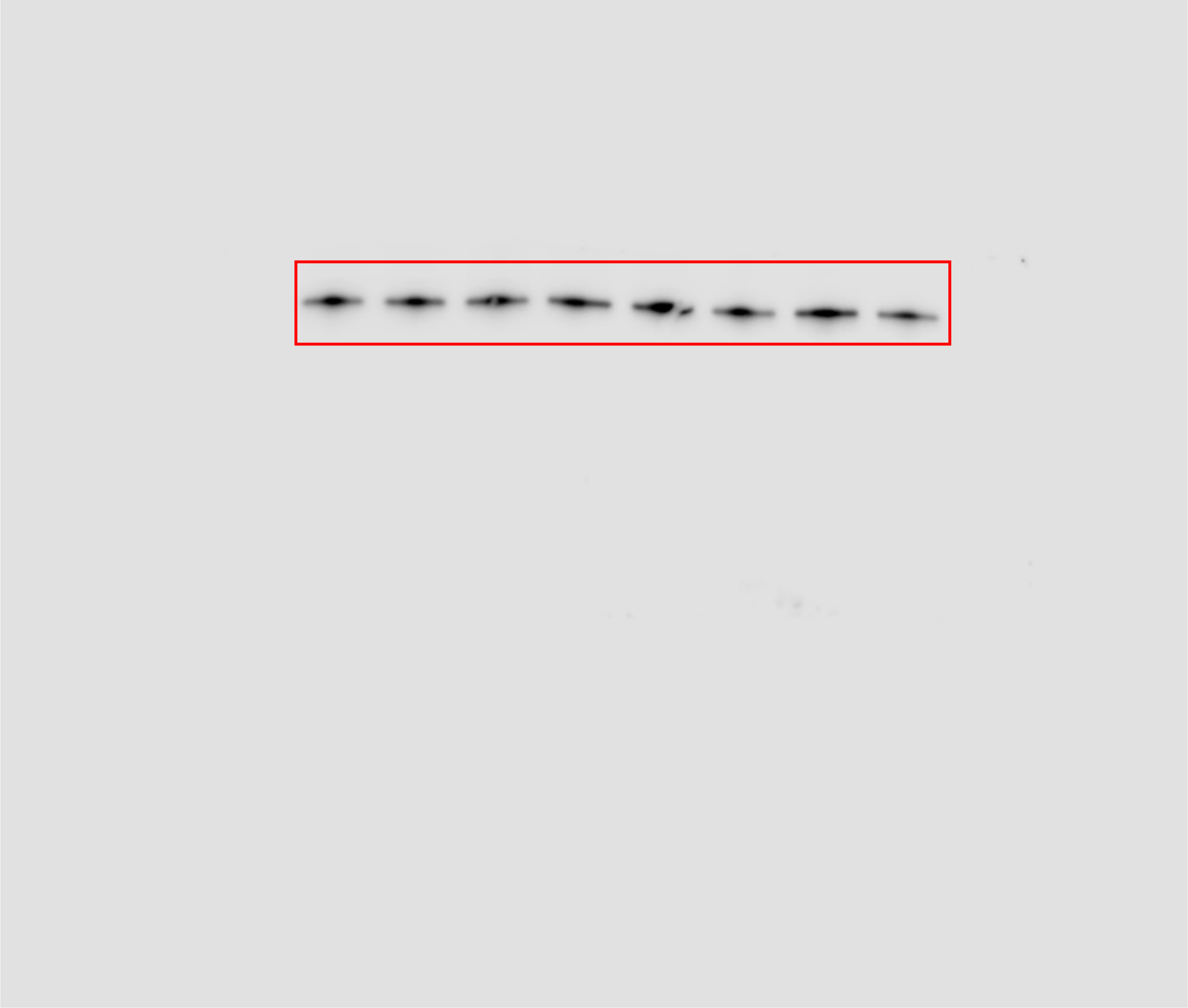

Extended Data Fig. 2i

N2ICD

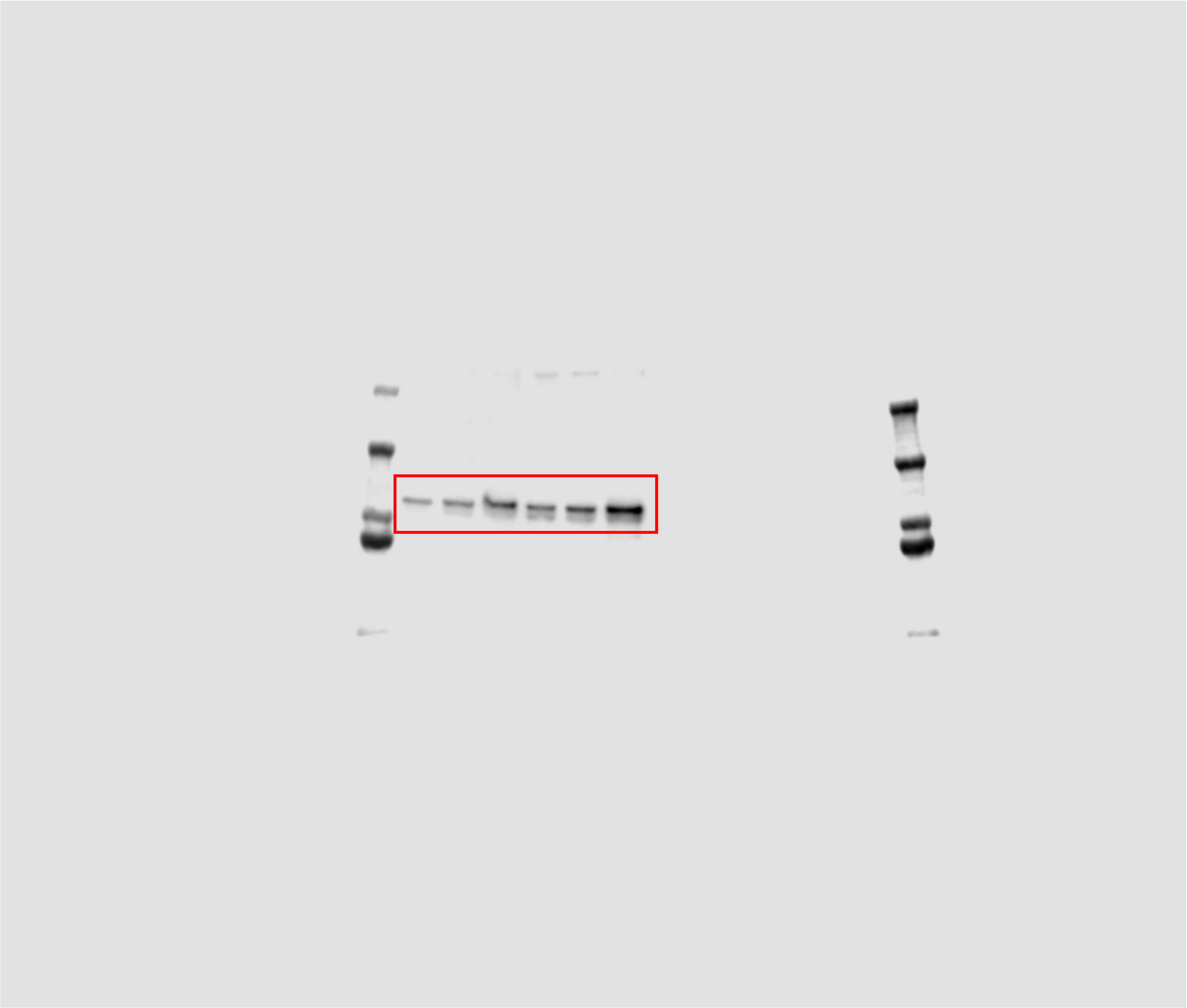

Extended Data Fig. 2i

TBP

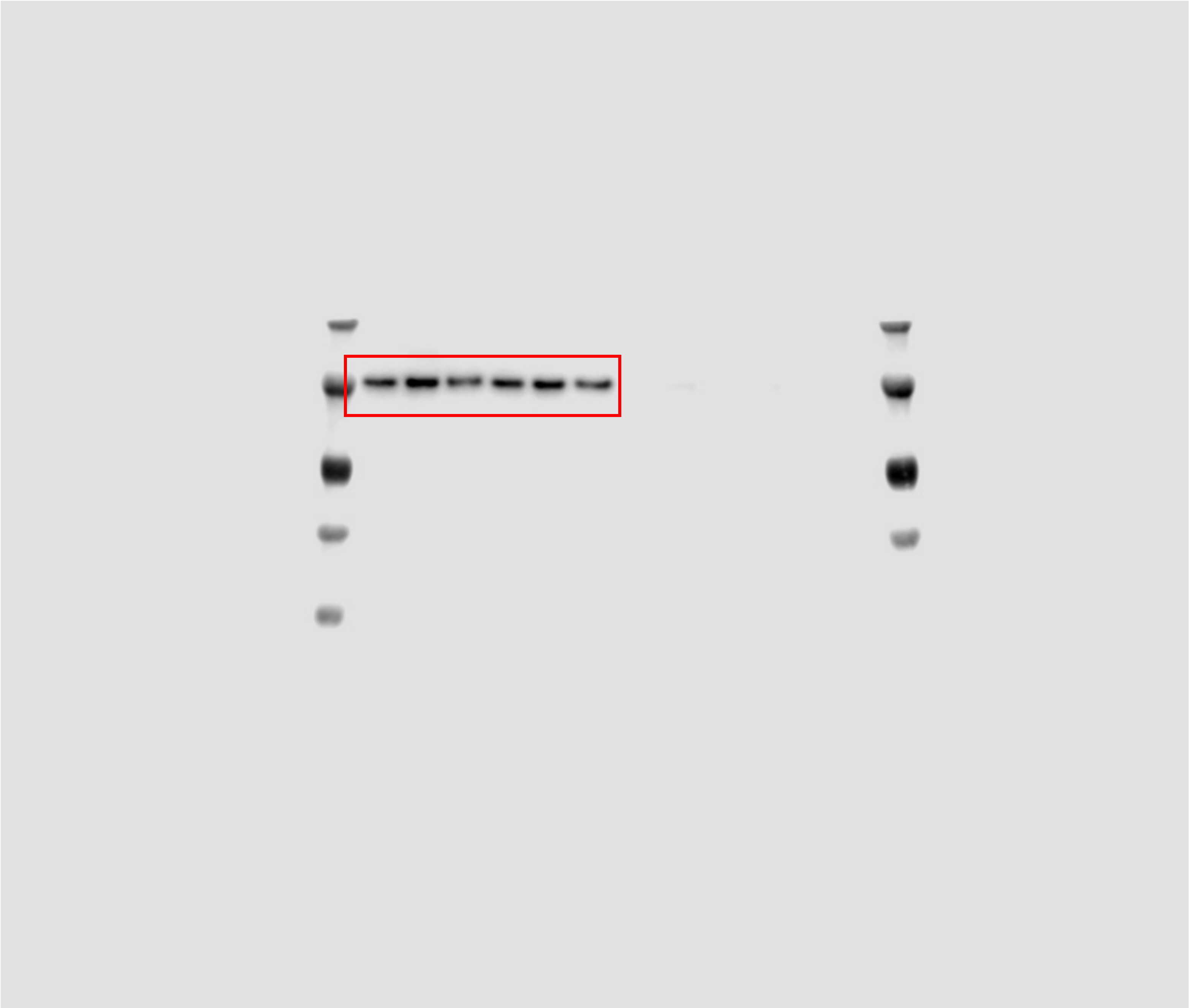

Extended Data Fig3b

mCherry short expo

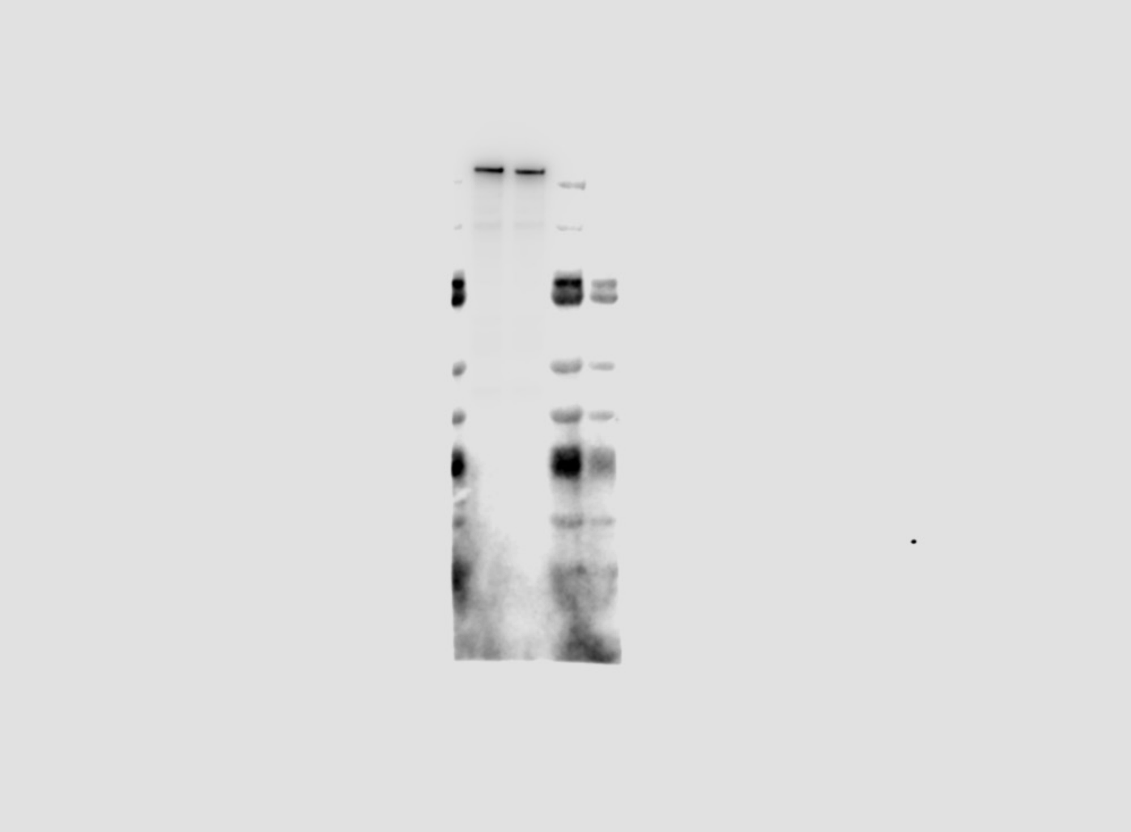

mCherry long expo

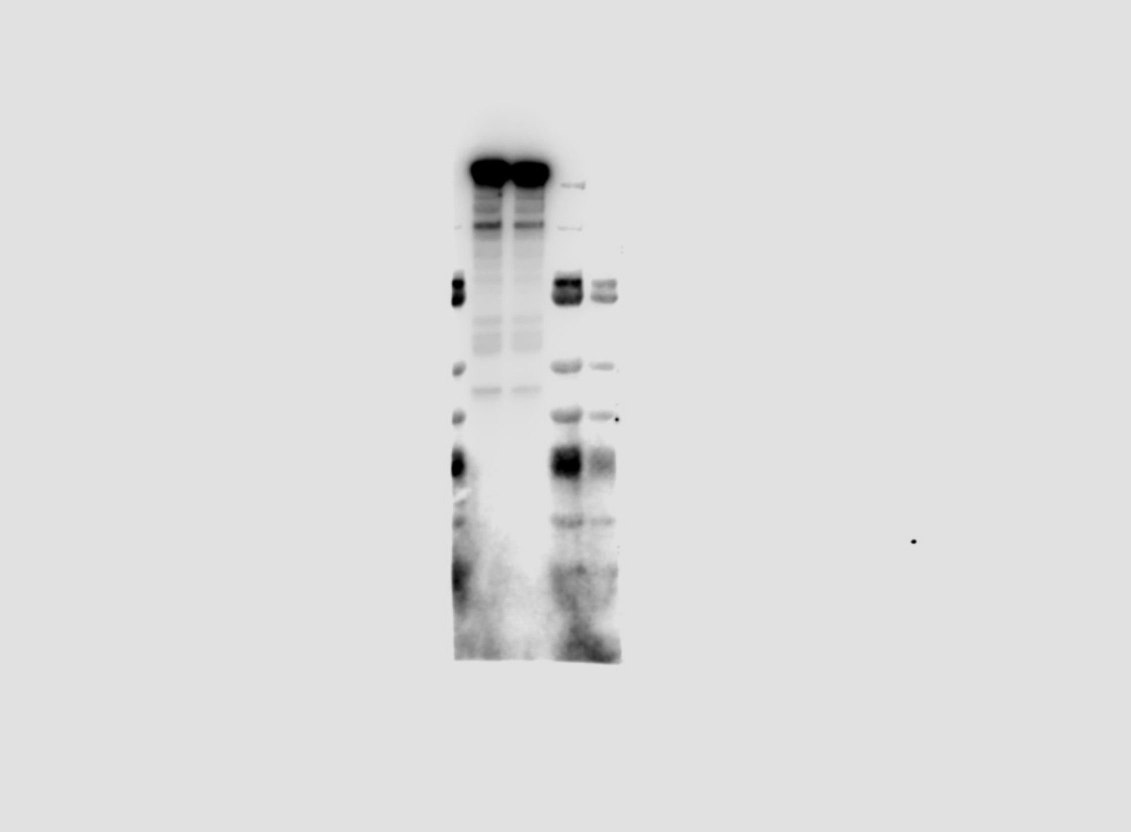

St. Jude - Confidential

Extended Data Fig3b

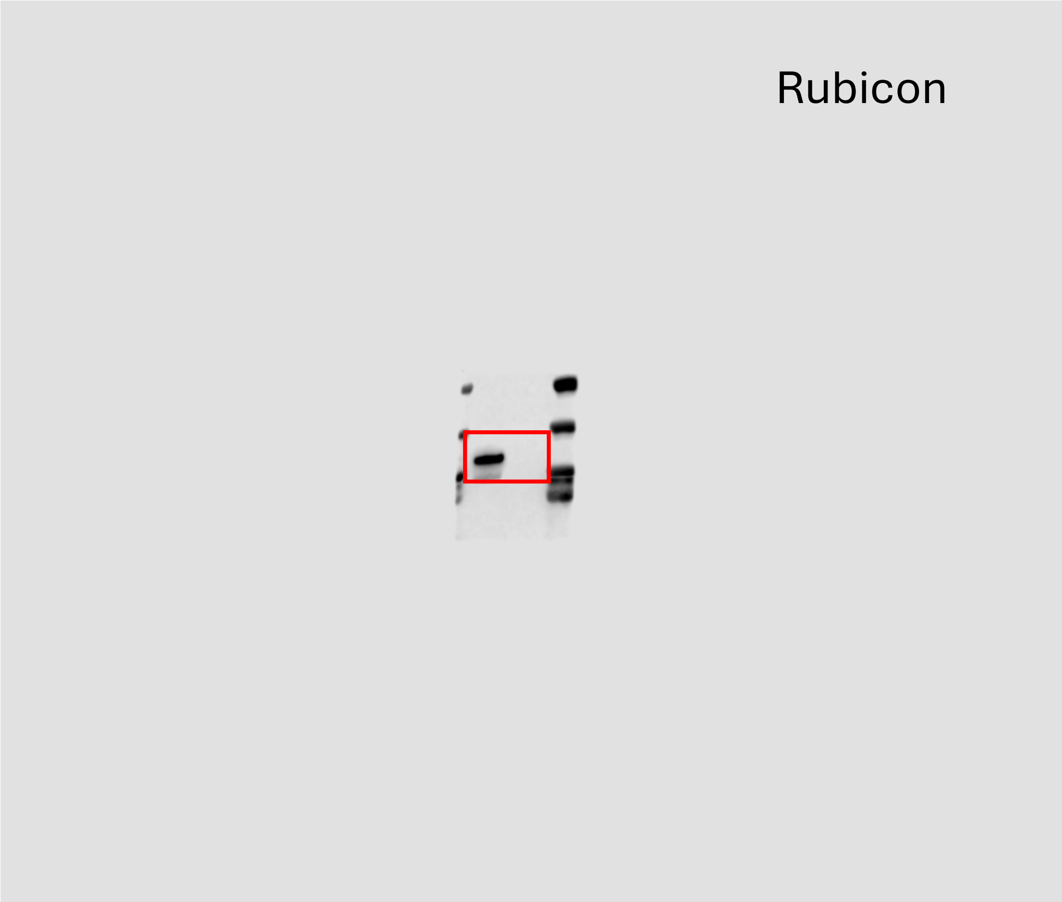

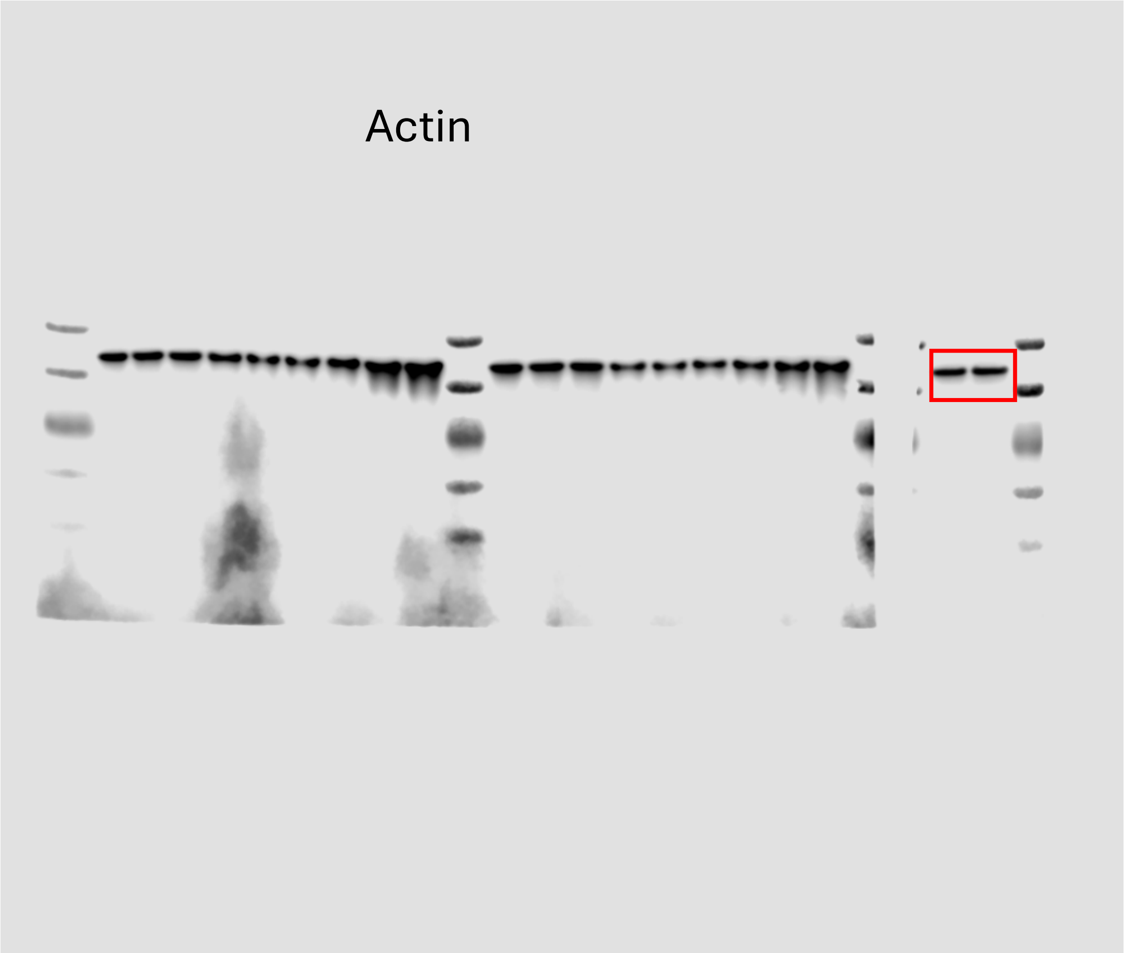

Extended Data fig. 5a

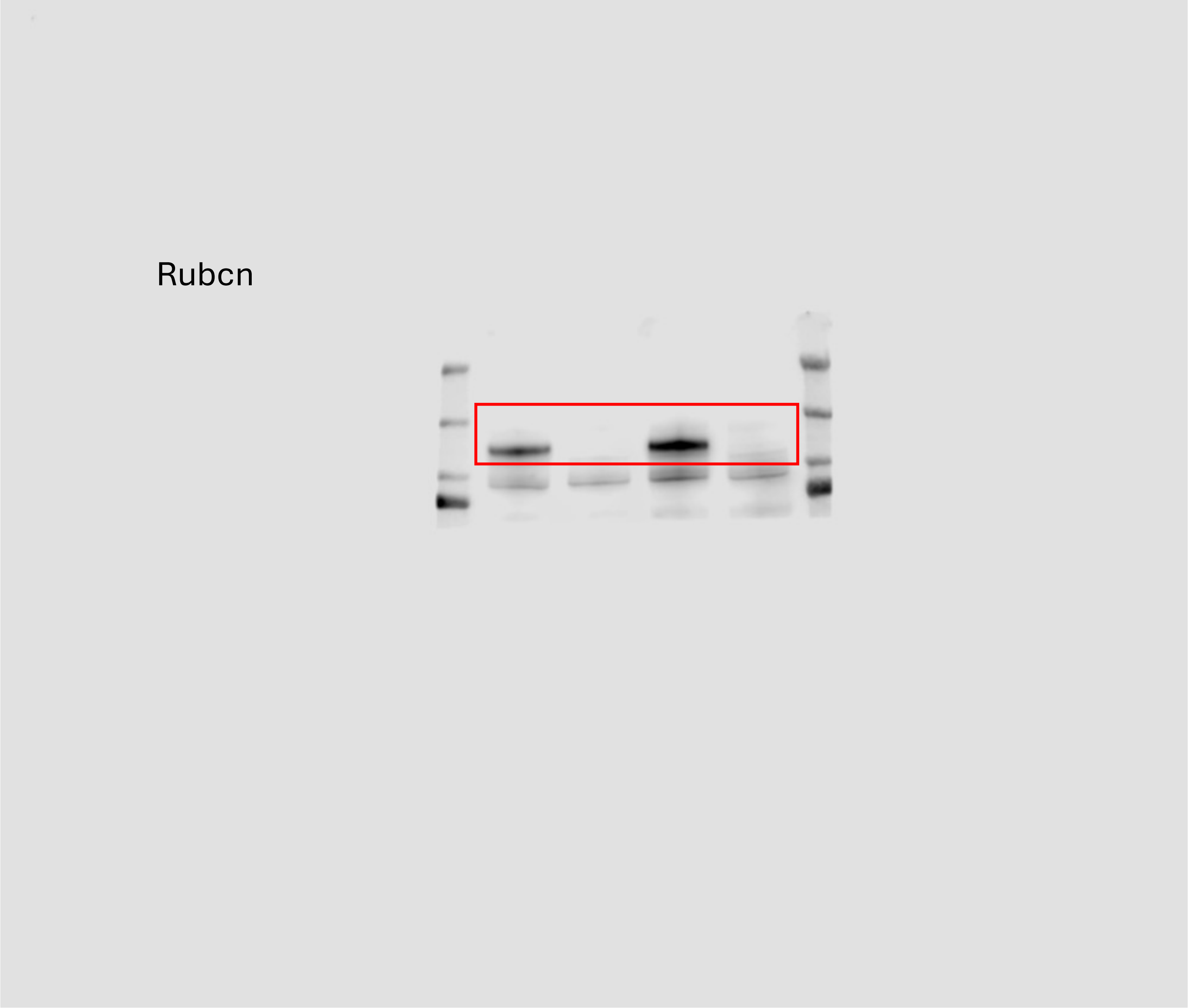

Extended Data fig. 5a

Actin

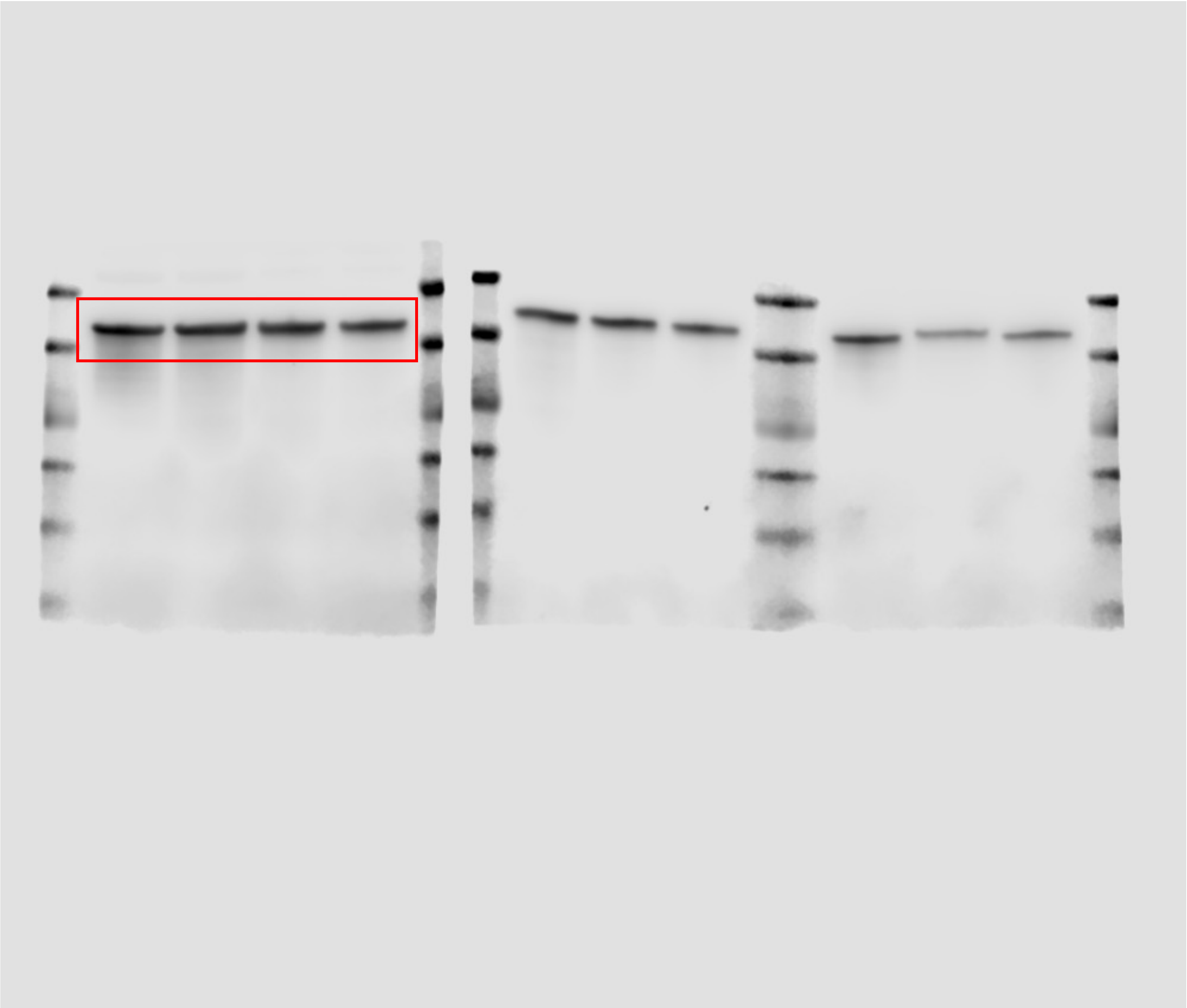

Extended data fig. 5b

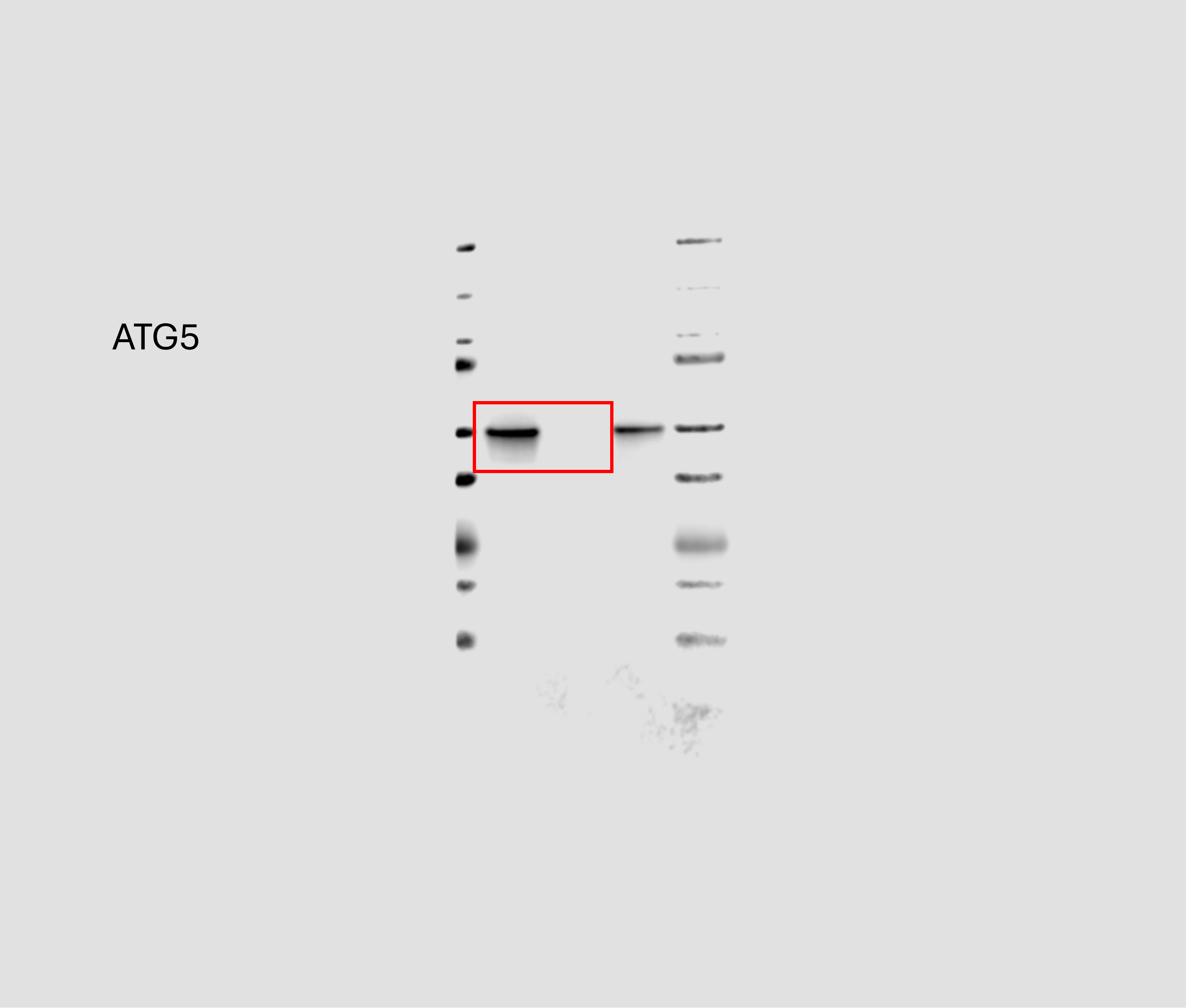

Extended data fig. 5b

Actin

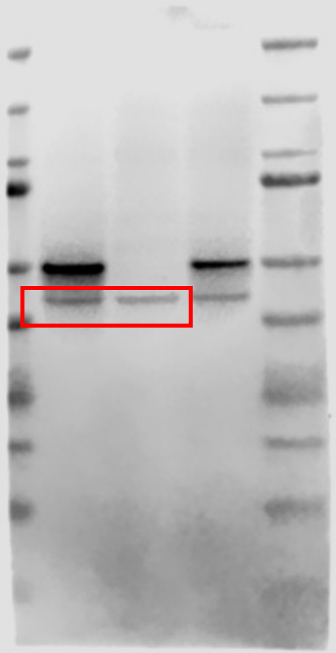

Extended Data Fig. 5e

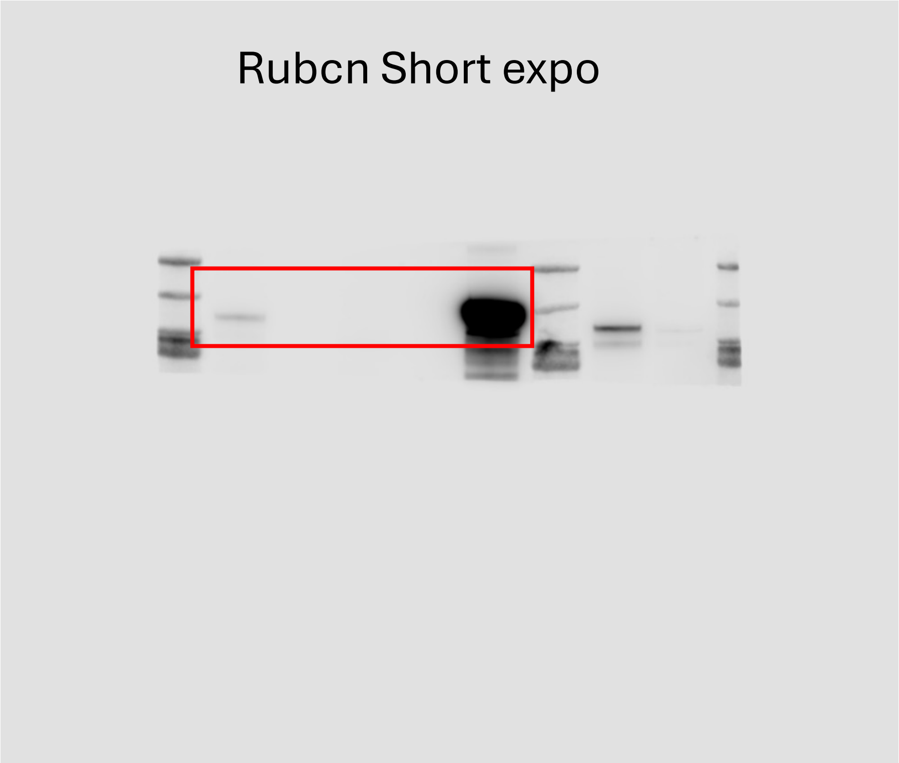

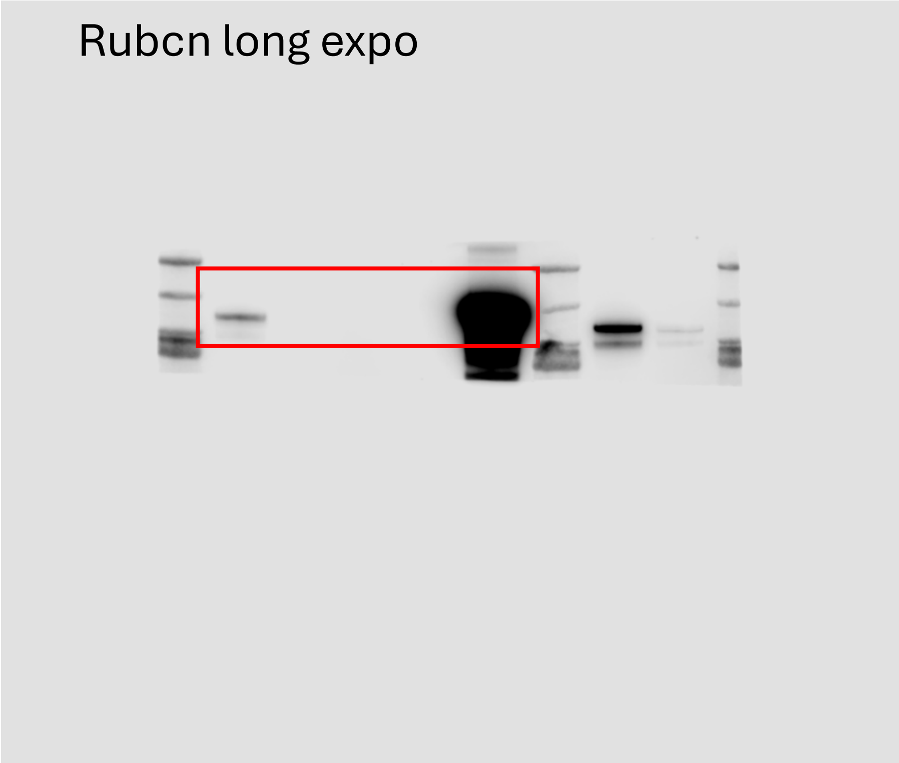

Extended Data Fig. 5e

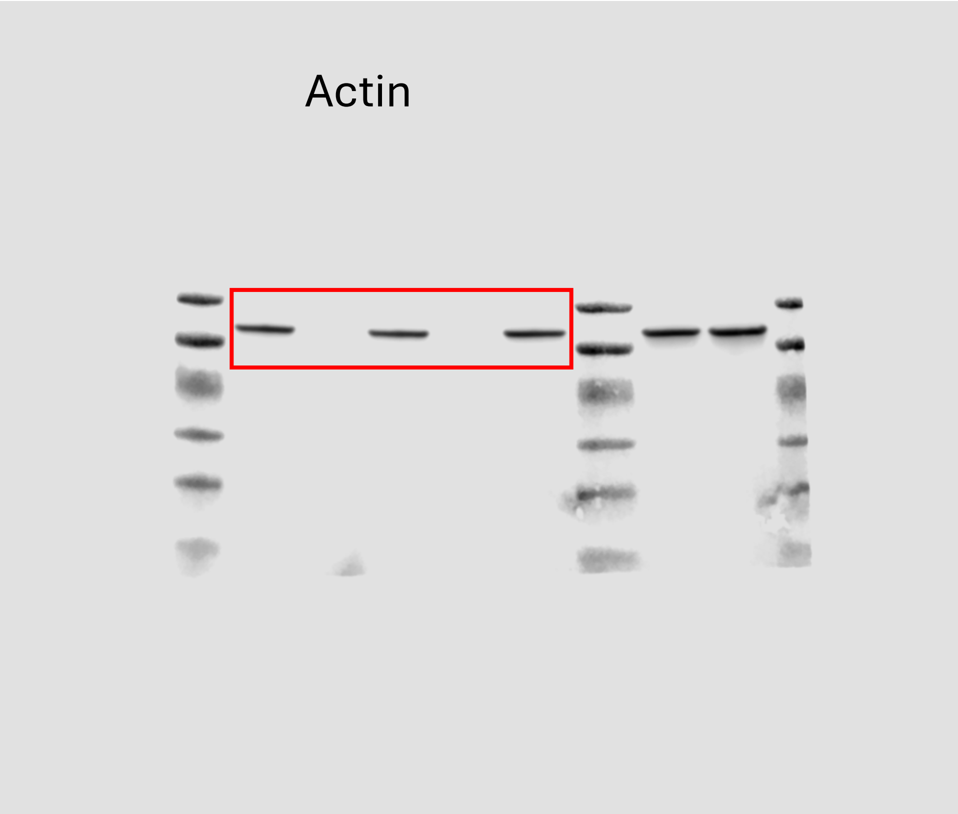

Extended Data Fig5f

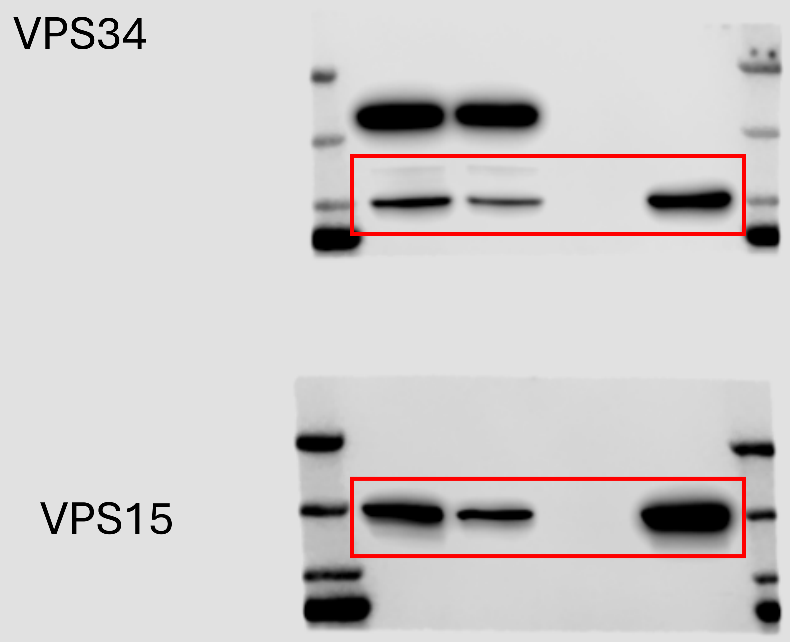

Extended Data Fig5f

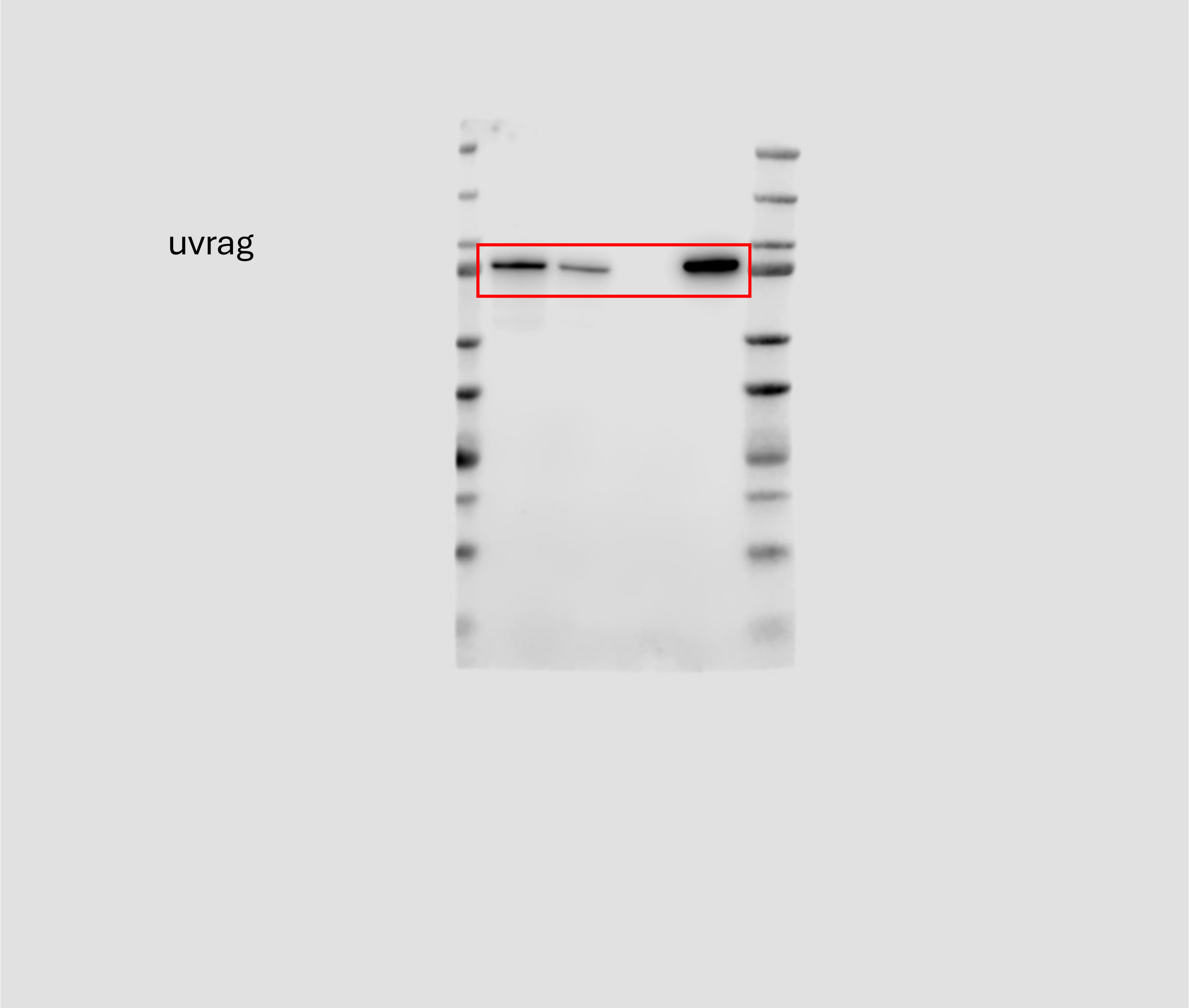

Extended Data Fig5f

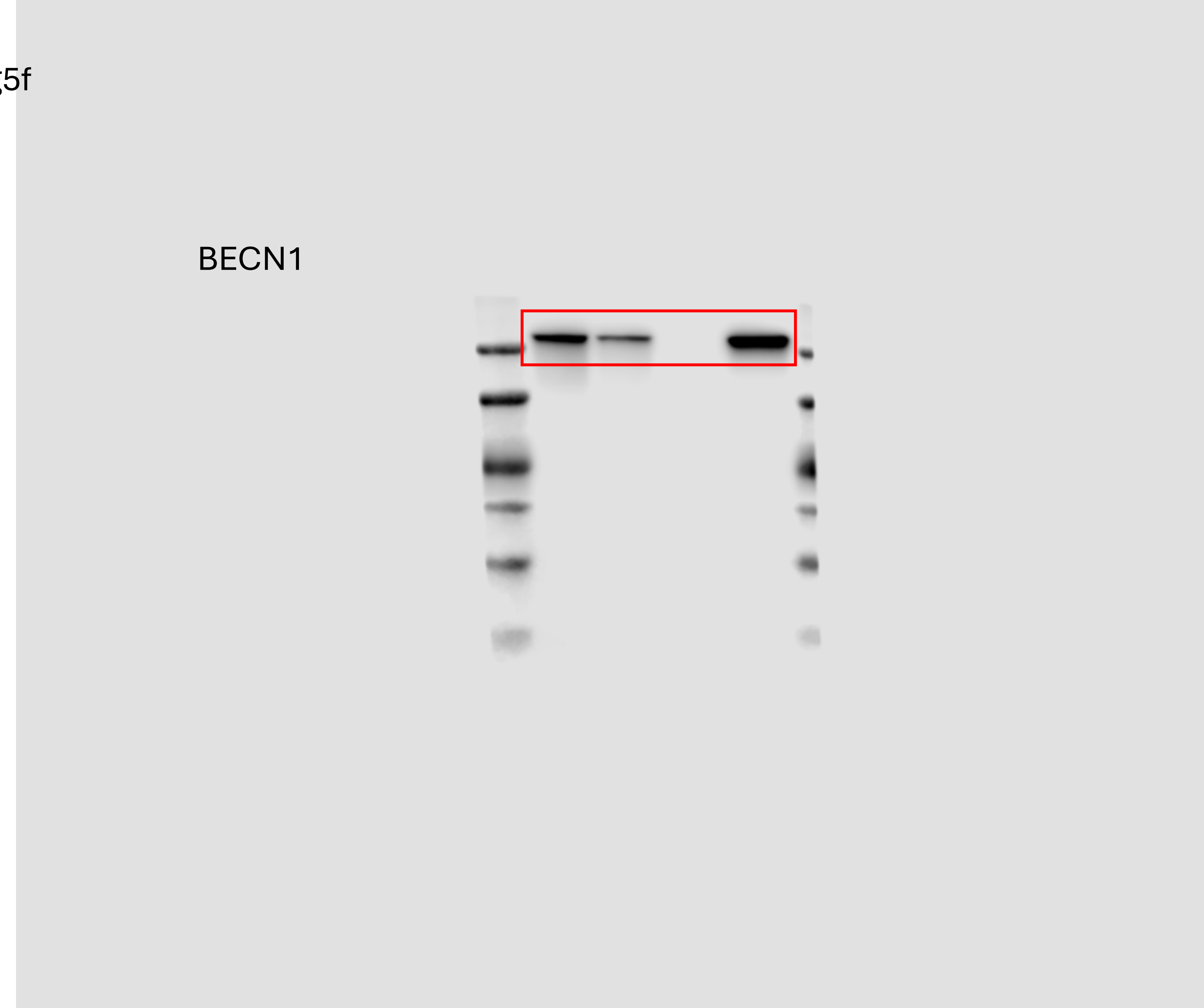

Extended Data Fig5f

Rubcn short exposure

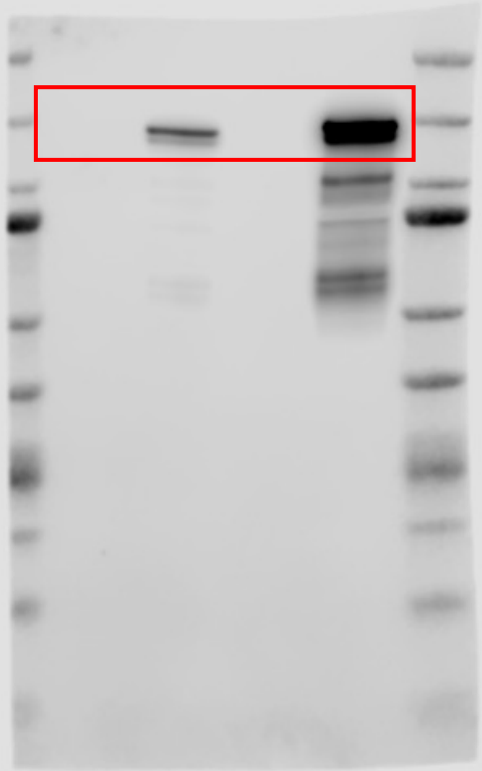

Extended Data Fig5f

Rubcn long exposure

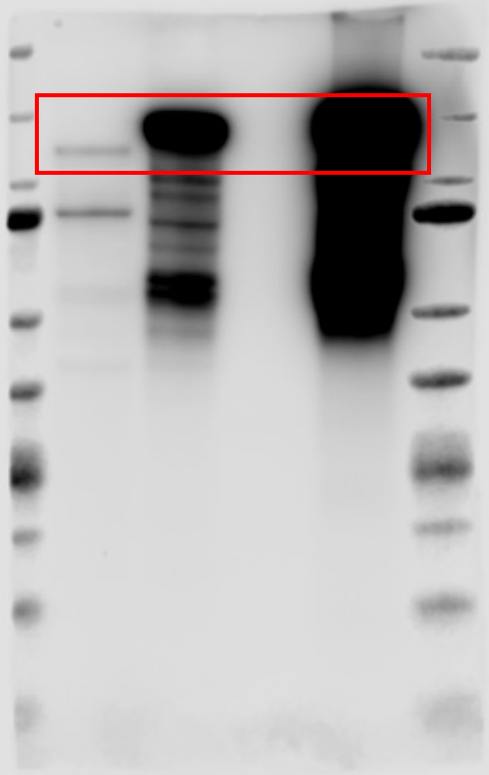

Extended Data Fig5f

Actin

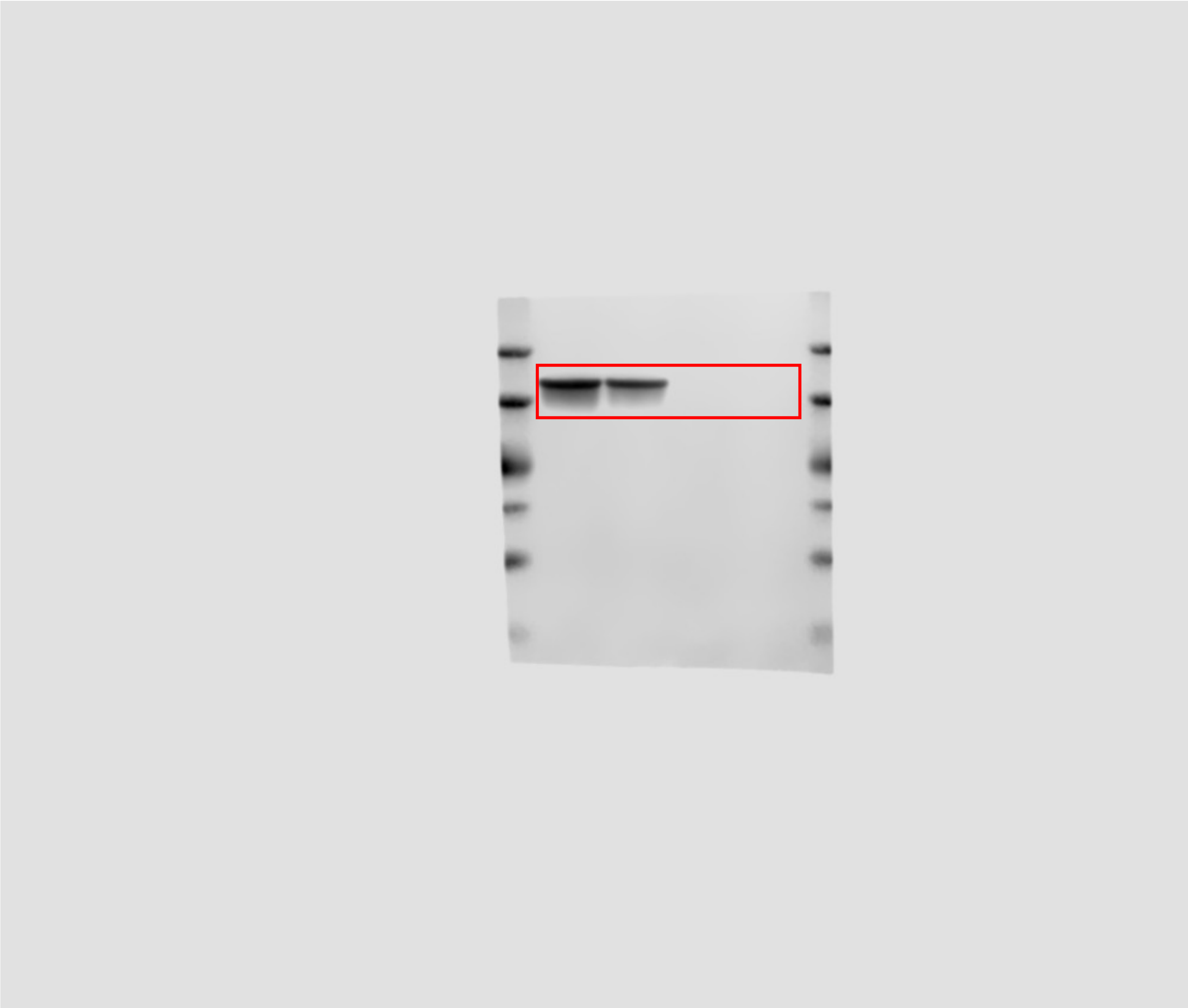

Extended Data Fig. 5h

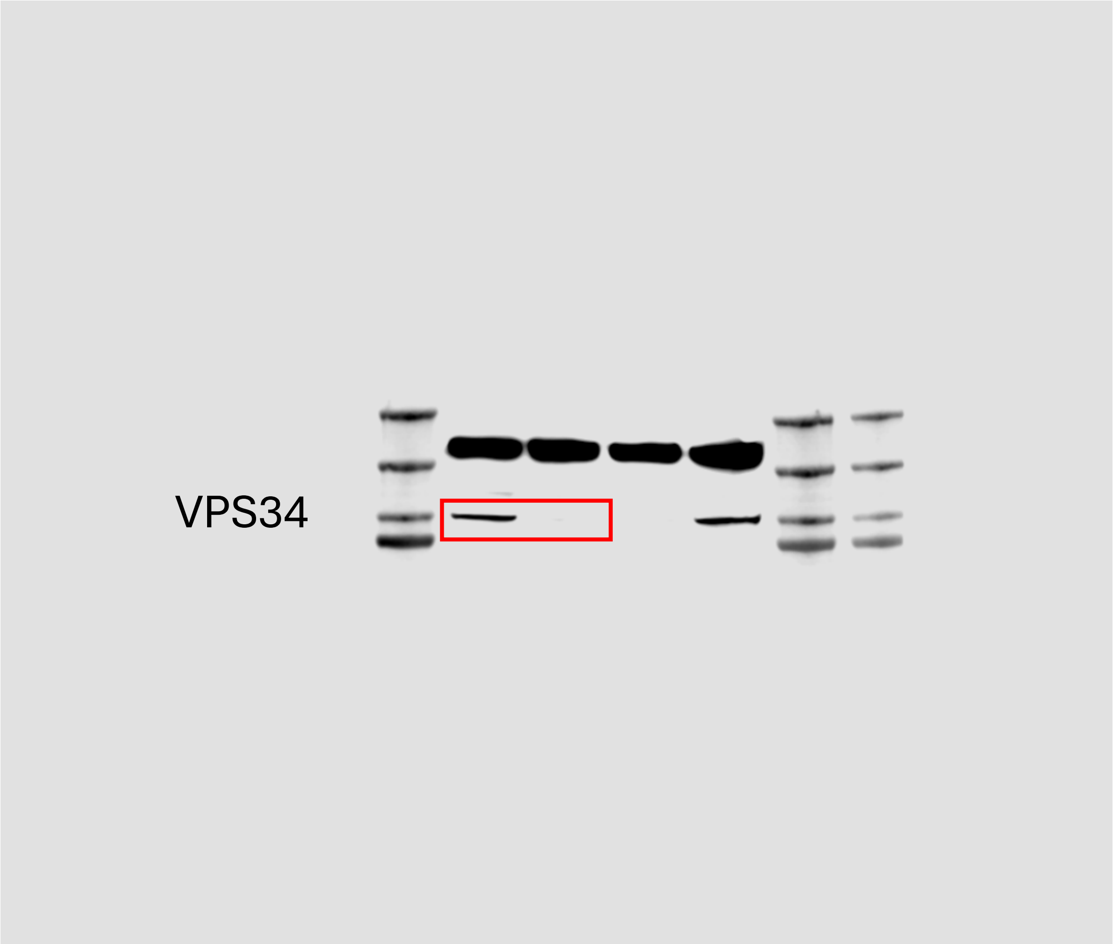

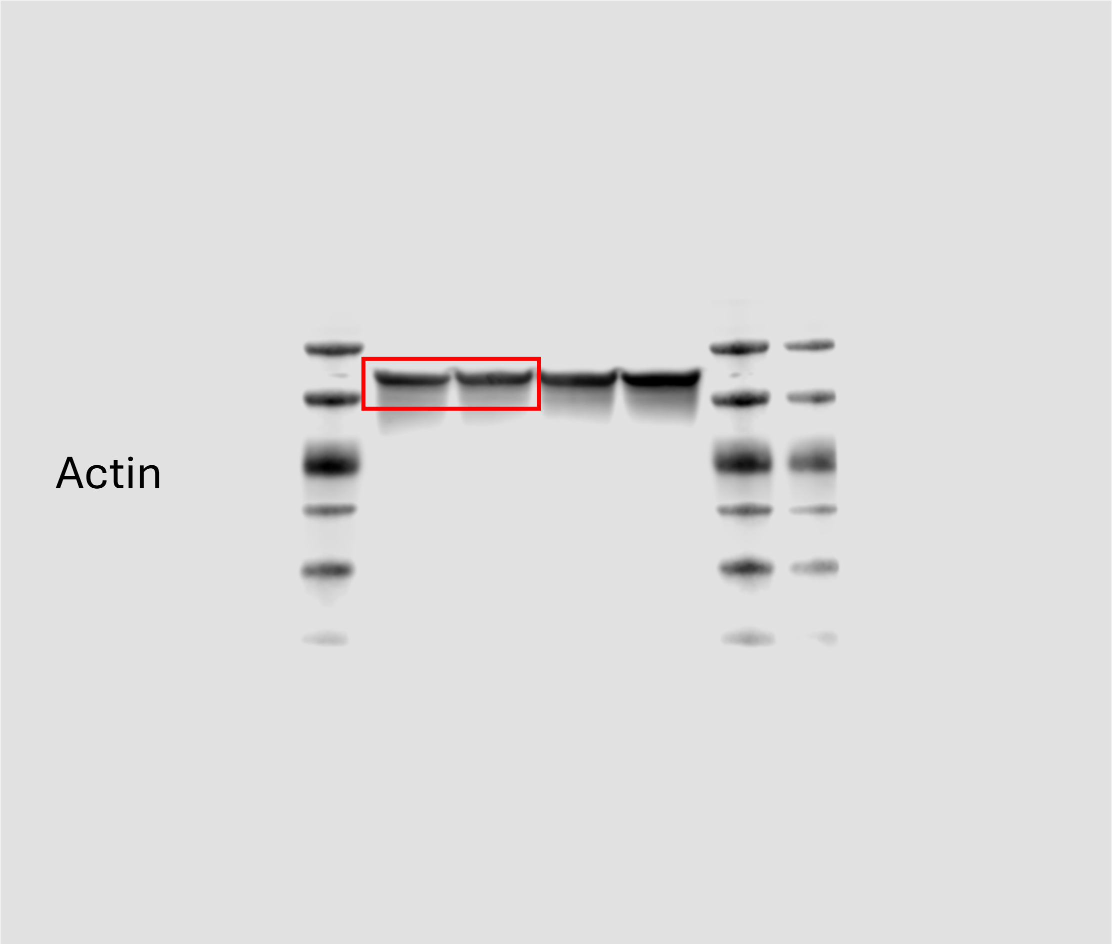

Extended Data Fig. 5h

ATG14

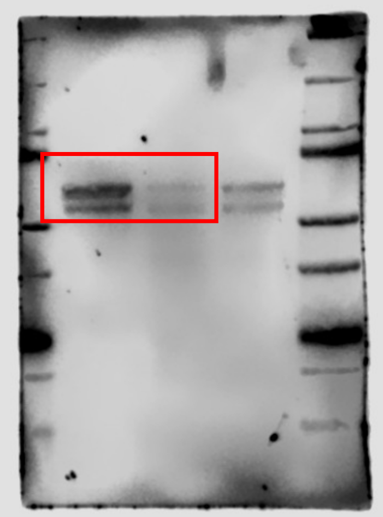

Actin

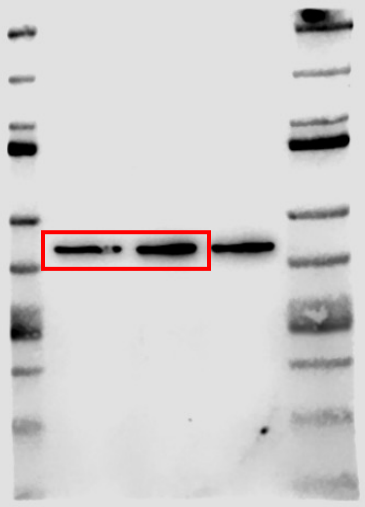

Extended Data Fig.6e

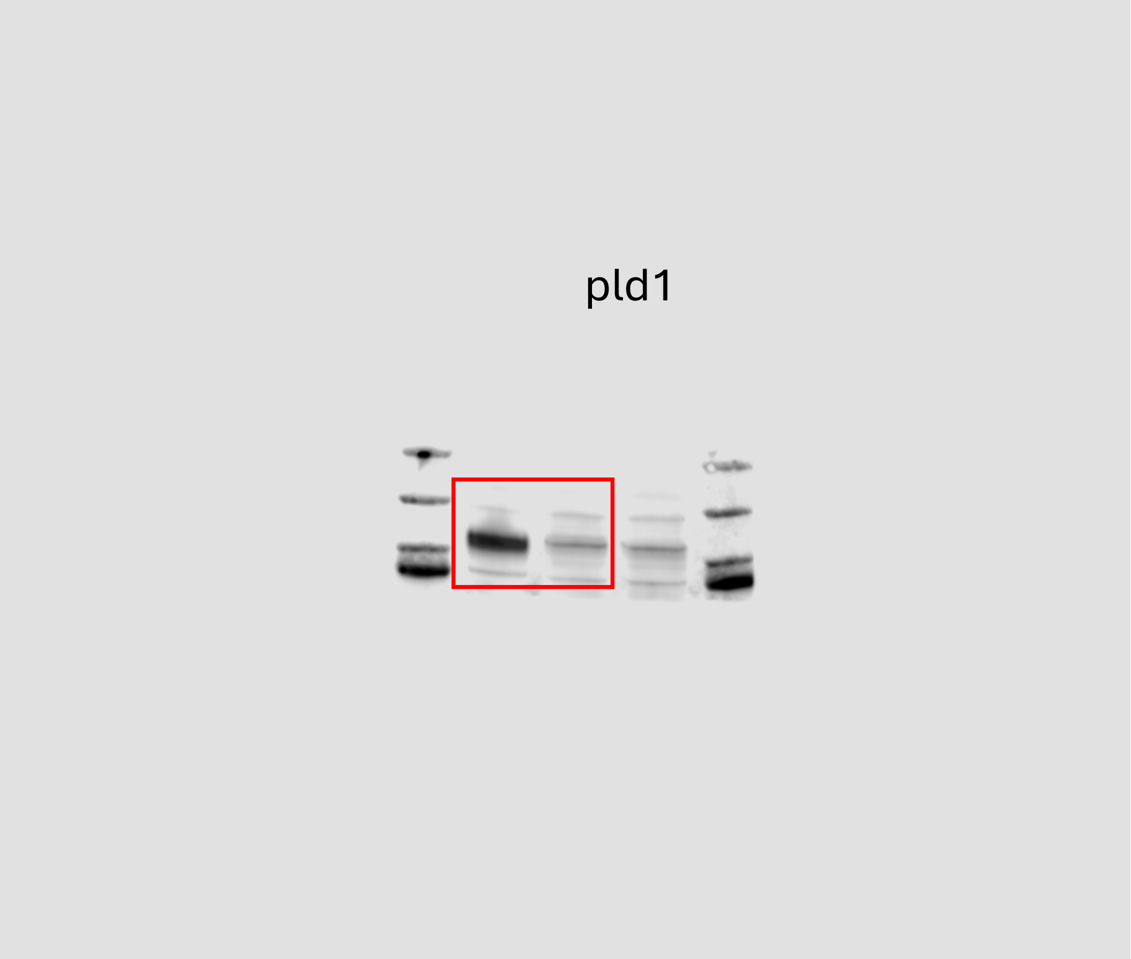

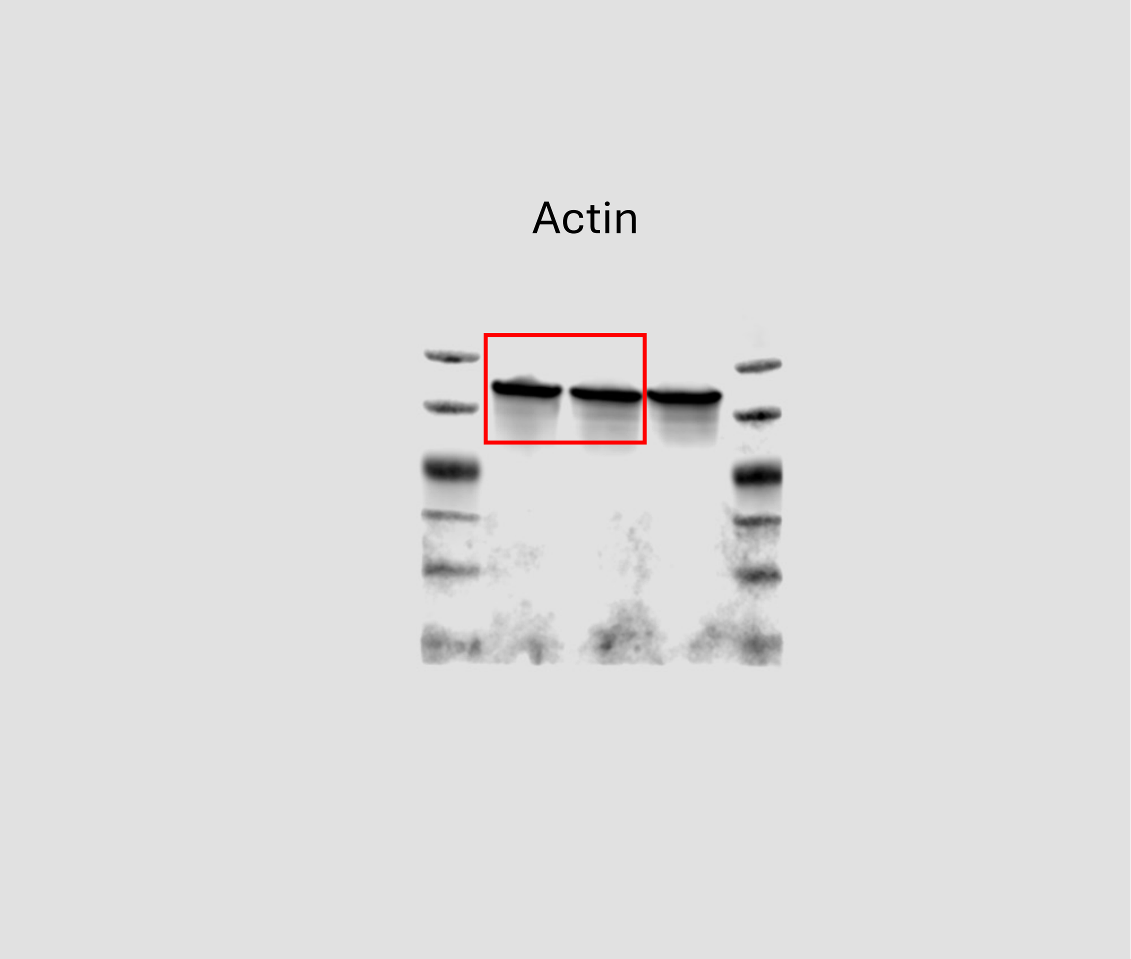

St. Jude - Confidential
